# Machine-learning-assisted high-throughput identification of potent and stable neutralizing antibodies against all four dengue virus serotypes

**DOI:** 10.1038/s41598-024-67487-8

**Published:** 2024-07-26

**Authors:** Piyatida Natsrita, Phasit Charoenkwan, Watshara Shoombuatong, Panupong Mahalapbutr, Kiatichai Faksri, Sorujsiri Chareonsudjai, Thanyada Rungrotmongkol, Chonlatip Pipattanaboon

**Affiliations:** 1 Department of Microbiology, Faculty of Medicine, Khon Kaen University, Khon Kaen, 40002 Thailand; 2 Research and Diagnostic Center for Emerging Infectious Diseases, Khon Kaen University, Khon Kaen, 40002 Thailand; 3 Modern Management and Information Technology, College of Arts, Media and Technology, Chiang Mai University, Chiang Mai, 50200 Thailand; 4 Center for Research Innovation and Biomedical Informatics, Faculty of Medical Technology, Mahidol University, Bangkok, 10700 Thailand; 5 Department of Biochemistry, Faculty of Medicine, Khon Kaen University, Khon Kaen, 40002 Thailand; 6 Center of Excellent in Biocatalyst and Sustainable Biotechnology, Department of Biochemistry, Faculty of Science, Chulalongkorn University, Bangkok, 10330 Thailand

**Keywords:** Computational biology and bioinformatics, Drug discovery, Microbiology

## Abstract

Several computational methods have been developed to identify neutralizing antibodies (NAbs) covering four dengue virus serotypes (DENV-1 to DENV-4); however, limitations of the dataset and the resulting performance remain. Here, we developed a new computational framework to predict potent and stable NAbs against DENV-1 to DENV-4 using only antibody (CDR-H3) and epitope sequences as input. Specifically, our proposed computational framework employed sequence-based ML and molecular dynamic simulation (MD) methods to achieve more accurate identification. First, we built a novel dataset (n = 1108) by compiling the interactions of CDR-H3 and epitope sequences with the half maximum inhibitory concentration (IC50) values, which represent neutralizing activities. Second, we achieved an accurately predictive ML model that showed high AUC values of 0.879 and 0.885 by tenfold cross-validation and independent tests, respectively. Finally, our computational framework could be applied to filter approximately 2.5 million unseen antibodies into two final candidates that showed strong and stable binding to all four serotypes. In addition, the most potent and stable candidate (1B3B9_V21) was evaluated for its development potential as a therapeutic agent by molecular docking and MD simulations. This study provides an antibody computational approach to facilitate the high-throughput identification of NAbs and accelerate the development of therapeutic antibodies.

## Introduction

Dengue virus (DENV), which has four antigenically distinct serotypes (DENV-1 to DENV-4), has worldwide spread and causes 400 million infections/year, with 100 million cases of mild to severe dengue disease (dengue fever, dengue haemorrhagic fever, or dengue shock syndrome) and 20,000 deaths^1^. To date, there is neither a fully effective, licenced dengue vaccine nor an approved therapeutic agent due to the phenomenon of antibody-dependent enhancement (ADE), which is a pathological immune response to subsequent infection with a different serotype, leading to severe disease^2^. ADE occurs when non-neutralizing or sub-neutralizing levels of antibodies from a previous dengue infection or vaccination bind to a different serotype of dengue virus during a subsequent infection. Recent updates show that various approved dengue vaccines demonstrate differing efficacy rates across each serotype, typically ranging from approximately 60% and 80% in clinical trials involving both immune and non-immune participants to dengue^3^. To mitigate the risk of ADE, dengue vaccine or therapeutic candidates must induce strong and balanced immune responses against all four dengue serotypes.

DENV particles are composed of seven nonstructural proteins (NS1, NS2A, NS2B, NS3, NS4A, NS4B, NS5) and three structural proteins (E, C, prM/M). Dengue envelope (E) protein is the principal target for potent broadly neutralizing antibodies (NAbs), containing neutralizing epitopes on EDII (envelope domain II with fusion loop), EDI-EDII (interdomain), EDIII (envelope domain III), and EDE (envelope dimer epitope)^4–7^. The overall structure of the E protein is conserved across all four DENV serotypes, exhibiting 60–70% amino acid sequence identity^8^. Developing cross-reactive neutralizing therapeutic antibodies or vaccines against all serotypes requires navigating the structural variability of the E protein and identifying conserved epitopes that induce protective immunity without enhancing infection. The fusion loop, a conserved region situated within EDII, plays a vital role in the viral fusion process with host cells and may potentially trigger ADE^8^. Variations in the sequence and structure of the EDII among serotypes impact antibody binding, neutralization efficacy, and ADE, thereby impacting the design of both therapeutics and vaccines. Addressing these challenges emphasizes the critical importance of gaining a thorough understanding of the structure of DENV and the interactions between antibodies and antigens. This understanding is crucial for refining strategies aimed at developing vaccines and therapeutics.

Over the past three decades, there have been many attempts to develop NAbs against E proteins of all four serotypes, known as cross-neutralizing antibodies, with half maximum inhibitory concentration (IC50) values (or neutralizing activities) less than 10 µg/mL to reduce the effects of ADE as promising therapeutic agents for dengue infection^9,10^. Recent studies on therapeutic antibodies have established a threshold of IC50 ≤ 10 µg/mL to distinguish between neutralizing (IC50 ≤ 10 µg/mL) and nonneutralizing (IC50 > 10 µg/mL) activities^11,12^. One of our antibody candidates, 1B3B9, targets the fusion loop and effectively neutralizes all four dengue serotypes. However, it exhibits varying levels of neutralizing activity (ranging from 0.125 to 3 ug/ml) against different serotypes, which could potentially lead to ADE^6,13^. To tackle this challenge, we aim to mutate this antibody and apply a computational framework to enhance its effectiveness.

The design of antibodies for the treatment of various diseases, such as DENV infectious diseases, requires two structural parts: fragment antigen-binding (Fab) and constant fragment crystallizable (Fc) regions^14^. The Fab region contains the hypervariable regions or complementary determining regions (CDRs) CDR-H1, CDR-H2, and CDR-H3 on each heavy chain and CDR-L1, CDR-L2, and CDR-L3 on each light chain. CDR-H3 is the most hypervariable region responsible for binding and neutralizing activities that should be considered for antibody-specified epitope prediction^15^. Antibody design and prediction (screening) is the most important step to limit laborious, time-consuming, and expensive laboratory work in the process of therapeutic antibody discovery and development. Recently, the integration of experimental (in vitro and in vivo) and computational (in silico) methods has assisted and enriched the effective generation of therapeutic antibodies due to the advancement of experimental studies, sequence data, structural data, and computational approaches, especially machine learning (ML) and molecular dynamics (MD) simulation techniques^15^.

ML and MD can be used to detect and predict important patterns of epitopes on antigens, paratopes on antibodies, or paratope–epitope interactions and enable us to develop novel therapeutic antibodies quickly to combat emerging and reemerging diseases^15,16^. Most of the computational algorithms for antibody screening that showed high accuracy involved the use of complex methods and structural data^17,18^. Simple, sequence-based ML methods that can be used to screen for antibodies with desired properties have been reported for only some diseases, such as cancers^19,20^, HIV infection^21,22^, and SARS-CoV-2 infection^23^. Previous prediction approaches for dengue NAbs have used only ML-based^24,25^ or MD-based^26^ methods, which have different advantages in high-throughput prediction for antibody screening or highly efficient prediction for antibody developability, respectively^24,27^. None of the previous approaches used the dengue antibody dataset containing full-length CDR-H3 sequences and epitope sequences together with the in vitro IC50 values. Additionally, there is no complete in silico screening approach for cross-neutralizing antibodies against the four DENV serotypes^24,28^.

In this study, we developed an computational framework, incorporating a sequence-based ML method and a simple MD method, to accurately identify potent and stable neutralizing antibodies against all four dengue serotypes. The development process in our framework consists of four major steps: (1) dataset preparation, (2) feature extraction and ML analysis, (3) ML screening, and (4) MD screening. The major contributions from this process can be summarized as follows. First, we comprehensively generated a novel sequence-based dataset with IC50 activities from available experimental results over the last three decades. Second, we developed a computational framework, which is the first integrative approach (ML and MD methods) to identify potent and stable antibodies against DENV-1 to DENV-4. We compared and demonstrated the performance of three different encoding schemes in conjunction with ten ML algorithms to select the best performing model and capture the crucial information on NAbs. We then applied this computational framework to screen several million unseen antibodies into two final NAb candidates. Finally, we identified the most potent and stable 1B3B9_V21 antibody as a promising therapeutic agent against dengue infection by molecular docking and MD analysis.

## Results

### Computational framework overview and dataset analysis

**Figure 1:**
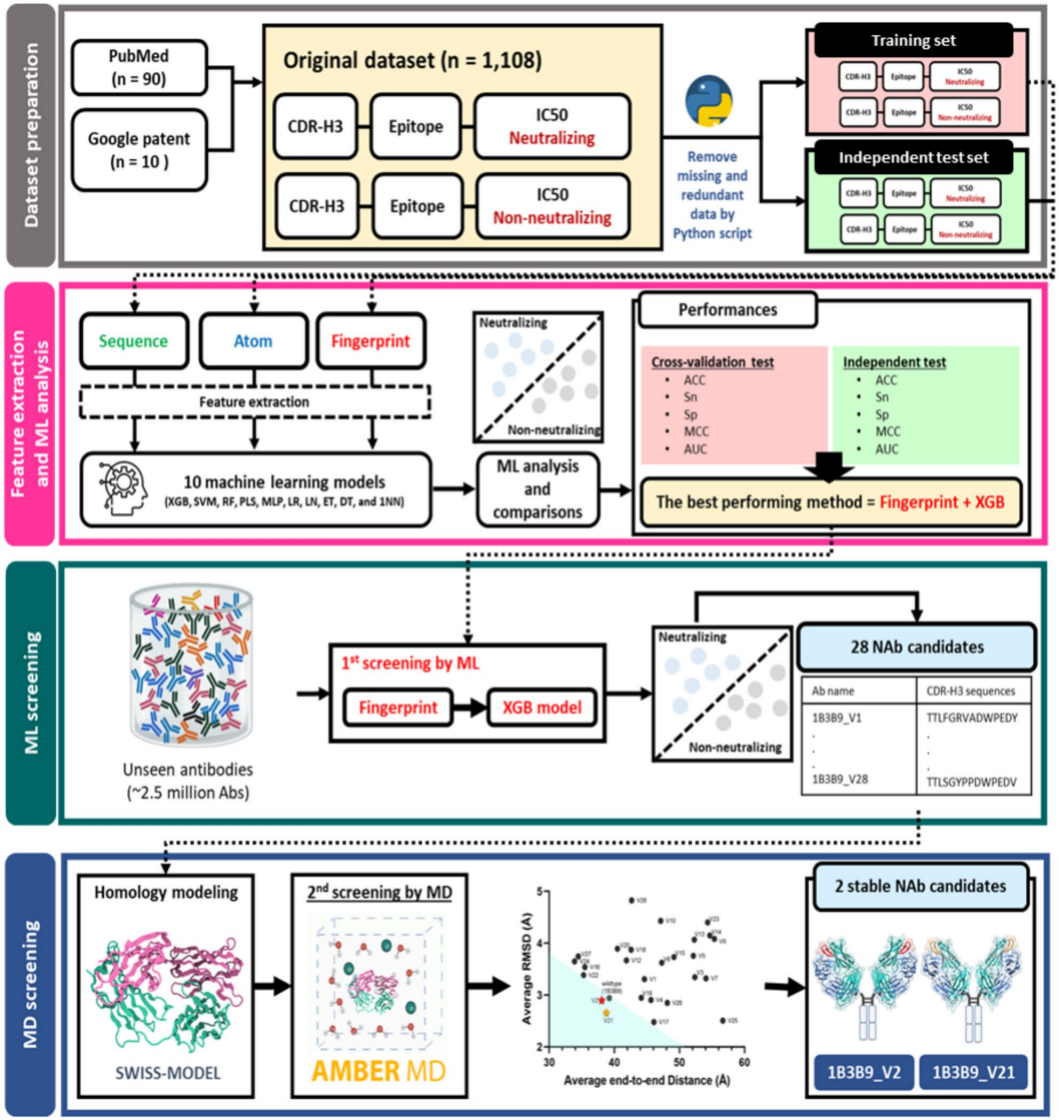
Overview of the computational framework. We generated a dataset of CDR-H3 epitope IC50 values from the PubMed and Google patent databases (n = 100 publications). The computational framework includes 4 steps: (1) dataset preparation (n = 1,108 interactions), (2) feature extraction (n = 3 methods: sequence-based, atom-based, fingerprint-based feature descriptors) and ML analysis (n = 10 models: XGB, SVM, RF, PLS, MLP, LR, LN, ET, DT, and 1NN), (3) ML screening (XGB model), which provided 28 potential NAb candidates, and (4) MD screening, which provided 2 stable NAb candidates (1B3B9_V2 and 1B3B9_V21).

We designed a novel in silico prediction framework integrating sequence-based ML and simple MD methods to screen high-throughput antibody variants for potent and stable NAb candidates against four serotypes of DENV. The computational framework overview consists of four steps: dataset preparation, feature extraction and ML analysis, ML screening, and MD screening (Fig. 1). In the dataset preparation, we collected CDR-H3 sequences, epitope sequences, and experimental IC50 values from well-defined PubMed and Google patent databases during 1992–2022 (n = 100 publications; Supplemental Table 1). Missing and redundant data were not included in our dataset. All sequences were paired to form a total of 1108 antibody–antigen interactions and further labelled according to IC50 values as the neutralizing class (positive dataset; IC50 ≤ 10 μg/mL; n = 554) and the nonneutralizing class (negative dataset; IC50 > 10 μg/mL; n = 554) (Table 1).

**Table 1:** Details of the benchmark dataset used for ML analysis.

Dataset (n = 1108)	Benchmark dataset	
	Positive (neutralizing)	Negative (nonneutralizing)
Training set	443	443
Independent test set	111	111
Total	554	554

**Figure 2:**
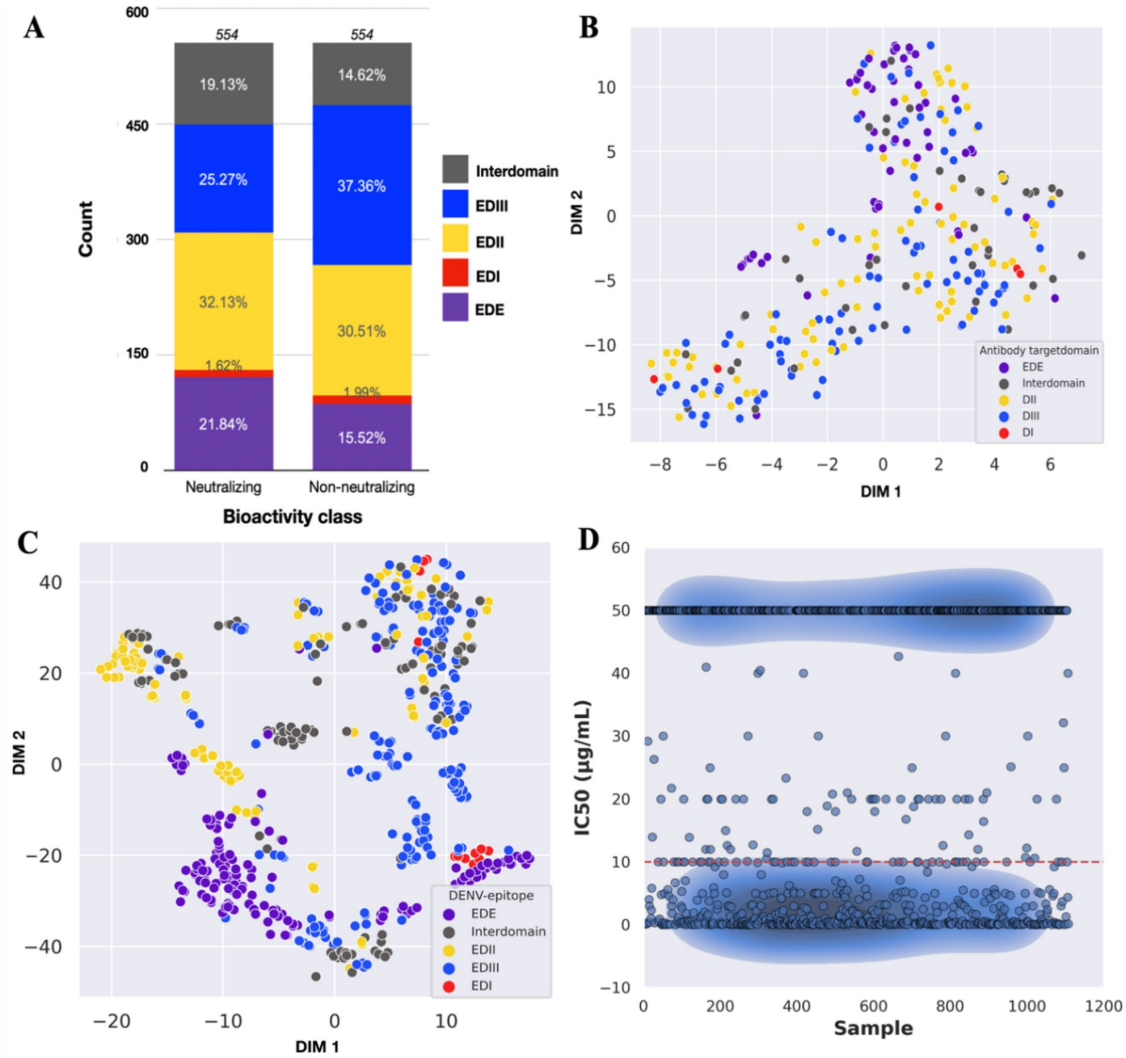
Visualization of the input dataset. We visualized the input dataset before feature extraction and ML analysis as follows: (**A**) Numbers of neutralizing (n = 554) and nonneutralizing (n = 554) interactions based on target epitope domains are shown in a bar plot. EDI is envelope domain I. EDII is envelope domain II. EDIII is envelope domain III. EDI-EDII is interdomain. EDE is the envelope dimer epitope domain. (**B**) Diversity of CDR-H3 antibody sequences based on target domains including EDI, EDII, EDIII, interdomain, and EDE by t-SNE (n = 306; perplexity = 30, learning rate = 100), (**C**) Diversity of epitope sequences based on target domains including EDI, EDII, EDIII, interdomain, and EDE by t-SNE (n = 609; perplexity = 30, learning rate = 100), and (**D**) Distribution of IC50 values of each antibody–antigen interaction by scatter plot (n = 1,108; cut-off value for neutralizing class ≤ 10 μg/ml).

Overall, we found that the antibody–antigen interactions in the neutralizing (N) and nonneutralizing (NN) classes had binding residues on EDII (N = 32.13%, NN = 30.51%), EDIII (N = 25.27%, NN = 37.36%), EDE (N = 21.84%, NN = 15.52%), interdomain (N = 19.13%, NN = 14.62%), and EDI (N = 1.62%, NN = 1.99%) (Fig. 2A). We randomly divided our balanced dataset into a training dataset (80%, n = 443) and an independent dataset (20%, n = 111) (Table 1). To visualize the diversity of our training data, we separately analysed the sequence-extracted input of CDR-H3 and epitope sequences in the t-distributed stochastic neighbour embedding (t-SNE) plot and labelled them with epitope domains. We found that CDR-H3 sequences are diverse, as illustrated in Fig. 2B. In contrast, the epitope sequences in all domains are well clustered in several areas (Fig. 2C). We also evaluated the IC50 labels of all input interactions and found that the IC50 cut-off value representing a high neutralizing antibody level (IC50 ≤ 10 μg/ml) can be used to explicitly divide our dataset into neutralizing and nonneutralizing classes (Fig. 2D).

### Analysis and selection of the best ML-performing method

**Figure 3:**
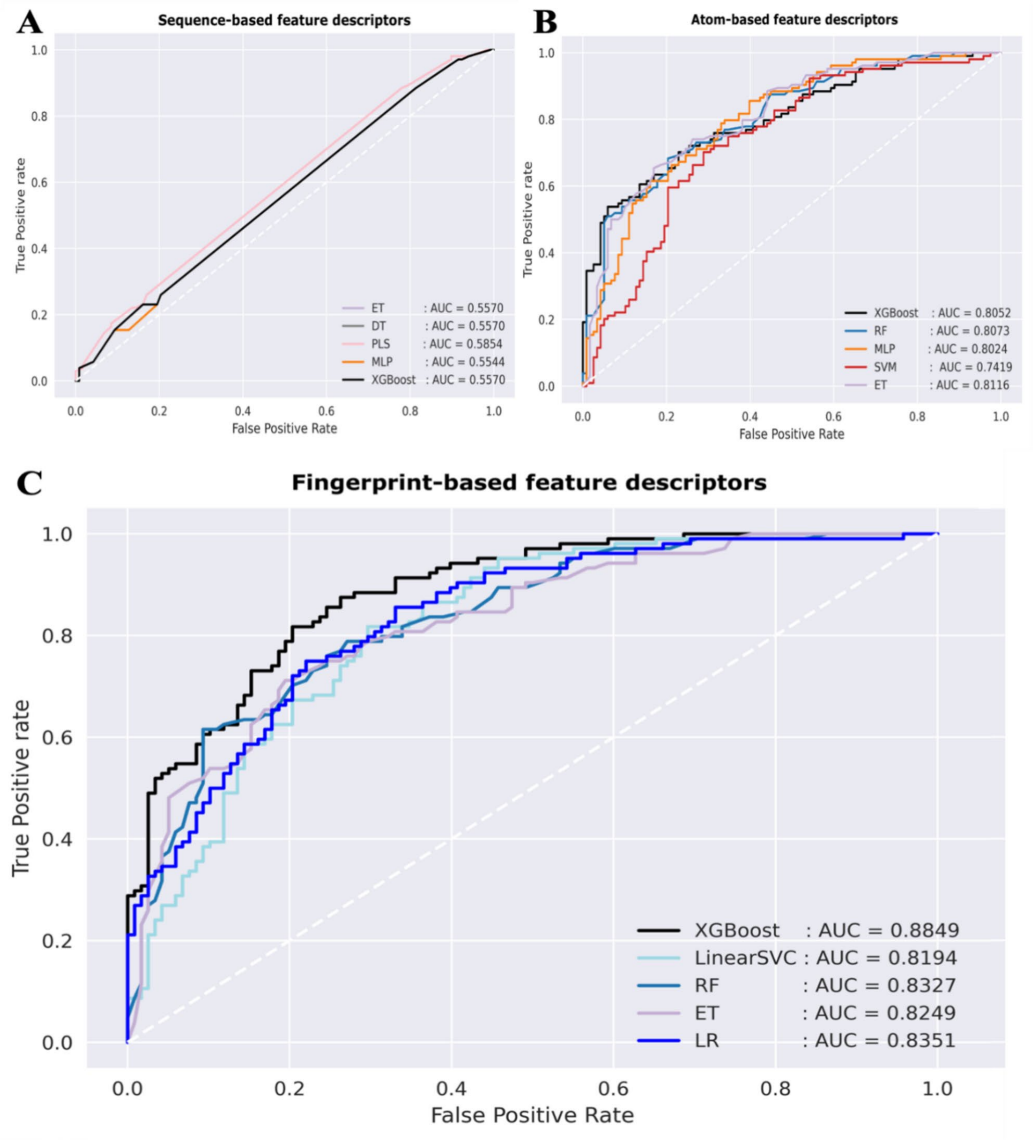
Performances of the top five ML models in the three encoding methods. We illustrated the performances of the top five ML models in three encoding methods by ROC curves with the AUC values. (**A**) ML analysis using sequence-based features. (**B**) ML analysis using atom-based features. (**C**) ML analysis using fingerprint-based features. We found that the XGB model and fingerprint encoding method are the most suitable approaches for classifying neutralizing and nonneutralizing antibody classes.

**Figure 4:**
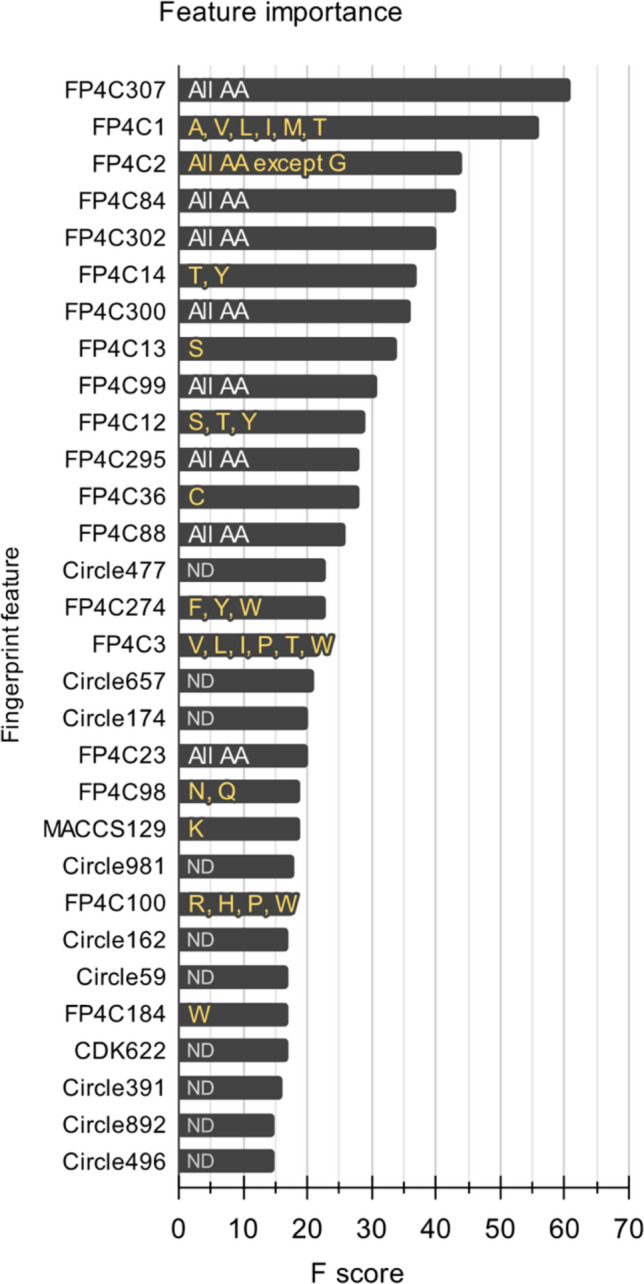
Top 30 important features with corresponding amino acids. We analysed our dataset and found the top 30 important features and corresponding amino acids of the neutralizing class from XGB model analysis. (All AA; all amino acids, ND; could not be determined, A; alanine, V; valine, L; leucine, I; isoleucine, M; methionine, T; threonine, G; glycine, Y; tyrosine, S; serine, C; cysteine, F; phenylalanine, W; tryptophan, N; asparagine, Q; glutamine, K; lysine, R; arginine, H; histidine, P; proline).

In this section, we describe a comparative experiment on a variant ML model developed by using three encoding methods and ten ML models. In the ML analysis, we performed tenfold cross-validation and independent tests on our dataset to demonstrate the prediction performance of all ML models (Supplemental Tables 6–8). To compare the ML performance for NAb prediction, we demonstrated the top five performing models with the highest AUC scores of each experiment in terms of five different matrices by tenfold cross-validation and independent tests (Table 2). We found that the AUCs of the top five ML models using the fingerprint-based encoding method were higher than those of other encoding methods (Table 2). Finally, we selected the best predictive approach of the XGB algorithm and fingerprint-based encoding method for the first-round screening of potential antibody candidates. This ML model yielded an ACC of 0.802, Sn of 0.788, Sp of 0.817, MCC of 0.604, and AUC of 0.885, as indicated by an independent test (Table 2). To confirm the interpretability of the XGB model, we illustrated the performance of this model and the other top five ML models of each method in ROC curves with AUC values of 0.554–0.885 by the independent test (Fig. 3A–C). The XGB model has an AUC of 0.885, which represents the strong ability of the XGB model to classify neutralizing or nonneutralizing antibodies (Fig. 3C). Furthermore, we determined the top 30 fingerprint features connecting with the corresponding amino acids, which are often found in the neutralizing class, via the F score plot to demonstrate the accuracy of those features (Fig. 4). The important features correlated with some amino acids (not all or nearly all amino acids) were considered unique features that might play a major role in the neutralization mechanism of dengue antibodies, including alcohol (amino acids S, T, and Y) and aromaticity (amino acids F, Y, and W) features (Fig. 4). However, there are some features (labelled as could not be determined; ND) that have not been reported to be related to amino acids and binding properties in antibody interactions.

**Table 2:** Top five ML algorithms based on three different feature encoding methods.

Feature set	Top performance model	Tenfold cross-validation					Independent test				
		ACC	Sn	Sp	MCC	AUC	ACC	Sn	Sp	MCC	AUC
Sequence-based descriptor	PLS	0.515	0.766	0.291	0.069	0.525	0.554	0.839	0.231	0.088	0.585
	MLP	0.528	0.832	0.236	0.085	0.524	0.514	0.186	0.885	0.099	0.567
	ET	0.526	0.837	0.227	0.078	0.531	0.536	0.805	0.231	0.044	0.557
	XGB	0.524	0.832	0.227	0.073	0.526	0.536	0.805	0.231	0.044	0.557
	DT	0.524	0.832	0.227	0.073	0.525	0.536	0.805	0.231	0.044	0.557
Atom-based descriptor	ET	0.738	0.751	0.729	0.478	0.808	0.725	0.712	0.740	0.451	0.812
	RF	0.737	0.737	0.740	0.474	0.805	0.725	0.670	0.740	0.451	0.812
	XGB	0.757	0.748	0.766	0.511	0.824	0.703	0.610	0.760	0.410	0.807
	MLP	0.669	0.635	0.703	0.335	0.728	0.680	0.602	0.856	0.372	0.805
	SVM	0.678	0.653	0.711	0.362	0.718	0.721	0.661	0.789	0.469	0.802
Fingerprint-based descriptor	XGB	0.815	0.791	0.842	0.630	0.879	0.802	0.788	0.817	0.604	0.885
	MLP	0.752	0.731	0.769	0.502	0.815	0.775	0.746	0.808	0.553	0.858
	SVM	0.809	0.788	0.831	0.618	0.868	0.779	0.754	0.808	0.561	0.852
	LR	0.758	0.741	0.775	0.514	0.825	0.748	0.695	0.808	0.503	0.837
	RF	0.791	0.769	0.814	0.583	0.854	0.757	0.729	0.789	0.516	0.833

ACC; Accuracy, Sn; Sensitivity, Sp; Specificity, MCC; Matthew’s correlation coefficient, AUC; Area under the ROC curve.

### Generation and screening of new antibody variants

We first generated new antibody variants (unseen antibodies) by applying single, double, and triple mutations to all amino acids of the CDR-H3 sequence (TTLSGYSADWPEDY) of the 1B3B9 neutralizing human monoclonal antibody, resulting in 2,529,794 CDR-H3 variants. These CDR-H3 variants were paired with the most cross-reactive epitopes of dengue virus (FL epitope residues; CCDRWCFCCK) as antibody–antigen sequences for feature extraction by the fingerprint-based method into the numerical input for the first ML screening of cross-neutralizing antibodies (potential candidates). To precisely predict the potential antibodies, we used the selected XGB model with a confidence score of 0.9900 for screening all antibody variants, and this ML screener filtered approximately 2.5 million variants to obtain 28 potential candidates. All 28 ML-screened candidates contain triple-point mutations, and the major residues are S100, Y102, S103, A104, and D105 (Supplemental Table 10). The minor mutation residues of these candidates are W106, E108, D109, and Y110, whereas there is no mutation at T97, T98, L99, G101, or P107, as shown in Supplemental Table 10.

**Figure 5:**
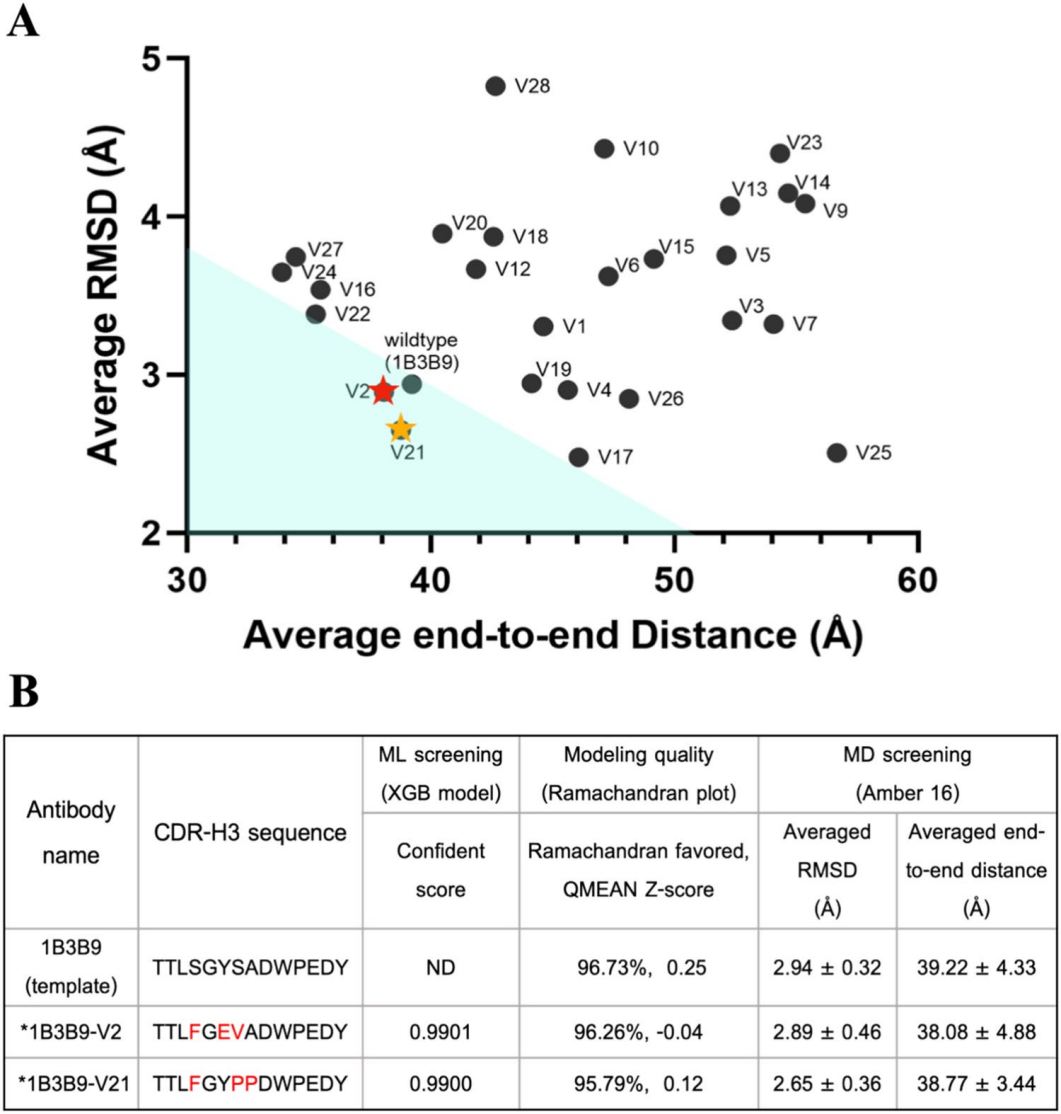
ML and MD screening for antibody candidates. We generated approximately 2.5 million CDR-H3 variants from human neutralizing 1B3B9 antibody (template) by random mutation with single, double, and triple points for further screening by ML and MD methods. (**A**) Distribution of ML-screened NAb candidates by scatter plot of the average end-to-end distance (Å) and average RMSD (Å) calculated by Amber 16. The 1B3B9 (template) antibody is indicated by a green circle. The final screened 1B3B9_V2 and 1B3B9_V21 NAbs are shown as red and yellow stars. (**B**) ML, structure modelling quality, and MD analysis of the antibody template (1B3B9) and outstanding candidates (1B3B9_V2 and 1B3B9_V21). Red letters represent mutated amino acid residues of outstanding antibody candidates compared with the antibody template.

To empower the screening framework and increase antibody developability, we screened these 28 ML-screened candidates with an MD simulation tool to test the stability of each antibody molecule. We designed a simple MD by performing homology modelling of these candidates, introducing the mutated CDR-H3 sequences into the 1B3B9 antibody template, and sent it to the SWISS-MODEL server. We determined the quality of each modelled structure with Ramachandran favoured and QMEAN scores before performing MD simulations. All constructed 3D structures showed QMEAN scores less than 1, reflecting the native-like structure (Supplemental Table 10). Then, we used MD to screen a stable conformation of each 3D structure and showed MD-screened candidates in a scatter plot with the average RMSD (a standard measure of structural distance between coordinates or structure changes; Y axis) and average end-to-end distance (a distance between the first and the last carbon atom in a protein or structure length; X axis) values, which are the representative parameters for molecular stability in Fig. 5A. From this MD plot, 1B3B9 (green circle), 1B3B9_V2 (red star), and 1B3B9_V21 (yellow star) had average values (X, Y) of (39.22 ± 4.33, 2.94 ± 0.32), (38.08 ± 4.88, 2.89 ± 0.46), and (38.77 ± 3.44, 2.65 ± 0.36), respectively (Fig. 5A,B). The RMSD plot of all structures is provided in Supplemental Fig. 1. We accordingly concluded that 1B3B9_V2 and 1B3B9_V21 have lower RMSD and end-to-end distance values than the 1B3B9 template and other candidates, which implies that the two candidates have more stable structural conformations and higher developability as synthetic antibodies. In this finding, we proposed the best 1B3B9_V21 antibody, which has the lowest average RMSD, representing a more stable configuration, for further characterization as a potent and stable candidate targeting the four envelope proteins of DENV-1 to DENV-4.

### In silico characterization of the best 1B3B9_V21 NAb candidate

**Figure 6:**
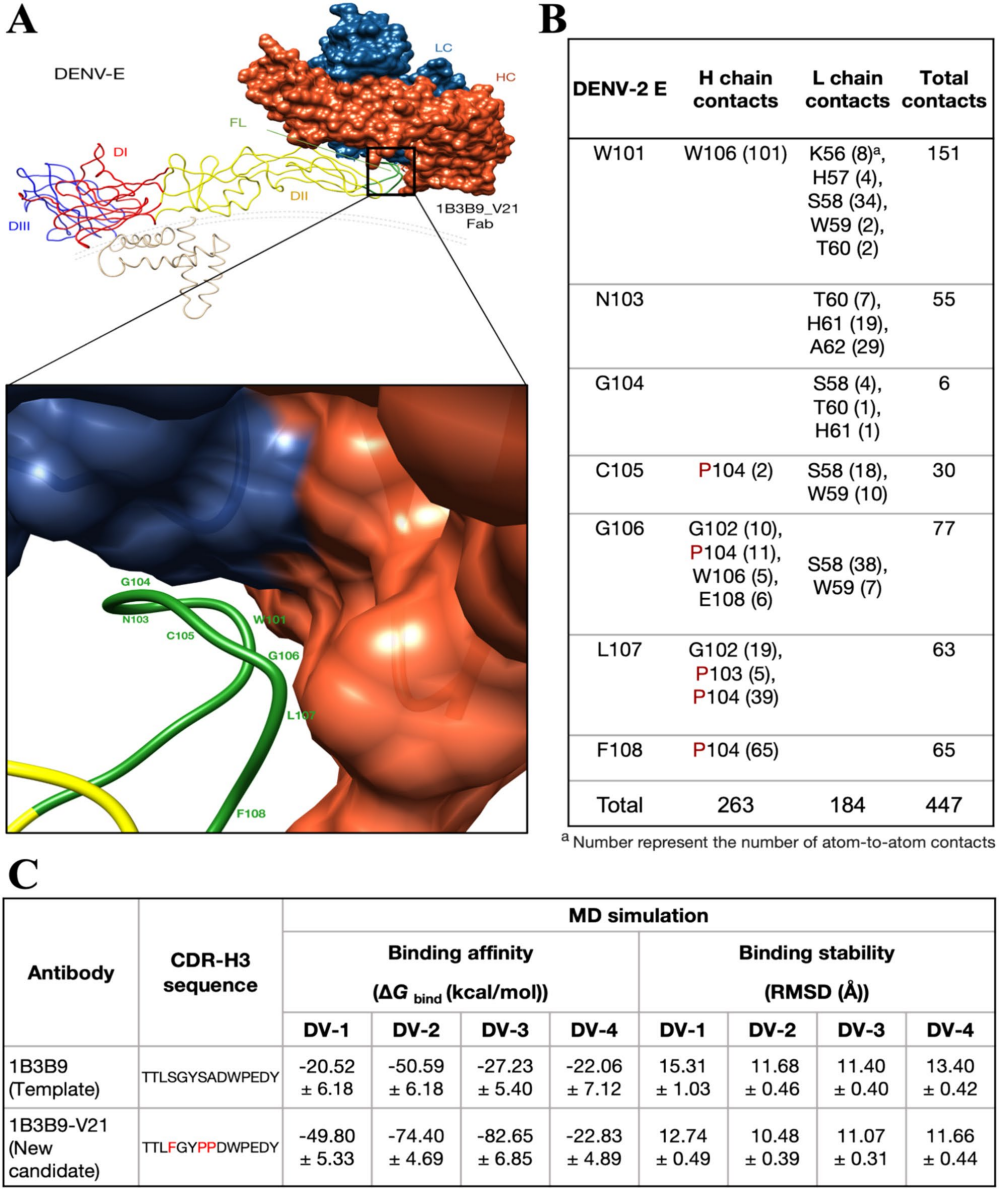
Molecular docking and MD analysis of the most potent and stable neutralizing antibody candidate (1B3B9_V21) against DENV-1 to DENV-4. We obtained the best NAb candidate, 1B3B9_V21, with potent and stable neutralizing activities against all four serotypes. We performed homology modelling, molecular docking, and MD simulations. (**A**) Structure of 1B3B9_V21 Fab bound to the fusion loop epitope on the EDII domain of DENV-2 (PDB ID: 5A1Z). (**B**) Residues participating in interactions between DENV-2 E and 1B3B9_V21 Fab are shown in the table with the number of atom-to-atom contacts analysed by the contact command of AMBER16. The distance cut-off is 4.5 Å. (**C**) Binding energies of 1B3B9_V21 with DENV-1 to DENV-4 (PDB ID: 4CCT, 5A1Z, 3J6T, 4CBF) were determined in terms of binding affinities (Δ*G*_bind_; kcal/mol) and binding stabilities (RMSD; Å). Red letters represent mutated amino acid residues of the new antibody candidate compared with the antibody template.

We investigated the ability of the best predicted antibody candidate (1B3B9_V21) compared to the real-world antibody template (1B3B9) in terms of binding interactions and binding energies (affinities and stabilities) using molecular docking and MD simulations, respectively. We used complexed four envelope proteins of DENV-1 to DENV-4 (PDB: 4CCT, 5A1Z, 3J6T, 4CBF) with 1B3B9 and 1B3B9_V21 in both docking and MD experiments. In a molecular docking study at a resolution of 4.5 Å, 1B3B9_V21 straddled FL epitopes from the top of EDII with interactions involving both the heavy chain and light chains (Fig. 6A). The binding motifs of 1B3B9_V21 were located at the fusion loop (W101, N103, G104, C105, G106, L107 and F108 residues) on the E protein of DENV-2 (Fig. 6A) with 447 atom-to-atom contacts (Fig. 6B). Structural analysis showed that 1B3B9 and 1B3B9_V21 bound to different regions of FL epitopes within R99—F108 (Supplemental Tables 11 and 12). 1B3B9_V21 revealed higher numbers of atom-to-atom contacts with DENV-2 E, DENV-3 E, and DENV-4 E proteins than 1B3B9, except DENV-1 E protein (Supplemental Tables 11 and 12). We therefore sought to determine whether 1B3B9_V21 could bind to DENV-1 to DENV-4 with strong affinity. Next, we tested the binding affinities and stabilities of 1B3B9 and 1B3B9_V21 with DENV-1 to DENV-4 by using MD simulations. We found that 1B3B9_V21 in complex with DENV-1, DENV-2 and DENV-3 showed lower Δ*G*_bind_ energies with lower average RMSD values (except for the DENV-3 system) than 1B3B9, whereas the Δ*G*_bind_ of 1B3B9_V21 in complex with DENV-4 was similar to that of 1B3B9 (Fig. 6C and Supplemental Fig. 2). These results were in good agreement with H-bond and contact analyses showing that 1B3B9_V21 in complex with DENV-1, DENV-2 and DENV-3 exhibited higher H-bond formations and number of contact atoms compared to 1B3B9_V21 in complex with DENV-4 (Supplemental Figs. 3 and 4). The RMSD plots of all antibody–antigen complexes are available in Supplemental Fig. 2. All data from in silico characterization indicated that 1B3B9_V21, which is the best NAb candidate from our computational framework, exhibits cross-neutralizing and stable binding with DENV-1 to DENV-4. Moreover, 1B3B9_V21 also provided binding interactions for these cross-reactive FL epitopes to other flaviviruses such as Zika virus and Japanese encephalitis virus (Supplemental Fig. 5). Binding site was also occupied between FL and 1B3B9_V21 heavy chain especially CDR-H3 mutated amino acid sequence (Supplemental Table 13). We suggest that our computational framework has potential for use in the efficient design and screening of potent and stable dengue therapeutic antibodies.

## Discussion

Antibody treatment is a promising strategy to combat severe dengue. An ideal antibody therapeutic should sufficiently neutralize all four DENV serotypes to reduce ADE effects, which is the major problem for dengue treatment^9,10^. A simple, rapid, and efficient antibody design and screening method, especially an in silico method, is the first crucial step to accelerate antibody discovery and the development of dengue therapeutic antibodies.

In this study, we propose a well-characterized dataset and in silico approach to predict potent and stable neutralizing antibodies against DENV-1 to DENV-4 to decrease ADE effects by screening for antibodies with strong and stable binding, thus providing an outstanding 1B3B9_V21 antibody candidate for further antibody engineering and optimization. We newly generated a CDR-H3-epitope-IC50 dataset from well-defined and real experimental results of 100 publications from 1992 to 2022. IC50 values obtained from different laboratory methods are unlikely to significantly impact algorithms, as each assay can accurately reflect the actual activities being measured^28^. We found that our dataset has a balance of neutralizing (positive) and nonneutralizing (negative) categories with highly diverse properties of CDR-H3 sequences and lower variation among epitope sequences, similar to the results of a previous study^23^. Our computational framework is designed to combine sequence-based ML screening and simple MD screening to identify the most potent and stable candidates in the most practical workflow. The combination of the XGB model and fingerprint-based method achieved excellent ML performance (AUC = 0.885 by independent test) in the prediction of cross-neutralizing antibodies against four DENV serotypes. We chose the AUC metric to evaluate the model's performance and selection for classifying neutralizing and non-neutralizing antibodies because of its comprehensive evaluation across all potential classification thresholds and various operational scenarios, as demonstrated in previous study^23^. Interestingly, the ML screener was used to screen approximately 2.5 million unseen antibodies to obtain 28 NAb candidates. The MD screener was used to screen 28 potential antibodies to find 2 stable NAb candidates, which may indicate the developability of these screened candidates as synthetic antibodies. Compared to existing approaches^17,18,23^, our framework uses a sequence-based ML model (no need for antibody and antigen structural data) and a simple MD protocol (requiring only an antibody structure with 15 ns MD production) that is easily applied in real-world experiments. In addition, our ML-based framework might be usable for antibody screening against other flaviviruses according to the cross-reactive epitopes that we used in this study, which are conserved among DENV, Zika, Japanese encephalitis, yellow fever, West Nile, and tick-borne encephalitis viruses^29,30^. Our approach has five key features. (1) It has two successive steps: general ML and simple MD methods. (2) It reduces the limitations of needing the full antibody sequence (instead requiring only the CDR-H3 sequence) and full antigen sequence. (3) It decreases time-consuming tasks by running all processes within 2 weeks. (4) It enables cost-effective screening to reduce laboratory work. (5) It is applicable to screening other flaviviruses because of the use of cross-reactive epitopes as shown in Supplemental Table 13.

Regarding the details of ML analysis, we considered the fingerprint-based method to be suitable for the prediction of antibody–antigen interactions because it can extract information from biophysical and biochemical properties without relying on the sequence or 3D structure. This approach facilitates the identification of unknown therapeutic candidates on a large scale for subsequent experimental validation^31^. We identified that the important features of 28 NAb candidates belong to the substructure count fingerprint (FP4C), which lists the top 30 fingerprints alongside their respective descriptions in Supplemental Table 9. Three of the top 30 fingerprints belong to the general class of alcohols (FP4C14: secondary alcohol, FP4C13: primary alcohol, and FP4C12: alcohol), which are found in the side chains of certain amino acids such as serine and threonine. These hydroxyl groups can act as both hydrogen bond donors and acceptors, influencing hydrogen bonding networks, binding affinity, and the neutralizing activity of antibodies^32^. Two of the 30 most important features were FP4C274 (aromatic ring) and FP4C184 (heteroaromatic ring), which are present in the side chains of certain amino acids like phenylalanine, tryptophan, and histidine. Aromaticity plays a significant role in cooperative interactions involving hydrophobicity, charge, and hydrogen bonding properties, making it well-suited for creating binding sites for epitopes, irrespective of the presence of polar and nonpolar surface residues^33^. For the FP4C307 (chiral center) fingerprint, it is recognized as a pivotal feature that significantly improves the predictive performance of machine learning models in drug discovery tasks, particularly in predicting KRAS G12C inhibitors and other biological activities^34^. Regarding FP4C84 (carboxylic acid) and FP4C88 (carboxylic acid derivative), which are found in the side chains of amino acids like aspartic acid and glutamic acid, they participate in hydrogen bonding, ionic interactions, and salt bridges critical for protein structure and protein–protein interactions^35^. FP4C302 (rotatable bond), with an average F-score of 40, represents single bonds that allow free rotation, contributing to molecular flexibility. This structural characteristic is crucial for determining overall molecular flexibility. Interestingly, all compounds in the dataset possessed rotatable bonds, particularly abundant in active compounds, suggesting their importance for biological activity^36^. FP4C295 (C–O–N–S bond) refers to the presence of a carbon–oxygen–sulfur bond in molecular structures. This feature can influence protein–protein binding by participating in hydrogen bonding interactions with amino acid residues on the protein surface, enhancing binding affinity and specificity^37^. Additionally, other significant FP4Cs include miscellaneous descriptors such as FP4C1-3 and FP4C300. FP4C1 (primary carbon), FP4C2 (secondary carbon), and FP4C3 (tertiary carbon) carbons indicate that carbon atoms with two or three neighbouring carbons in drug molecules may enhance metabolic stability by limiting access to metabolic pathways^38^. Based on feature importance (Supplemental Table 9) and amino acid analysis (Fig. 4), we suggest that the essential characteristics of our identified neutralizing antibodies encompass alcohols (present in amino acids S, T, and Y) and aromaticity (linked to amino acids F, Y, and W). These features are essential for the binding mechanism and maintaining the correct secondary structure at the antibody–antigen binding sites^39^.

Furthermore, we observed that the mutated residues of outstanding candidates are mostly located in the central area of the CDR-H3 region (S100, Y102, S103, A104, D105, W106, E108, D109, and Y110), which are crucial sites for antibody engineering and improvement, as supported by previous studies^40,41^. After characterization by in silico methods, 1B3B9_V21 revealed binding motifs at the most cross-reactive fusion loop region on the EDII protein of DENV-1 to DENV-4 (W101, G102, G104, G106, and L107 residues). These binding sites have been reported in many studies of cross-neutralizing antibodies against all four DENV^6,29,42^. In molecular dynamics, 1B3B9_V21 showed high binding affinities (less than -22.83 ± 4.89 kcal/mol) and stabilities (less than 11.83 ± 1.960 Å) with DENV-1 to DENV-4. Most of the binding energies of 1B3B9_V21 are lower than those of the real 1B3B9 antibody. Therefore, we suggest 1B3B9_V21 as a potent and stable NAb candidate for development as a therapeutic agent. To accomplish the application of the NAb candidates, in vitro tests of the neutralizing and ADE activities of these predicted antibodies are still required.

In conclusion, we have presented the first ML-based framework that employed a sequence-based ML method and a simple MD method for the accurate and rapid identification of NAbs against DENV-1 to DENV-4. We also provided an updated dengue antibody dataset including unique information on CDR-H3 sequences, epitope sequences, and IC50 values. We used three different feature-encoding methods and ten ML algorithms to compare and exhibit the best performing model. Our ML model can be used for large-scale identification of NAbs. The MD method can be used to select stable NAbs for more accurate identification. Our outstanding 1B3B9_V21 candidate showed high potential for development as a therapeutic antibody, and this NAb candidate is warranted for further in vitro analysis. Our proposed computational framework might support novel opportunities to discover, design, and engineer therapeutic antibodies against four dengue viruses and other flaviviruses.

## Methods

### Computational framework design

We built a dataset, model, and computational framework for screening cross-neutralizing antibodies against DENV. The computational framework contains four steps (Fig. 1): (1) dataset preparation of CDR-H3-epitope-IC50 pairing data, (2) feature extraction and ML analysis for the best performing model, (3) generation of CDR-H3 antibody variants and ML screening for potential antibody candidates, and (4) MD screening for stable antibody candidates.

### Dataset preparation

The dataset of anti-DENV CDR-H3 sequences was prepared from published data that satisfied three criteria: (1) including complete CDR-H3 amino acid sequences specific to DENV and published in PubMed or Google patent databases, (2) including epitopes on the E protein in EDII (fusion loop), EDI-EDII (interdomain), EDIII, or EDE, and (3) including neutralizing activities against DENV in µg/ml, as characterized by a focus reduction neutralization test (FRNT) or plaque reduction neutralization test (PRNT) or enzyme-linked immunosorbent assay (ELISA). The CDR-H3 sequences were annotated using the IMGT numbering scheme through the Antibody Region-Specific Alignment (AbRSA) web service. The neutralization (NT) activities derived from FRNT and PRNT represent actual experimental NT values, while antibodies that do not bind in the ELISA assay were categorized as non-neutralizing because they fail to bind to the antigen. All data (Supplemental Table 1) were carefully preprocessed to handle missing data and remove redundant data. The compilation of interactions between CDR-H3 and epitope sequences followed the principles outlined by Magar et al*.*^23^. Our dataset was derived from experimental results that included the sequences of CDR-H3 and epitopes, along with corresponding in vitro IC50 values against any of the four serotypes of dengue virus. In this dataset, we collected CDR-H3 sequences along with their epitopes and neutralizing activities (IC50; µg/ml) and further divided them into a positive (neutralizing) dataset (IC50 ≤ 10 µg/ml) and a negative (nonneutralizing) dataset (IC50 > 10 µg/ml). Each positive and negative dataset was randomly divided into a training dataset (80%) and an independent dataset (20%) (Table 1).

### Feature extraction and machine learning analysis

To evaluate the contribution of variant ML methods at the different levels of each antibody–antigen interaction, we performed a comparative analysis on different feature extraction schemes and ML methods. In the case of the feature extraction schemes, we applied three types of feature descriptors addressing multiple aspects, including 12 sequence-based, 7 atom-based, and 12 fingerprint-based feature descriptors (Supplemental Tables 2–4) following the previous studies^43–45^. The CDR-H3 and epitope sequences were concatenated and encoded into a single embedding of an antibody–antigen complex using our in-house Python-based code. Data normalization was performed to transform the input features into the same scale using Min–Max Scaler. Afterwards, the scaled training set was employed to train ten different ML algorithms: extreme gradient boosting (XGB), support vector machine (SVM), random forest (RF), partial least squares regression (PLS), multilayer perceptron (MLP), logistic regression (LR), linear support vector classification (LN), extra tree (ET), decision tree (DT), and k-nearest neighbours (KNN) classifiers. The algorithm selection was guided by considerations of accuracy, computational resources, and interpretability to derive the optimal predictive model for the dataset. Simpler models such as PLS, LR, LN, and DT provide easier interpretability, whereas more complex models like XGB, SVM, RF, MLP, ET, and KNN offer greater effectiveness in capturing intricate patterns within high-dimensional data spaces and deliver superior predictive capabilities^24^. All models are designed specifically for binary classification. Hyperparameter searching was performed as described in Supplemental Table 5. Herein, we evaluated and compared the performance of the developed ML methods based on tenfold cross-validation (CV) and independent tests. We demonstrated ML performances based on five metrics: accuracy (ACC), sensitivity (Sn), specificity (Sp), Matthew’s correlation coefficient (MCC), and area under the ROC curve (AUC), as previously described^43,46^. The ML algorithm with the greatest AUC for predicting NAbs was selected to screen a panel of unseen antibodies in the ML screening step. Model comparison, analysis and evaluation were performed using Python script 3.8 (all codes; available at GitHub).

### In silico antibody library

An in silico antibody library of 2,529,794 variants was generated by making single, double, and triple mutations on all amino acids in the CDR-H3 region of the 1B3B9 neutralizing human monoclonal antibody (antibody template)^6^ using Python script 3.8. All antibody variants were screened and ranked by the best ML algorithm and simple MD simulation in further steps to discover new potent and stable NAb candidates.

### ML and MD screening methods

In the first ML screening step, all antibody variants were filtered by the best performing ML algorithm with a confidence score cut-off of 0.990 to screen for potent neutralizing antibody candidates against four serotypes of DENV. Then, we constructed the 3D structure of the 1B3B9 Fab antibody template and ML-screened antibody variants using the SWISS-MODEL server^47^ (PDB 3t2n.1). Homology assessments and structural comparisons were performed using the SWISS-ExPASy server in terms of Ramachandran plots and QMEAN scores^48^ to validate the model quality of all 3D structures before screening by MD simulation.

In the second MD screening step, to determine the Fab structural stability as previously described (18), we performed MD simulations of all Fab antibody variants in a solvated environment using Amber 16. Each system was simulated under the periodic boundary condition with isothermal-isobaric (NPT) ensemble. The temperature and pressure were controlled by using Langevin thermostat^49^ with collision frequency of 2.0 and Berendsen barostat^50^. Topologies of each Fab were generated according to the ff14SB forcefield^51^. The Fab molecule was cantered in a cubic simulation box, extending 1 nm from the molecule surface. Then, the system box was solved by TIP3B model water atoms^52^, and a net positive charge was neutralized with chloride ions. Energy minimization was carried out using 1500 steps of steepest descent (SD) followed by 1500 steps of conjugated gradient (CG) methods with constrained solvent molecules. Then, the whole system was fully minimized using the same procedure. The minimized system was subjected to two rounds of equilibration at 310 K and 1 atm. First, the molecular system was equilibrated in the NVT ensemble for 100 picoseconds and a 2-femtosecond time step. Second, the equilibration was applied in a round of NPT simulation for 100 picoseconds to ensures that the simulated system is at physiological temperature and pressure. Then, the system was carried out in NPT and no constraints for 500 picoseconds. Finally, a 15 ns unrestrained NPT simulation at 310 K and 1 atm was carried out under identical simulation parameters. We collected the MD results and demonstrated the stabilities of all variants in the scatter plot of average end-to-end distance values at the X axis and averaged the root-mean-square displacement (RMSD) values at the Y axis to select the outstanding NAb candidates.

### Characterization by molecular docking and MD simulation

The antibody template (1B3B9) and the best screened NAb candidate were further characterized for their binding sites, binding affinities, and binding stabilities with DENV-1 to DENV-4 (utilizing PDB IDs: 4CCT, 5A1Z, 3J6T, 4CBF, which correspond to the strains used in previous in vitro studies)^6^ using molecular docking and MD simulation. First, antibody–antigen docking was performed by using the ZDOCK server^53^ using the default parameters and the target residues. The best posture of each docked complex was selected according to the lowest-energy ZRANK score. Then, MD simulations of each antibody–antigen complex were executed at 310 K and 1 atm for 100 ns using Amber 16. The MD procedure was the same as in the MD screening method above. The MD trajectories were saved every 10 ps.

The binding affinity of the antibody–antigen complex was calculated as binding free energy (Δ*G*_bind_; kcal/mol) based on the MM/PB(GB)SA approach from the last 20 ns of the MD production using the CPPTRAJ^54^ and MMPBSA.py^55^ modules of AMBER16. The binding stability of each complex was evaluated in terms of RMSD according to the number of antigen–antibody hydrogen bonds and the number of atomic contacts using the CPPTRAJ modules of AMBER16. The H-bond interactions were calculated using two criteria: (1) distance between the hydrogen donor (HD) and hydrogen acceptor (HA) of ≤ 3.5 Å and (2) HD–H···HA angle of ≥ 150°. The number of atom contacts was counted as the number of atoms within 4.5 Å of each complex.

### Supplementary Information

Supplementary Information.

## Data Availability

All data generated or analyzed during this study are included in this published article and its supplementary information files. The dataset, feature descriptions, ML performances, RMSD plots of MD simulations, data of ML-screened antibodies, and numbers of atom-to-atom contacts are provided in the supplementary information. All codes in this study are available online at https://github.com/PiyatidaNatsrita/In-silico-dengue-virus-antibody-prediction-2022.git.
