# Supplementary material for: Machine-learning-assisted high-throughput identification of potent and stable neutralizing antibodies against all four dengue virus serotypes
Source: Sci Rep. 2024 Jul 26;14:17165. doi: 10.1038/s41598-024-67487-8 (PMC11282219; doi:10.1038/s41598-024-67487-8)
Supplement: Supplementary file 1 — Supplementary Information. [file 41598_2024_67487_MOESM1_ESM.docx]

**Supplementary Information**

**Machine-learning-assisted high-throughput identification of potent and stable neutralizing antibodies against all four dengue virus serotypes**

Piyatida Natsrita^1,2^, Phasit Charoenkwan^3^, Watshara Shoombuatong^4^, Panupong Mahalapbutr^5^, Kiatichai Faksri^1,2^, Sorujsiri Chareonsudjai^1^, Thanyada Rungrotmongkol^6^, Chonlatip Pipattanaboon^1, 2^*

**Affiliations**

^1^Department of Microbiology, Faculty of Medicine, Khon Kaen University, Khon Kaen, 40002, Thailand.

^2^Research and Diagnostic Center for Emerging Infectious Diseases, Khon Kaen University, Khon Kaen, 40002, Thailand.

^3^Modern Management and Information Technology, College of Arts, Media and Technology, Chiang Mai University, Chiang Mai, 50200, Thailand.

^4^Center for Research Innovation and Biomedical Informatics, Faculty of Medical Technology, Mahidol University, Bangkok, 10700, Thailand.

^5^Department of Biochemistry, Faculty of Medicine, Khon Kaen University, Khon Kaen, 40002, Thailand.

^6^Center of Excellent in Biocatalyst and sustainable Biotechnology, Department of Biochemistry Faculty of Science, Chulalongkorn University, Bangkok, 10330, Thailand.

***Corresponding author**

Chonlatip Pipattanaboon, Department of Microbiology Faculty of Medicine, Khon Kaen University, Khon Kaen, 40002, Thailand.

E-mail: [chonpi@kku.ac.th](mailto:chonpi@kku.ac.th)

**Supplemental Figure 1: RMSD plot of 1B3B9 (template) antibody and 28 NAb variants.**


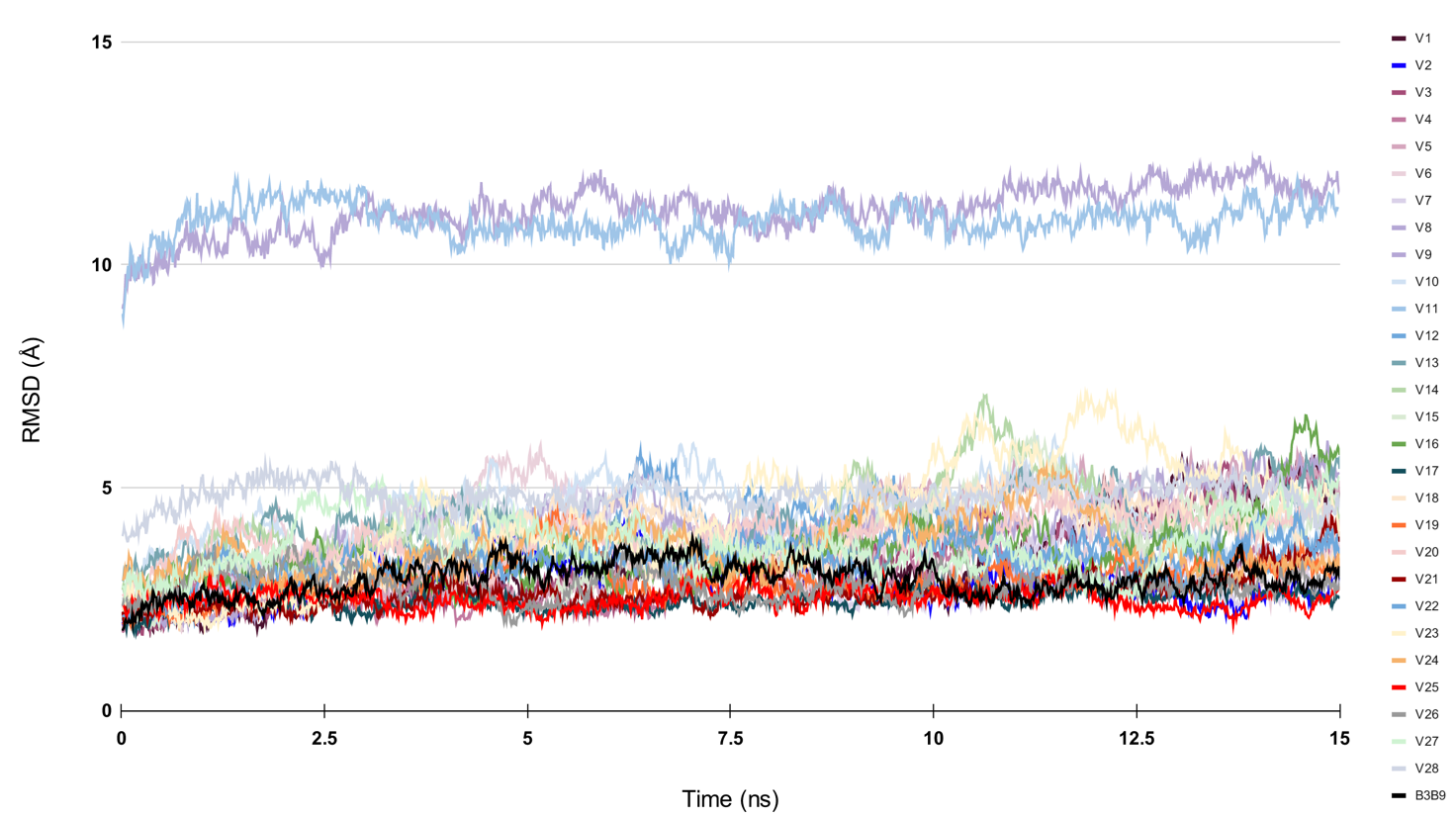


**Supplemental Figure 2: RMSD plot of 1B3B9 and 1B3B9_V21 NAbs with envelope protein of DENV-1 to DENV-4.**

**
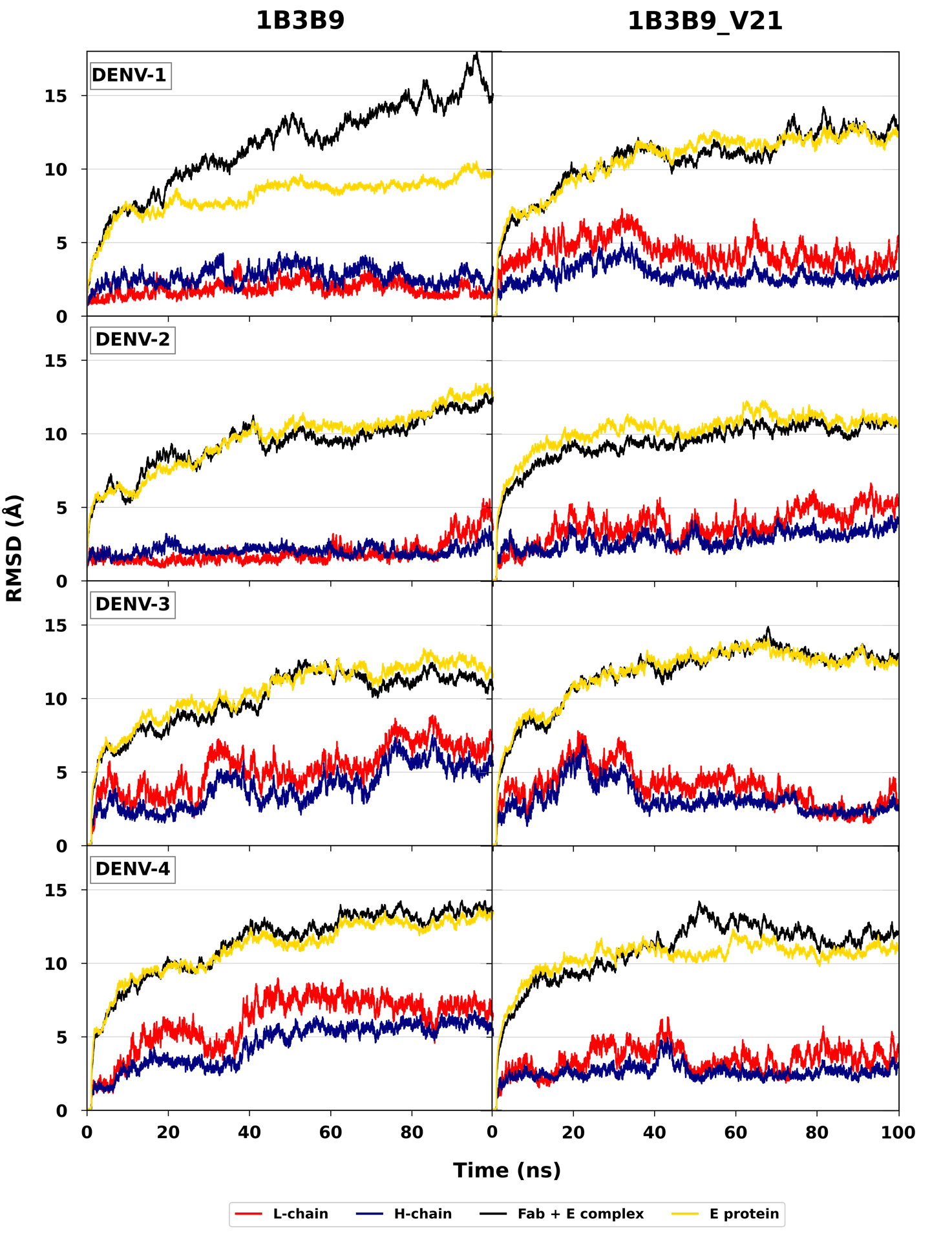
**

**Supplemental Figure 3: Number of hydrogen bond through time (100 ns) of 1B3B9 and 1B3B9_V21 NAbs with envelope protein of DENV-1 to DENV-4.**

**
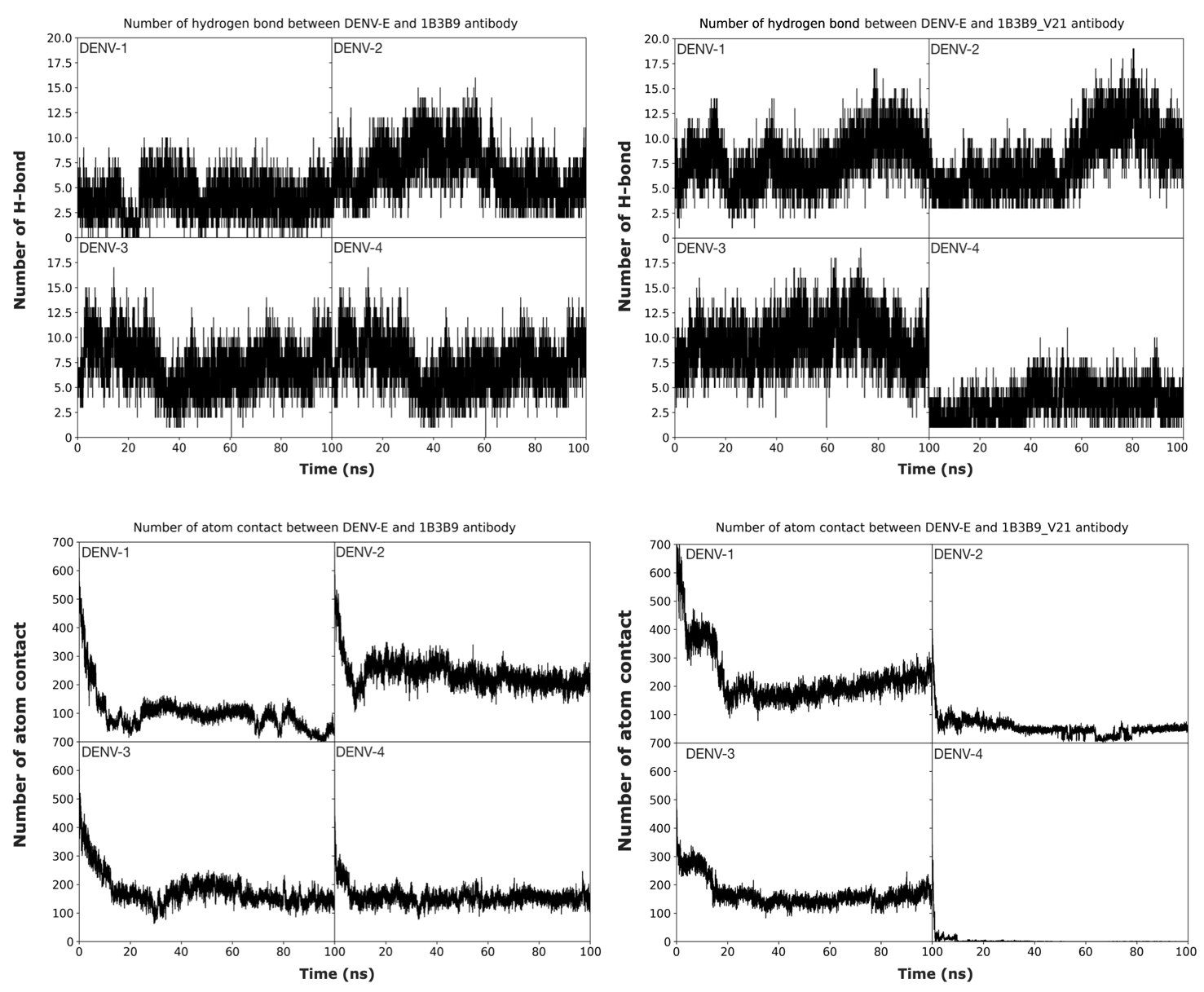
**

**Supplemental Figure 4: Number of atom contact through time (100 ns) of 1B3B9 and 1B3B9_V21 NAbs with envelope protein of DENV-1 to DENV-4.**

**
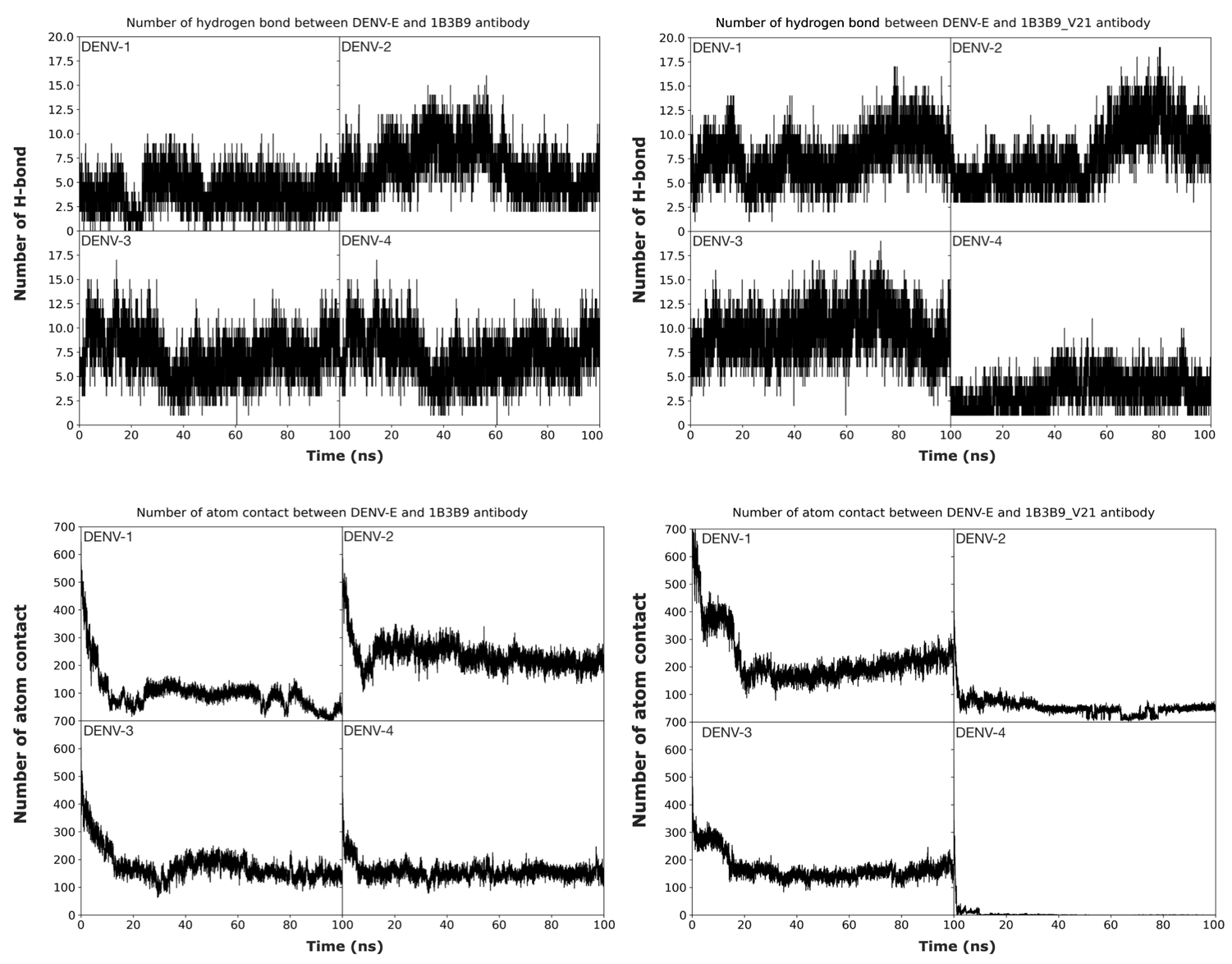
**

**Supplemental Figure 5: Molecular docking of 1B3B9_V21 antibody against envelope protein of Zika virus (ZIKV) and Japanese encephalitis virus (JEV)**

**
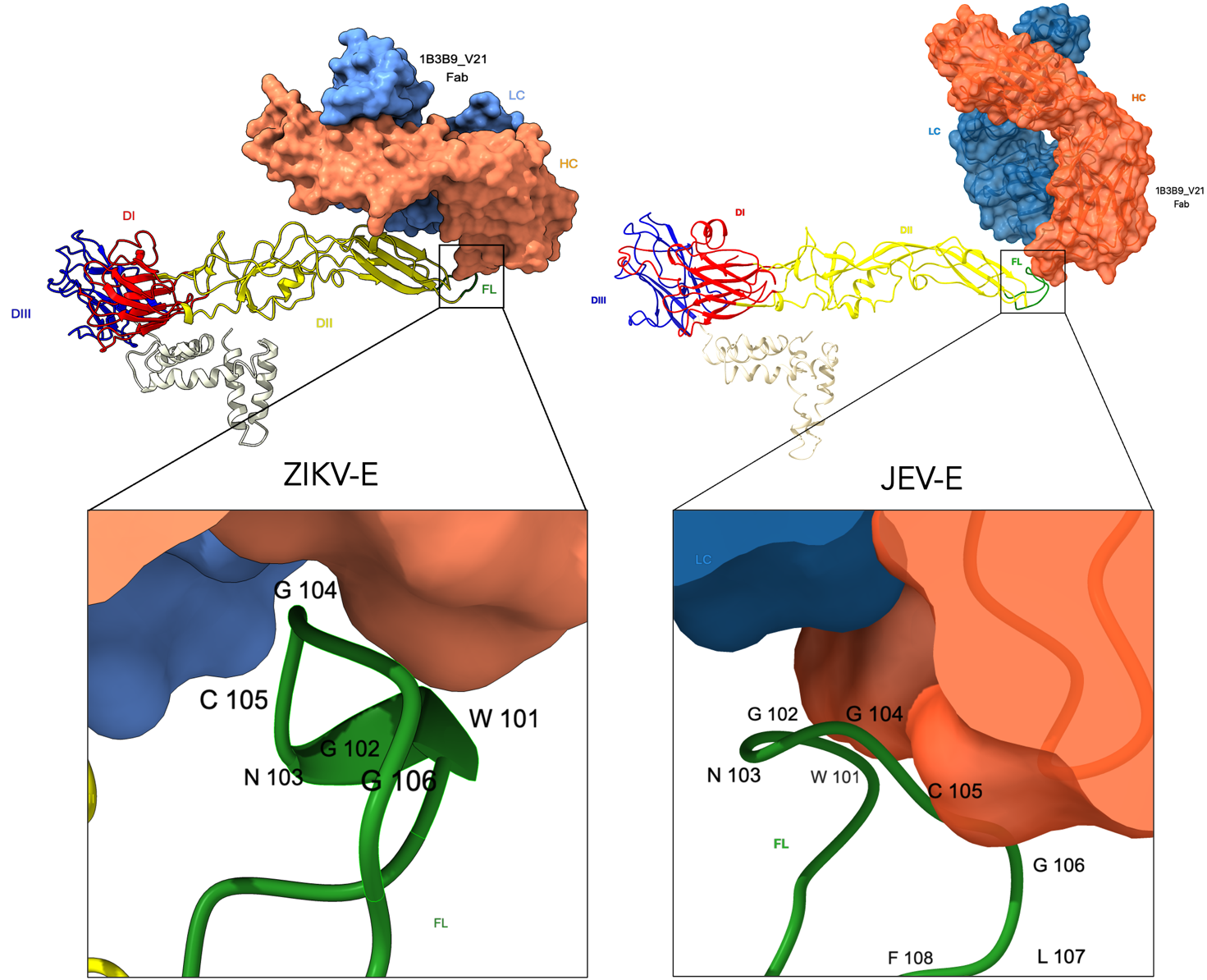
**

**Supplemental Table 1: Dataset used in this study (n = 1,108 interactions from 100 publications).**

| **Data** | **Antibody** | | **Epitope** | | | **Neutralizing activity** | | **Interaction label** | **Reference** |
| --- | --- | --- | --- | --- | --- | --- | --- | --- | --- |
|  | **Name** | **Sequence** | **DENV serotype** | **Epitope region** | **Sequence** | **Assay** | **IC50 (μg/mL)** |  | **(PMID or Patent number)** |
| **1** | 3E1 | VSLGDAWLLYWTP | DENV-3 | EDII | WF | NT50 | 0.44 | Neutralizing | 34155267 |
| **2** | 1E5 | ARGRPYIGGDYEYDYFYGMDV | DENV-3 | EDII | WLF | NT50 | 0.34 | Neutralizing | 34155267 |
| **3** | 4E11 | SRGWEGFAY | DENV-4 | EDIII | SIDKEMLLW | PRNT50 | 2.40 | Neutralizing | 26430770 |
| **4** | 4E11 | SRGWEGFAY | DENV-2 | EDIII | KFKVVKEIRQEDEKDSPGLKLNW | FRNT50 | 0.03 | Neutralizing | 23569282 |
| **5** | 4E11 | SRGWEGFAY | DENV-1 | EDIII | SFKLEKEVQKETDKEKPKLKLSW | FRNT50 | 0.06 | Neutralizing | 23569282 |
| **6** | 4E11 | SRGWEGFAY | DENV-3 | EDIII | TFVLKKEVKEKEKKEEPNLKINW | FRNT50 | 0.52 | Neutralizing | 23569282 |
| **7** | 4E11 | SRGWEGFAY | DENV-3 | EDIII | TFVLKKEVEKKKLKINW | FCNT50 | 8.00 | Neutralizing | 22285214 |
| **8** | 4E11 | SRGWEGFAY | DENV-1 | EDIII | SFKLEKEVQKEEKPLKLSW | FCNT50 | 0.16 | Neutralizing | 22285214 |
| **9** | 4E11 | SRGWEGFAY | DENV-2 | EDIII | KFKIVKEIREEKDPGLKLNW | FCNT50 | 0.13 | Neutralizing | 22285214 |
| **10** | 3E31 | SRGSKGAMDY | DENV-1 | EDIII | AETQHTLQITEE | PRNT50 | 1.80 | Neutralizing | 29249606 |
| **11** | 3E31 | SRGSKGAMDY | DENV-1 | EDIII | EKEVAETQHGTV | ELISPOT-MNT | 5.60 | Neutralizing | 23851440 |
| **12** | 3E31 | SRGSKGAMDY | DENV-3 | EDIII | KKEVSETQHGTI | ELISPOT-MNT | 0.40 | Neutralizing | 23851440 |
| **13** | 3E31 | SRGSKGAMDY | DENV-2 | EDIII | VKEIAETQHGTI | ELISPOT-MNT | 2.50 | Neutralizing | 23851440 |
| **14** | 14C10 | GIAGGWAFW | DENV-1 | EDE | TNQYLKGTTPQEILGTLKEK | PRNT50,BHK21 | 0.47 | Neutralizing | 27974667 |
| **15** | 14C10 | GIAGGWAFW | DENV-3 | EDE | TNQYLKGTTPQEILTKLKDN | FCNT50,Vero | 0.10 | Neutralizing | 30895307 |
| **16** | 14C10 | GIAGGWAFW | DENV-1 | EDE | TNQYLKGVTPQEVLGTLREK | FRNT50 | 3.16 | Neutralizing | 28251184 |
| **17** | 1A10 | ATYYADGSSYSEY | DENV-1 | Interdomain | GLH | PRNT50 | 0.94 | Neutralizing | 15542643, WO2005056600 |
| **18** | 1A10H7 | ARSRYYYDSDASNYGMDV | DENV-2 | EDII | CCWCCCK | VN50 | 2.20 | Neutralizing | 23545366, WO2013035345, WO2014064943A1 |
| **19** | 1A10H7 | ARSRYYYDSDASNYGMDV | DENV-3 | EDII | CCWCCCL | VN50 | 5.70 | Neutralizing | 23545366, WO2013035345, WO2014064943A1 |
| **20** | 1A10H7 | ARSRYYYDSDASNYGMDV | DENV-4 | EDII | CCWCCCS | VN50 | 4.60 | Neutralizing | 23545366, WO2013035345, WO2014064943A1 |
| **21** | 1A10H7 | ARSRYYYDSDASNYGMDV | DENV-1 | EDII | CCWCCCV | VN50 | 9.20 | Neutralizing | 23545366, WO2013035345, WO2014064943A1 |
| **22** | 1A1D | ARDYEGFAY | DENV-1 | EDIII | KFKLEKVKPKS | NT50 | 0.02 | Neutralizing | 26905804 |
| **23** | 1A1D | ARDYEGFAY | DENV-3 | EDIII | KFKVVKIQPKN | NT50 | 0.04 | Neutralizing | 26905804 |
| **24** | 1A1D | ARDYEGFAY | DENV-2 | EDIII | SFKVVKIQPKN | NT50 | 0.02 | Neutralizing | 26905804 |
| **25** | 1A1D-2 | ARDYEGFAY | DENV-3 | Interdomain | GLKEP | FRNT50,Vero | 1.26 | Neutralizing | 23162552 |
| **26** | 1A1D-2 | ARDYEGFAY | DENV-2 | EDIII | GKFKVVKIRQP | FCNT50 | 10.00 | Neutralizing | 22278250 |
| **27** | 1A1D-2 | ARDYEGFAY | DENV-2 | EDIII | GKFKVVKIRQPKLN | FCNT50 | 10.00 | Neutralizing | 22278250 |
| **28** | 1A1D-2 | ARDYEGFAY | DENV-1 | EDIII | GKV | PRNT50 | 1.68 | Neutralizing | 20832836 |
| **29** | 1A1D-2 | ARDYEGFAY | DENV-2 | EDIII | KKI | PRNT50 | 6.67 | Neutralizing | 20832836 |
| **30** | 1A1D-2 | ARDYEGFAY | DENV-3 | EDIII | VKV | PRNT50 | 10.00 | Neutralizing | 20832836 |
| **31** | 1A1D-2 | ARDYEGFAY | DENV-2 | EDIII | GKKK | PRNT50,BHK21 | 0.30 | Neutralizing | 17881453 |
| **32** | 1A5 | AREYCTGDTCFAHFDY | DENV-3 | Interdomain | CGLH | PRNT50 | 0.89 | Neutralizing | 15542643, WO2005056600 |
| **33** | 1A5 | AREYCTGDTCFAHFDY | DENV-4 | Interdomain | CVLH | PRNT50 | 4.30 | Neutralizing | 15542644, WO2005056600 |
| **34** | 1B2 | ARAVTAGMPAAGTLDH | DENV-2 | Interdomain | GLH | PRNT50 | 3.13 | Neutralizing | 15542643, WO2005056600 |
| **35** | 1B3B9 | TTLSGYSADWPEDY | DENV-2 | EDII | CCWCCCK | VN50 | 2.60 | Neutralizing | 23545366, WO2013035345, WO2014064943A1 |
| **36** | 1B3B9 | TTLSGYSADWPEDY | DENV-2 | EDII | CCCDRWCFCCK | FRNT50 | 0.13 | Neutralizing | 23545366, WO2013035345, WO2014064943A1 |
| **37** | 1B3B9 | TTLSGYSADWPEDY | DENV-3 | EDII | CCCDRWCFCCL | FRNT50 | 2.00 | Neutralizing | 23545366, WO2013035345, WO2014064943A1 |
| **38** | 1B3B9 | TTLSGYSADWPEDY | DENV-4 | EDII | CCCDRWCFCCS | FRNT50 | 2.00 | Neutralizing | 23545366, WO2013035345, WO2014064943A1 |
| **39** | 1B3B9 | TTLSGYSADWPEDY | DENV-1 | EDII | CCCDRWCFCCV | FRNT50 | 3.00 | Neutralizing | 23545366, WO2013035345, WO2014064943A1 |
| **40** | 1C19 | CAAGRRLTFAYW | DENV-3 | EDIII | RGE | ELISA | 0.04 | Neutralizing | 26962223 |
| **41** | 1C1G4 | ARGPDYESSDSPWFDY | DENV-2 | EDII | CCCDRWCFCCK | VN50 | 4.00 | Neutralizing | 23545366, WO2013035345, WO2014064943A1 |
| **42** | 1C1G4 | ARGPDYESSDSPWFDY | DENV-3 | EDII | CCCDRWCFCCL | VN50 | 9.20 | Neutralizing | 23545366, WO2013035345, WO2014064943A1 |
| **43** | 1C1G4 | ARGPDYESSDSPWFDY | DENV-4 | EDII | CCCDRWCFCCS | VN50 | 2.20 | Neutralizing | 23545366, WO2013035345, WO2014064943A1 |
| **44** | 1C1G4 | ARGPDYESSDSPWFDY | DENV-1 | EDII | CCCDRWCFCCV | VN50 | 10.00 | Neutralizing | 23545366, WO2013035345, WO2014064943A1 |
| **45** | 1C2D2 | ARVAKLFGSATYGMDV | DENV-2 | EDII | CCCDRWCFCCK | VN50 | 2.70 | Neutralizing | 23545366, WO2013035345, WO2014064943A1 |
| **46** | 1C2D2 | ARVAKLFGSATYGMDV | DENV-3 | EDII | CCCDRWCFCCL | VN50 | 4.20 | Neutralizing | 23545366, WO2013035345, WO2014064943A1 |
| **47** | 1C2D2 | ARVAKLFGSATYGMDV | DENV-4 | EDII | CCCDRWCFCCS | VN50 | 2.20 | Neutralizing | 23545366, WO2013035345, WO2014064943A1 |
| **48** | 1C2D2 | ARVAKLFGSATYGMDV | DENV-1 | EDII | CCCDRWCFCCV | VN50 | 5.60 | Neutralizing | 23545366, WO2013035345, WO2014064943A1 |
| **49** | 1C3 | ARDSGWYDF | DENV-3 | EDII | WL | NT50 | 0.38 | Neutralizing | 34155267 |
| **50** | 1E7B8 | ARHRAVAGGDSDHDENNWFGP | DENV-2 | EDII | CCCDRWCFCCK | FRNT50 | 1.50 | Neutralizing | 23545366, WO2013035345, WO2014064943A1 |
| **51** | 1E7B8 | ARHRAVAGGDSDHDENNWFGP | DENV-3 | EDII | CCCDRWCFCCL | VN50 | 2.80 | Neutralizing | 23545366, WO2013035345, WO2014064943A1 |
| **52** | 1E7B8 | ARHRAVAGGDSDHDENNWFGP | DENV-4 | EDII | CCCDRWCFCCS | VN50 | 1.90 | Neutralizing | 23545366, WO2013035345, WO2014064943A1 |
| **53** | 1F11 | AKDFLYYYDTNGDTGH | DENV-3 | EDII | WF | NT50 | 0.38 | Neutralizing | 34155267 |
| **54** | 1F4 | DKNPGTKPYYHYGMDV | DENV-1 | Interdomain | KNKETTTG | FCNT50,Vero | 0.11 | Neutralizing | 30185598 |
| **55** | 1F4 | DKNPGTKPYYHYGMDV | DENV-1 | Interdomain | LKETNKSATEVTAMTSVEVKPDSGTT | FCNT50,DC-SIGN | 10.00 | Neutralizing | 28251184 |
| **56** | 1F4 | DKNPGTKPYYHYGMDV | DENV-1 | Interdomain | LEG | FCNT50,DC-SIGN | 3.00 | Neutralizing | 22499787 |
| **57** | 1F4 | DKNPGTKPYYHYGMDV | DENV-1 | Interdomain | LKE | FCNT50,DC-SIGN | 3.00 | Neutralizing | 22499787 |
| **58** | 1F4 | DKNPGTKPYYHYGMDV | DENV-1 | Interdomain | LKG | FCNT50,DC-SIGN | 0.18 | Neutralizing | 22499787 |
| **59** | 1F4 | DKNPGTKPYYHYGMDV | DENV-1 | Interdomain | LKETNKSTETIATTTSEIQTDSGTT | PRNT50 | 0.03 | Neutralizing | 24421336 |
| **60** | 1F4 | DKNPGTKPYYHYGMDV | DENV-1 | Interdomain | LKETNKSSTETTATTTTEIQTDSGTT | PRNT50 | 0.05 | Neutralizing | 24421336 |
| **61** | 1G6 | DKNPGTKPYYHYGMDV | DENV-4 | EDIII | AGTLH | ELISA | 10.00 | Neutralizing | 26430770 |
| **62** | 1G6 | DKNPGTKPYYHYGMDV | DENV-4 | EDIII | ALGLH | ELISA | 10.00 | Neutralizing | 26430770 |
| **63** | 1G6 | DKNPGTKPYYHYGMDV | DENV-4 | EDIII | ALTGH | ELISA | 10.00 | Neutralizing | 26430770 |
| **64** | 1G6 | DKNPGTKPYYHYGMDV | DENV-4 | EDIII | ALTLG | ELISA | 10.00 | Neutralizing | 26430770 |
| **65** | 1G6 | DKNPGTKPYYHYGMDV | DENV-4 | EDIII | ALTLH | PRNT50 | 1.52 | Neutralizing | 26430770 |
| **66** | 1G6 | DKNPGTKPYYHYGMDV | DENV-4 | EDIII | GLTLH | ELISA | 10.00 | Neutralizing | 26430770 |
| **67** | 1G7C2 | ATLIAVAGSEGAGSFDI | DENV-2 | EDII | CCDRWCFCCK | FRNT50 | 1.10 | Neutralizing | 23545366, WO2013035345, WO2014064943A1 |
| **68** | 1G7C2 | ATLIAVAGSEGAGSFDI | DENV-3 | EDII | CCDRWCFCCL | FRNT50 | 4.50 | Neutralizing | 23545366, WO2013035345, WO2014064943A1 |
| **69** | 1G7C2 | ATLIAVAGSEGAGSFDI | DENV-4 | EDII | CCDRWCFCCS | VN50 | 2.20 | Neutralizing | 23545366, WO2013035345, WO2014064943A1 |
| **70** | 1G7C2 | ATLIAVAGSEGAGSFDI | DENV-1 | EDII | CCDRWCFCCV | VN50 | 9.90 | Neutralizing | 23545366, WO2013035345, WO2014064943A1 |
| **71** | 1H5A11 | ATGSQWPGDY | DENV-2 | EDII | CCCDRWCFCCK | VN50 | 1.50 | Neutralizing | 23545366, WO2013035345, WO2014064943A1 |
| **72** | 1H5A11 | ATGSQWPGDY | DENV-3 | EDII | CCCDRWCFCCL | VN50 | 5.30 | Neutralizing | 23545366, WO2013035345, WO2014064943A1 |
| **73** | 1H5A11 | ATGSQWPGDY | DENV-4 | EDII | CCCDRWCFCCS | VN50 | 1.20 | Neutralizing | 23545366, WO2013035345, WO2014064943A1 |
| **74** | 1H5A11 | ATGSQWPGDY | DENV-1 | EDII | CCCDRWCFCCV | VN50 | 5.20 | Neutralizing | 23545366, WO2013035345, WO2014064943A1 |
| **75** | 1M7 | CARFGPPYSQEEGVFHGAFDVW | DENV-3 | EDII | WG | FCNT50,Vero | 0.02 | Neutralizing | 24255124 |
| **76** | 1N5 | CAGEMATVFEYFQYW | DENV-3 | EDII | WLG | FCNT50,vero | 0.50 | Neutralizing | 24255124 |
| **77** | 2A10 | RDYYALDY | DENV-3 | EDII | DRW | FCNT50,U937-DC-SIGN | 5.84 | Neutralizing | 26905804 |
| **78** | 2A10 | RDYYALDY | DENV-2 | EDII | WGLFK | FCNT50,U937-DC-SIGN | 7.00 | Neutralizing | 26905804 |
| **79** | 2A10 | RDYYALDY | DENV-3 | EDII | WGLFL | FCNT50,U937-DC-SIGN | 5.00 | Neutralizing | 26905804 |
| **80** | 2A10 | RDYYALDY | DENV-4 | EDII | WGLFS | FCNT50,U937-DC-SIGN | 8.00 | Neutralizing | 26905804 |
| **81** | 2A10 | RDYYALDY | DENV-1 | EDII | WGLFV | FCNT50,U937-DC-SIGN | 7.00 | Neutralizing | 26905804 |
| **82** | 2A10G6 | GRRDYYALDY | DENV-1 | EDII | FVDRGWG | PRNT50,BHK21 | 1.82 | Neutralizing | 21264311 |
| **83** | 2A10G6 | GRRDYYALDY | DENV-2 | EDII | MVDRGWG | PRNT50,BHK21 | 1.23 | Neutralizing | 21264311 |
| **84** | 2A10G6 | GRRDYYALDY | DENV-4 | EDII | VVDRGWG | PRNT50,BHK21 | 1.31 | Neutralizing | 21264311 |
| **85** | 2A10G6 | GRRDYYALDY | DENV-3 | EDII | YVDRGWG | PRNT50,BHK21 | 2.19 | Neutralizing | 21264311 |
| **86** | 2A10G6 | GRRDYYALDY | DENV-2 | EDII | TQSWGCGLF | FACS | 10.00 | Neutralizing | 34267374 |
| **87** | 2A10G6 | GRRDYYALDY | DENV-1 | EDII | TQTWGCGLF | FACS | 10.00 | Neutralizing | 34267374 |
| **88** | 2A10G6 | GRRDYYALDY | DENV-3 | EDII | TQVWGCGLF | FACS | 10.00 | Neutralizing | 34267374 |
| **89** | 2A10G6 | GRRDYYALDY | DENV-4 | EDII | TQYWGCGLF | FACS | 10.00 | Neutralizing | 34267374 |
| **90** | 2C2 | ATLFVAAPGTGY | DENV-3 | EDII | WF | NT50 | 0.11 | Neutralizing | 34155267 |
| **91** | 2C8 | AKWDGHYFDY | DENV-2 | EDIII | MSYSMTGDGSPKKVEP | FRNT50 | 0.00 | Neutralizing | 30323338 |
| **92** | 2D22 | RPQSIFDWNFDL | DENV-4 | EDIII | KVQDDP | FRNT50 | 0.50 | Neutralizing | 29481552 |
| **93** | 2D22 | RPQSIFDWNFDL | DENV-4 | EDIII | KVKQGGDSP | FRNT50 | 1.00 | Neutralizing | 29481552 |
| **94** | 2D22 | RPQSIFDWNFDL | DENV-2 | EDIII | KVKQGKDSP | FRNT50 | 0.08 | Neutralizing | 29481552 |
| **95** | 2D22 | RPQSIFDWNFDL | DENV-2 | EDIII | KVKQEDSP | PRNT50 | 0.11 | Neutralizing | 26138979 |
| **96** | 2D22 | RPQSIFDWNFDL | DENV-2 | EDIII | KVKQGDSP | PRNT50 | 0.07 | Neutralizing | 26138979 |
| **97** | 2D22 | RPQSIFDWNFDL | DENV-2 | EDIII | KVKQGRDSP | FRNT50 | 0.15 | Neutralizing | 26463165 |
| **98** | 2D73 | RGYRYDGAHFDY | DENV-2 | EDIII | AETQHGTVITVNEE | ELISPOT-MNT | 0.30 | Neutralizing | 23851440 |
| **99** | 2D73 | RGYRYDGAHFDY | DENV-3 | EDIII | SETQHGTLITANEE | ELISPOT-MNT | 0.10 | Neutralizing | 23851440 |
| **100** | 2G2 | AILFNSDSPLDY | DENV-3 | EDII | WF | NT50 | 0.30 | Neutralizing | 34155267 |
| **101** | 2H12 | TRGGSHAMDY | DENV-1 | EDIII | KAETQHLIAE | FRNT50,Vero | 0.08 | Neutralizing | 22491255 |
| **102** | 2H12 | TRGGSHAMDY | DENV-3 | EDIII | KSETQHLIAE | FRNT50,Vero | 4.35 | Neutralizing | 22491255 |
| **103** | 2H5 | AREYCTGDTCFAHFDY | DENV-1 | Interdomain | GLH | PRNT50 | 0.47 | Neutralizing | 15542643, WO2005056600 |
| **104** | 2H5 | AREYCTGDTCFAHFDY | DENV-4 | Interdomain | GLQ | PRNT50 | 9.26 | Neutralizing | 15542643, WO2005056600 |
| **105** | 2H7 | AREYCTGGTCFAHFDY | DENV-1 | Interdomain | GLH | PRNT50 | 0.26 | Neutralizing | 15542643, WO2005056600 |
| **106** | 2H7 | AREYCTGGTCFAHFDY | DENV-4 | Interdomain | GLQ | PRNT50 | 7.26 | Neutralizing | 15542643, WO2005056600 |
| **107** | 2H7 | AREYCTGGTCFAHFDY | DENV-3 | Interdomain | VLH | PRNT50 | 5.92 | Neutralizing | 15542643, WO2005056600 |
| **108** | 2H8G1 | ATGGGRFSGSGNYYYYGMDV | DENV-2 | EDII | CCCDRWCFCCK | VN50 | 5.10 | Neutralizing | 23545366, WO2013035345, WO2014064943A1 |
| **109** | 2H8G1 | ATGGGRFSGSGNYYYYGMDV | DENV-4 | EDII | CCCDRWCFCCS | VN50 | 6.60 | Neutralizing | 23545366, WO2013035345, WO2014064943A1 |
| **110** | 3A10G12 | AAGSQWPGDY | DENV-2 | EDII | CCCDRWCFCCK | FRNT50 | 1.40 | Neutralizing | 23545366, WO2013035345, WO2014064943A1 |
| **111** | 3A10G12 | AAGSQWPGDY | DENV-3 | EDII | CCCDRWCFCCL | VN50 | 5.30 | Neutralizing | 23545366, WO2013035345, WO2014064943A1 |
| **112** | 3A10G12 | AAGSQWPGDY | DENV-4 | EDII | CCCDRWCFCCS | VN50 | 1.20 | Neutralizing | 23545366, WO2013035345, WO2014064943A1 |
| **113** | 3A10G12 | AAGSQWPGDY | DENV-1 | EDII | CCCDRWCFCCV | VN50 | 5.10 | Neutralizing | 23545366, WO2013035345, WO2014064943A1 |
| **114** | 3A1E2 | ARGMTGFTTSNTESFDL | DENV-2 | EDII | CCCDRWCFCCK | VN50 | 1.40 | Neutralizing | 23545366, WO2013035345, WO2014064943A1 |
| **115** | 3A1E2 | ARGMTGFTTSNTESFDL | DENV-3 | EDII | CCCDRWCFCCL | VN50 | 5.60 | Neutralizing | 23545366, WO2013035345, WO2014064943A1 |
| **116** | 3A1E2 | ARGMTGFTTSNTESFDL | DENV-4 | EDII | CCCDRWCFCCS | VN50 | 0.90 | Neutralizing | 23545366, WO2013035345, WO2014064943A1 |
| **117** | 3A1E2 | ARGMTGFTTSNTESFDL | DENV-1 | EDII | CCCDRWCFCCV | VN50 | 4.80 | Neutralizing | 23545366, WO2013035345, WO2014064943A1 |
| **118** | 3B6C7 | ASPGGLISDEAMAGYFDY | DENV-4 | EDII | CCRWCFCCS | VN50 | 3.60 | Neutralizing | 23545366, WO2013035345, WO2014064943A1 |
| **119** | 3B6C7 | ASPGGLISDEAMAGYFDY | DENV-2 | EDII | CCCDRWCFCCK | VN50 | 4.30 | Neutralizing | 23545366, WO2013035345, WO2014064943A1 |
| **120** | 3G9 | AKLFGVGDSDGY | DENV-3 | EDII | WF | NT50 | 0.62 | Neutralizing | 34155267 |
| **121** | 3H5 | ARKGGFAMDY | DENV-2 | EDIII | KEP | PRNT50,BHK21 | 0.13 | Neutralizing | 22509258 |
| **122** | 3H5 | ARKGGFAMDY | DENV-2 | EDIII | MTKEGDPFERKGVEPGQ | FRNT50 | 0.04 | Neutralizing | 30323338 |
| **123** | 3H5 | ARKGGFAMDY | DENV-2 | EDIII | APG | FRNT50 | 2.40 | Neutralizing | 25351518 |
| **124** | 3H5 | ARKGGFAMDY | DENV-2 | EDIII | KPG | FRNT50 | 0.20 | Neutralizing | 25351518 |
| **125** | 3H5 | ARKGGFAMDY | DENV-2 | EDII | MTKVP | FRNT50 | 0.20 | Neutralizing | 28631593 |
| **126** | 3H5 | ARKGGFAMDY | DENV-2 | EDII | QLKLNWFKKGSS | PRNT50 | 10.00 | Neutralizing | 1634111 |
| **127** | 3H5 | ARKGGFAMDY | DENV-2 | EDII | KLNWFKKGSSIGQ | ELISA | 10.00 | Neutralizing | 1634111 |
| **128** | 4A6F9 | ANTLWTVGSKGGFDY | DENV-2 | EDII | CCCDRWCFCCK | FRNT50 | 2.30 | Neutralizing | 23545366, WO2013035345, WO2014064943A1 |
| **129** | 4A6F9 | ANTLWTVGSKGGFDY | DENV-3 | EDII | CCCDRWCFCCL | VN50 | 5.50 | Neutralizing | 23545366, WO2013035345, WO2014064943A1 |
| **130** | 4A6F9 | ANTLWTVGSKGGFDY | DENV-4 | EDII | CCCDRWCFCCS | VN50 | 4.40 | Neutralizing | 23545366, WO2013035345, WO2014064943A1 |
| **131** | 4E5A | SRGWEGFAY | DENV-2 | EDIII | KFKIDKEIRQEGSDPNGLKLNW | FRNT50 | 0.03 | Neutralizing | 23569282 |
| **132** | 4E5A | SRGWEGFAY | DENV-4 | EDIII | KFSLDKEMKKEGANVNSLTLHW | FRNT50 | 4.00 | Neutralizing | 23569282 |
| **133** | 4E5A | SRGWEGFAY | DENV-1 | EDIII | SFKIDKEAQKEGAEPNKLKLSW | FRNT50 | 0.02 | Neutralizing | 23569282 |
| **134** | 4E5A | SRGWEGFAY | DENV-3 | EDIII | TFVIDKEVKEKGVEPNNLKINW | FRNT50 | 0.77 | Neutralizing | 23569282 |
| **135** | 4E5A-VH-Y106R | SRGWEGFAR | DENV-2 | EDIII | KFKIDKEIREGSDPNGLKLNW | FRNT50 | 0.03 | Neutralizing | 26189681 |
| **136** | 4E5A-VH-Y106R | SRGWEGFAR | DENV-4 | EDIII | KFSLDKEMKEGANVNSLTLHW | FRNT50 | 4.00 | Neutralizing | 26189681, WO2015122995 |
| **137** | 4E5A-VH-Y106R | SRGWEGFAR | DENV-1 | EDIII | SFKIDKEAQEGAEPNKLKLSW | FRNT50 | 0.02 | Neutralizing | 26189681, WO2015122995 |
| **138** | 4E5A-VH-Y106R | SRGWEGFAR | DENV-3 | EDIII | TFVIDKEVKKGVEPNNLKINW | FRNT50 | 0.77 | Neutralizing | 26189681, WO2015122995 |
| **139** | 4F5E1 | ARVTGGWSDY | DENV-2 | EDII | CCCDWCFCCK | FRNT50 | 4.60 | Neutralizing | 23545366, WO2013035345, WO2014064943A1 |
| **140** | 4F5E1 | ARVTGGWSDY | DENV-3 | EDII | CCCDRWCFCCL | VN50 | 9.70 | Neutralizing | 23545366, WO2013035345, WO2014064943A1 |
| **141** | 4F5E1 | ARVTGGWSDY | DENV-4 | EDII | CCCDRWCFCCS | VN50 | 4.70 | Neutralizing | 23545366, WO2013035345, WO2014064943A1 |
| **142** | 4G2 | ARIYHYDGYFDV | DENV-3 | Interdomain | GLKEEP | PRNT50 | 0.12 | Neutralizing | 29249606 |
| **143** | 4G2 | ARIYHYDGYFDV | DENV-1 | Interdomain | GLKEKP | PRNT50 | 0.02 | Neutralizing | 29249606 |
| **144** | 4G2 | ARIYHYDGYFDV | DENV-2 | Interdomain | GLKESP | PRNT50 | 0.04 | Neutralizing | 29249606 |
| **145** | 4G2 | ARIYHYDGYFDV | DENV-4 | Interdomain | GLKESV | PRNT50 | 0.07 | Neutralizing | 29249606 |
| **146** | 4G2 | ARIYHYDGYFDV | DENV-3 | Interdomain | WGGLFEP | PRNT50 | 2.40 | Neutralizing | 29425203 |
| **147** | 4G2 | ARIYHYDGYFDV | DENV-1 | Interdomain | WGGLFKP | PRNT50 | 0.56 | Neutralizing | 29425203 |
| **148** | 4G2 | ARIYHYDGYFDV | DENV-4 | Interdomain | WGGLFSV | PRNT50 | 2.30 | Neutralizing | 29425203 |
| **149** | 4G2 | ARIYHYDGYFDV | DENV-1 | Interdomain | GLKEP | FRNT50 | 10.00 | Neutralizing | 23162552 |
| **150** | 4G2 | ARIYHYDGYFDV | DENV-2 | Interdomain | GGLW | PRNT50,BHK21 | 10.00 | Neutralizing | 22278250 |
| **151** | 4G2 | ARIYHYDGYFDV | DENV-2 | Interdomain | LRWKP | PRNT50,Vero | 0.13 | Neutralizing | 22509258 |
| **152** | 4G2 | ARIYHYDGYFDV | DENV-4 | Interdomain | LRWSP | PRNT50,Vero | 0.13 | Neutralizing | 22509258 |
| **153** | 4G2 | ARIYHYDGYFDV | DENV-2 | EDII | GL | FRNT50 | 10.00 | Neutralizing | 22709350, 26265529 |
| **154** | 4G2 | ARIYHYDGYFDV | DENV-2 | EDII | GGL | PRNT50 | 5.50 | Neutralizing | 2578750 |
| **155** | 4G2 | ARIYHYDGYFDV | DENV-2 | EDII | WGLF | FC | 10.00 | Neutralizing | 26135599, US8637035B2 |
| **156** | 4H12C8 | TTLSGYSADWPEDY | DENV-2 | EDII | CCCNRWCFCCK | VN50 | 5.50 | Neutralizing | 23545366, WO2013035345, WO2014064943A1 |
| **157** | 5D9 | ARQGTGTTGVSEDPDLY | DENV-4 | EDI | KP | PRNT50 | 0.58 | Neutralizing | 15078949, WO2005056600 |
| **158** | 5E6B1 | TTLSGYSADWPEDY | DENV-2 | EDII | DRGWGNGCGLFGKGGIVTCAMFTCKKNMKGK | FRNT50 | 3.10 | Neutralizing | 24637211 |
| **159** | 5G2D2 | STYYYDGSDLTYGMDV | DENV-2 | EDII | CCCDRWCFCCK | VN50 | 3.90 | Neutralizing | 23545366, WO2013035345, WO2014064943A1 |
| **160** | 5G2D2 | STYYYDGSDLTYGMDV | DENV-3 | EDII | CCCDRWCFCCL | VN50 | 5.10 | Neutralizing | 23545366, WO2013035345, WO2014064943A1 |
| **161** | 5G2D2 | STYYYDGSDLTYGMDV | DENV-4 | EDII | CCCDRWCFCCS | VN50 | 2.30 | Neutralizing | 23545366, WO2013035345, WO2014064943A1 |
| **162** | 5G2D2 | STYYYDGSDLTYGMDV | DENV-1 | EDII | CCCDRWCFCCV | VN50 | 5.20 | Neutralizing | 23545366, WO2013035345, WO2014064943A1 |
| **163** | 5G8E3 | AVYYCARRGDYSSSAENFQH | DENV-2 | EDII | CCCDRWCFCCK | VN50 | 8.40 | Neutralizing | 23545366, WO2013035345, WO2014064943A1 |
| **164** | 5G8E3 | AVYYCARRGDYSSSAENFQH | DENV-4 | EDII | CCCDRWCFCCS | VN50 | 3.10 | Neutralizing | 23545366, WO2013035345, WO2014064943A1 |
| **165** | 5H2 | ARQGTGTTGVSEDSFDL | DENV-4 | EDI | VTATSSVEVKPDGEKR | PRNT50 | 0.24 | Neutralizing | 15078949, WO2005056600 |
| **166** | 5H2 | ARQGTGTTGVSEDSFDL | DENV-4 | EDI | AVTAMSSVEVKPDGEKR | FRNT50,C3/36 | 0.16 | Neutralizing | 28251184 |
| **167** | 5H2 | ARQGTGTTGVSEDSFDL | DENV-4 | EDI | VTAMSSVEVKPDGEKR | N50 | 10.00 | Neutralizing | 29215033 |
| **168** | 5H2 | ARQGTGTTGVSEDSFDL | DENV-4 | EDI | EP | NT50 | 10.00 | Neutralizing | 17881450 |
| **169** | 5H2 | ARQGTGTTGVSEDSFDL | DENV-4 | EDI | KP | NT50 | 0.30 | Neutralizing | 17881450 |
| **170** | 5J7 | ARDKELLFSRAFDI | DENV-3 | Interdomain | QLEKELA | FRNT50,BHK21 | 0.06 | Neutralizing | 26962223 |
| **171** | 5J7 | ARDKELLFSRAFDI | DENV-3 | Interdomain | ATQLATRCWGEKVQENQLTGSGFK | FCNT50,Vero | 0.10 | Neutralizing | 22499787 |
| **172** | 5J7 | ARDKELLFSRAFDI | DENV-3 | Interdomain | ATQLATRCWGEKVQENQMLGTVLK | FCNT50,Vero | 0.10 | Neutralizing | 22499787 |
| **173** | 5J7 | ARDKELLFSRAFDI | DENV-4 | Interdomain | ATQLATKRCWGEEKVQENQLTTKKDTPTIKKE | NT50 | 10.00 | Neutralizing | 29215033 |
| **174** | 5J7 | ARDKELLFSRAFDI | DENV-3 | Interdomain | ATQLATRCWGEKVQENQLTTIKKE | NT50,Vero | 0.01 | Neutralizing | 25698059 |
| **175** | 9F12 | TTSLY | DENV-4 | EDIII | KSKG | PRNT50 | 3.00 | Neutralizing | 26430770 |
| **176** | 9F12 | TTSLY | DENV-4 | EDIII | DKEMAETQHG | PRNT50,Vero | 0.30 | Neutralizing | 19264660 |
| **177** | 9F12 | TTSLY | DENV-1 | EDIII | EKEVAETQHG | PRNT50,Vero | 0.30 | Neutralizing | 19264660 |
| **178** | 9F12 | TTSLY | DENV-3 | EDIII | KKEVSETQHG | PRNT50,Vero | 0.30 | Neutralizing | 19264660 |
| **179** | 9F12 | TTSLY | DENV-2 | EDIII | VKEIAETQHG | PRNT50,Vero | 0.30 | Neutralizing | 19264660 |
| **180** | 9F12 | TTSLY | DENV-3 | EDIII | SYAMCLNTFKVL | PRNT50,Vero | 0.30 | Neutralizing | 19264660 |
| **181** | 9F12 | TTSLY | DENV-2 | EDIII | SYSMCTGKFKVV | PRNT50,Vero | 0.30 | Neutralizing | 19264660 |
| **182** | 9F12 | TTSLY | DENV-4 | EDIII | SYTMCSGKFKSI | PRNT50,Vero | 0.30 | Neutralizing | 19264660 |
| **183** | 9F12 | TTSLY | DENV-1 | EDIII | SYVMCTGSFKLE | PRNT50,Vero | 0.30 | Neutralizing | 19264660 |
| **184** | 9F12 | TTSLY | DENV-3 | EDIII | VEYKGEGSPCKI | PRNT50,Vero | 0.30 | Neutralizing | 19264660 |
| **185** | 9F12 | TTSLY | DENV-4 | EDIII | VKYEGAGSPCKI | PRNT50,Vero | 0.30 | Neutralizing | 19264660 |
| **186** | 9F12 | TTSLY | DENV-1 | EDIII | VKYEGTDAPCKI | PRNT50,Vero | 0.30 | Neutralizing | 19264660 |
| **187** | 9F12 | TTSLY | DENV-2 | EDIII | VQYEGDGSPCKI | PRNT50,Vero | 0.30 | Neutralizing | 19264660 |
| **188** | Ab513 | GWEGFAY | DENV-3 | EDIII | KFSIDKEMKKEGATNVNSLTLHW | PRNT50 | 0.10 | Neutralizing | 26189681, WO2015122995 |
| **189** | ADI-24191 | AKDRPLHGFGELYDH | DENV-1 | EDIII | TGSFKLKYEGTDGKEEKALK | FRNT50,Vero | 10.00 | Neutralizing | 28821561 |
| **190** | ADI-24192 | AKDRSTRGFGELLNY | DENV-3 | EDIII | LNTFVLEYKGEDAKEDKALK | FRNT50,Vero | 3.00 | Neutralizing | 28821561 |
| **191** | ADI-24192 | AKDRSTRGFGELLNY | DENV-4 | EDIII | SGKFSIKYEGAGATNASALT | FRNT50,Vero | 3.00 | Neutralizing | 28821561 |
| **192** | ADI-24192 | AKDRSTRGFGELLNY | DENV-2 | EDIII | TGKFKVQYEGDGSKDEKALK | FRNT50,Vero | 3.00 | Neutralizing | 28821561 |
| **193** | ADI-24227 | AKDRPPYGVGELYDY | DENV-3 | EDIII | LNTFVLEYKGEDAKEDKALK | FRNT50,Vero | 3.00 | Neutralizing | 28821561 |
| **194** | ADI-24227 | AKDRPPYGVGELYDY | DENV-4 | EDIII | SGKFSIKYEGAGATNASALT | FRNT50,Vero | 3.00 | Neutralizing | 28821561 |
| **195** | ADI-24227 | AKDRPPYGVGELYDY | DENV-2 | EDIII | TGKFKVQYEGDGSKDEKALK | FRNT50,Vero | 3.00 | Neutralizing | 28821561 |
| **196** | ADI-24227 | AKDRPPYGVGELYDY | DENV-1 | EDIII | TGSFKLKYEGTDGKEEKALK | FRNT50,Vero | 0.01 | Neutralizing | 28821561 |
| **197** | ADI-24229 | ARDRLGQGFGELFAF | DENV-1 | EDIII | TGSFKLKYEGTDGKEEKALK | FRNT50,Vero | 10.00 | Neutralizing | 28821561 |
| **198** | ADI-24232 | GKDRTARGFGELLDS | DENV-3 | EDIII | LNTFVLEYKGEDAKEDKALK | FRNT50,Vero | 3.00 | Neutralizing | 28821561 |
| **199** | ADI-24232 | GKDRTARGFGELLDS | DENV-4 | EDIII | SGKFSIKYEGAGATNASALT | FRNT50,Vero | 0.19 | Neutralizing | 28821561 |
| **200** | ADI-24232 | GKDRTARGFGELLDS | DENV-2 | EDIII | TGKFKVQYEGDGSKDEKALK | FRNT50,Vero | 0.61 | Neutralizing | 28821561 |
| **201** | ADI-24232 | GKDRTARGFGELLDS | DENV-1 | EDIII | TGSFKLKYEGTDGKEEKALK | FRNT50,Vero | 0.00 | Neutralizing | 28821561 |
| **202** | ADI-24255 | GKDRVARGFGELLDS | DENV-3 | EDIII | LNTFVLEYKGEDAKEDKALK | FRNT50,Vero | 3.00 | Neutralizing | 28821561 |
| **203** | ADI-24255 | GKDRVARGFGELLDS | DENV-4 | EDIII | SGKFSIKYEGAGATNASALT | FRNT50,Vero | 2.30 | Neutralizing | 28821561 |
| **204** | ADI-24255 | GKDRVARGFGELLDS | DENV-2 | EDIII | TGKFKVQYEGDGSKDEKALK | FRNT50,Vero | 3.00 | Neutralizing | 28821561 |
| **205** | ADI-24255 | GKDRVARGFGELLDS | DENV-1 | EDIII | TGSFKLKYEGTDGKEEKALK | FRNT50,Vero | 0.00 | Neutralizing | 28821561 |
| **206** | ANTI-DENV-10_50-1B-H1L1 | ARVTSLSGSEVYGMDV | DENV-2 | EDII | VDRGWGNGCGLFGKGGIV | NT50 | 0.04 | Neutralizing | 28422757, WO2013089647A1 |
| **207** | ANTI-DENV-10_50-1B-H1L1 | ARVTSLSGSEVYGMDV | DENV-4 | EDII | VDRGWGNGCGLFGKGGVV | NT50 | 0.03 | Neutralizing | 28422757, WO2013089647A1 |
| **208** | ANTI-DENV-10_50-1B-H1L1 | ARVTSLSGSEVYGMDV | DENV-1 | EDII | VDRGWGNGCGLFGKGSLL | NT50 | 0.38 | Neutralizing | 28422757, WO2013089647A1 |
| **209** | ANTI-DENV-10_50-1B-H1L1 | ARVTSLSGSEVYGMDV | DENV-3 | EDII | VDRGWGNGCGLFGKGSLV | NT50 | 0.37 | Neutralizing | 28422757, WO2013089647A1 |
| **210** | ANTI-DENV-10_50-1D-H8L1 | ARDPSGTYDYNYYAMDV | DENV-2 | EDII | GGLK | NT50,DC-SIGN | 0.06 | Neutralizing | 27707930, WO2013089647A1 |
| **211** | ANTI-DENV-10_50-1D-H8L1 | ARDPSGTYDYNYYAMDV | DENV-3 | EDII | GGLL | NT50,DC-SIGN | 0.12 | Neutralizing | 27707930, WO2013089647A1 |
| **212** | ANTI-DENV-10_50-1D-H8L1 | ARDPSGTYDYNYYAMDV | DENV-4 | EDII | GGLS | NT50,DC-SIGN | 0.50 | Neutralizing | 27707930, WO2013089647A1 |
| **213** | ANTI-DENV-10_50-1D-H8L1 | ARDPSGTYDYNYYAMDV | DENV-1 | EDII | GGLV | NT50,DC-SIGN | 0.60 | Neutralizing | 27707930, WO2013089647A1 |
| **214** | ANTI-DENV-10_50-2F-H1L3 | YSSGPRWGHFDI | DENV-2 | EDII | GWFA | NT50,DC-SIGN | 0.18 | Neutralizing | 27707930, WO2013089647A1 |
| **215** | ANTI-DENV-10_50-2F-H1L3 | YSSGPRWGHFDI | DENV-1 | EDII | GWFS | NT50,DC-SIGN | 0.11 | Neutralizing | 27707930, WO2013089647A1 |
| **216** | ANTI-DENV-10_50-2F-H1L3 | YSSGPRWGHFDI | DENV-3 | EDII | GWFT | NT50,DC-SIGN | 0.09 | Neutralizing | 27707930, WO2013089647A1 |
| **217** | ANTI-DENV-10_50-2F-H1L3 | YSSGPRWGHFDI | DENV-4 | EDII | GWFV | NT50,DC-SIGN | 0.07 | Neutralizing | 27707930, WO2013089647A1 |
| **218** | ANTI-DENV-10_50-3H-H1L1 | AKDRQSLHDPGSGSYVALDH | DENV-1 | EDII | GWFS | NT50,DC-SIGN | 0.39 | Neutralizing | 27707930, WO2013089647A1 |
| **219** | ANTI-DENV-10_50-3H-H1L1 | AKDRQSLHDPGSGSYVALDH | DENV-3 | EDII | GWFT | NT50,DC-SIGN | 0.87 | Neutralizing | 27707930, WO2013089647A1 |
| **220** | ANTI-DENV-10_50-3H-H1L1 | AKDRQSLHDPGSGSYVALDH | DENV-4 | EDII | GWFV | NT50,DC-SIGN | 0.04 | Neutralizing | 27707930, WO2013089647A1 |
| **221** | ANTI-DENV-10_50-6E-H1L1 | AGGGIVSTMGAFGF | DENV-3 | EDII | GWF | NT50,DC-SIGN | 0.01 | Neutralizing | 27707930, WO2013089647A1 |
| **222** | ANTI-DENV-10_50-6E-H1L1 | AGGGIVSTMGAFGF | DENV-3 | EDII | WLFL | NT50 | 0.01 | Neutralizing | 27707930, WO2013089647A1 |
| **223** | ANTI-DENV-10_50-6E-H1L1 | AGGGIVSTMGAFGF | DENV-4 | EDII | WLFS | NT50 | 0.02 | Neutralizing | 27707930, WO2013089647A1 |
| **224** | ANTI-DENV-10_50-7A-H1L1 | AGEGYFGSAAINYFAF | DENV-3 | Interdomain | CVRGHK | NT50,DC-SIGN | 0.66 | Neutralizing | 27707930, WO2013089647A1 |
| **225** | ANTI-DENV-10_50-7A-H1L1 | AGEGYFGSAAINYFAF | DENV-4 | Interdomain | RILALE | NT50,DC-SIGN | 1.63 | Neutralizing | 27707930, WO2013089647A1 |
| **226** | ANTI-DENV-10_50-7E-H1L1 | ARHDCSDTTCREYFQH | DENV-3 | Interdomain | CVRGHK | NT50,DC-SIGN | 0.14 | Neutralizing | 27707930, WO2013089647A1 |
| **227** | ANTI-DENV-10_50-7H-H1L1 | AREGMVEVTALSFLDY | DENV-3 | Interdomain | CVRGHK | NT50,DC-SIGN | 2.00 | Neutralizing | 27707930, WO2013089647A1 |
| **228** | ANTI-DENV-10_50-8F-H1L1 | ARQAVFAEATGTTIGAIDY | DENV-2 | EDII | GGLK | NT50,DC-SIGN | 1.00 | Neutralizing | 27707930, WO2013089647A1 |
| **229** | ANTI-DENV-10_50-8F-H1L1 | ARQAVFAEATGTTIGAIDY | DENV-3 | EDII | GGLL | NT50,DC-SIGN | 0.30 | Neutralizing | 27707930, WO2013089647A1 |
| **230** | ANTI-DENV-10_50-8F-H1L1 | ARQAVFAEATGTTIGAIDY | DENV-4 | EDII | GGLS | NT50,DC-SIGN | 0.50 | Neutralizing | 27707930, WO2013089647A1 |
| **231** | ANTI-DENV-10_50-8F-H1L1 | ARQAVFAEATGTTIGAIDY | DENV-1 | EDII | GGLV | NT50,DC-SIGN | 0.60 | Neutralizing | 27707930, WO2013089647A1 |
| **232** | ANTI-DENV-10_50-9E-H2L2 | ATTNWGVSVKPGTFDI | DENV-2 | EDII | WLFK | NT50,DC-SIGN | 0.40 | Neutralizing | 27707930, WO2013089647A1 |
| **233** | ANTI-DENV-10_50-9E-H2L2 | ATTNWGVSVKPGTFDI | DENV-3 | EDII | WLFL | NT50,DC-SIGN | 0.32 | Neutralizing | 27707930, WO2013089647A1 |
| **234** | ANTI-DENV-10_50-9E-H2L2 | ATTNWGVSVKPGTFDI | DENV-4 | EDII | WLFS | NT50,DC-SIGN | 0.02 | Neutralizing | 27707930, WO2013089647A1 |
| **235** | ANTI-DENV-10_50-9E-H2L2 | ATTNWGVSVKPGTFDI | DENV-1 | EDII | WLFV | NT50,DC-SIGN | 0.04 | Neutralizing | 27707930, WO2013089647A1 |
| **236** | ANTI-DENV-10_63-1D-H4L1 | AHRRWEDSTFQH | DENV-2 | EDII | GGLK | NT50,DC-SIGN | 0.23 | Neutralizing | 27707930, WO2013089647A1 |
| **237** | ANTI-DENV-10_63-1D-H4L1 | AHRRWEDSTFQH | DENV-3 | EDII | GGLL | NT50,DC-SIGN | 0.04 | Neutralizing | 27707930, WO2013089647A1 |
| **238** | ANTI-DENV-10_63-1D-H4L1 | AHRRWEDSTFQH | DENV-4 | EDII | GGLS | NT50,DC-SIGN | 2.55 | Neutralizing | 27707930, WO2013089647A1 |
| **239** | ANTI-DENV-10_63-1D-H4L1 | AHRRWEDSTFQH | DENV-1 | EDII | GGLV | NT50,DC-SIGN | 1.35 | Neutralizing | 27707930, WO2013089647A1 |
| **240** | ANTI-DENV-10_63-1E-H3L1 | ARGRGGFAATAGTDY | DENV-1 | EDII | GGLV | NT50,DC-SIGN | 10.00 | Neutralizing | 27707930, WO2013089647A1 |
| **241** | ANTI-DENV-10_63-2C-H3L2 | SQRHILTGHYTDY | DENV-3 | EDIII | HWPL | NT50,DC-SIGN | 0.24 | Neutralizing | 27707930, WO2013089647A1 |
| **242** | ANTI-DENV-10_63-2F-H1L1 | AEDTALYYCANVLGYCTPTTCYEDH | DENV-3 | EDII | WLF | NT50,DC-SIGN | 0.10 | Neutralizing | 27707930, WO2013089647A1 |
| **243** | ANTI-DENV-10_63-4F-H6L1 | TIYFYDRSGYFFDH | DENV-2 | EDII | GGLK | NT50,DC-SIGN | 0.33 | Neutralizing | 27707930, WO2013089647A1 |
| **244** | ANTI-DENV-10_63-4F-H6L1 | TIYFYDRSGYFFDH | DENV-3 | EDII | GGLL | NT50,DC-SIGN | 0.27 | Neutralizing | 27707930, WO2013089647A1 |
| **245** | ANTI-DENV-10_63-4F-H6L1 | TIYFYDRSGYFFDH | DENV-4 | EDII | GGLS | NT50,DC-SIGN | 5.00 | Neutralizing | 27707930, WO2013089647A1 |
| **246** | ANTI-DENV-10_63-4F-H6L1 | TIYFYDRSGYFFDH | DENV-1 | EDII | GGLV | NT50,DC-SIGN | 3.79 | Neutralizing | 27707930, WO2013089647A1 |
| **247** | ANTI-DENV-10_63-5A-H6L1 | ARCWRGYDPLTGYYTGYYFDY | DENV-3 | EDII | WLF | NT50,DC-SIGN | 0.05 | Neutralizing | 27707930, WO2013089647A1 |
| **248** | ANTI-DENV-10_63-5B-H1L1 | ARHHSSGNALDY | DENV-2 | EDII | GGLK | NT50,DC-SIGN | 0.21 | Neutralizing | 27707930, WO2013089647A1 |
| **249** | ANTI-DENV-10_63-5B-H1L1 | ARHHSSGNALDY | DENV-3 | EDII | GGLL | NT50,DC-SIGN | 0.14 | Neutralizing | 27707930, WO2013089647A1 |
| **250** | ANTI-DENV-10_63-5B-H1L1 | ARHHSSGNALDY | DENV-4 | EDII | GGLS | NT50,DC-SIGN | 1.17 | Neutralizing | 27707930, WO2013089647A1 |
| **251** | ANTI-DENV-10_63-5D-H1L2 | LRSDDTALYFCARTPVTGPDLDY | DENV-3 | EDII | WGL | NT50,DC-SIGN | 0.01 | Neutralizing | 27707930, WO2013089647A1 |
| **252** | ANTI-DENV-10_63-5H-H3L1 | VRQMNYYHLGSSVGFDP | DENV-2 | EDII | GGLK | NT50,DC-SIGN | 0.05 | Neutralizing | 27707930, WO2013089647A1 |
| **253** | ANTI-DENV-10_63-5H-H3L1 | VRQMNYYHLGSSVGFDP | DENV-3 | EDII | GGLL | NT50,DC-SIGN | 0.05 | Neutralizing | 27707930, WO2013089647A1 |
| **254** | ANTI-DENV-10_63-5H-H3L1 | VRQMNYYHLGSSVGFDP | DENV-4 | EDII | GGLS | NT50,DC-SIGN | 0.58 | Neutralizing | 27707930, WO2013089647A1 |
| **255** | ANTI-DENV-10_63-5H-H3L1 | VRQMNYYHLGSSVGFDP | DENV-1 | EDII | GGLV | NT50,DC-SIGN | 2.85 | Neutralizing | 27707930, WO2013089647A1 |
| **256** | ANTI-DENV-10_63-6C-H8L1 | LTSEDMAVYFCARHHRHILTGERTDY | DENV-3 | EDIII | HWDPA | NT50,DC-SIGN | 0.38 | Neutralizing | 27707930, WO2013089647A1 |
| **257** | ANTI-DENV-10_63-6E-H6L1 | ARGGHHILTGRYIDD | DENV-2 | EDII | GGLK | NT50,DC-SIGN | 0.38 | Neutralizing | 27707930, WO2013089647A1 |
| **258** | ANTI-DENV-10_63-6E-H6L1 | ARGGHHILTGRYIDD | DENV-3 | EDII | GGLL | NT50,DC-SIGN | 0.11 | Neutralizing | 27707930, WO2013089647A1 |
| **259** | ANTI-DENV-10_63-8F-H1L1 | ARQAVFAEATGTTIGAIDY | DENV-3 | EDII | WLF | NT50,DC-SIGN | 0.06 | Neutralizing | 27707930, WO2013089647A1 |
| **260** | B10 | ARQDRNWFDS | DENV-1 | EDII | LPFWGF | NT50 | 3.21 | Neutralizing | 31820734, WO2020033491A1 |
| **261** | B10 | ARQDRNWFDS | DENV-3 | EDII | LPYWGF | NT50 | 3.21 | Neutralizing | 31820734, WO2020033491A1 |
| **262** | B10 | ARQDRNWFDS | DENV-2 | EDII | YPFWGF | NT50 | 3.21 | Neutralizing | 31820734, WO2020033491A1 |
| **263** | B10 | ARQDRNWFDS | DENV-4 | EDII | YPYWGF | NT50 | 3.21 | Neutralizing | 31820734, WO2020033491A1 |
| **264** | C4 | ARADEMATAQGFYAFDI | DENV-3 | Interdomain | KHVNTNFE | NT50 | 2.47 | Neutralizing | 31820734, WO2020033491A1 |
| **265** | C4 | ARADEMATAQGFYAFDI | DENV-1 | Interdomain | KHVNTTFE | NT50 | 0.79 | Neutralizing | 31820734, WO2020033491A1 |
| **266** | C4 | ARADEMATAQGFYAFDI | DENV-4 | Interdomain | KHVNTVFE | NT50 | 2.47 | Neutralizing | 31820734, WO2020033491A1 |
| **267** | C4 | ARYCSSPSCYHNWFDP | DENV-3 | Interdomain | KHVNTNFE | NT50 | 0.04 | Neutralizing | 31820734, WO2020033491A1 |
| **268** | C4 | ARYCSSPSCYHNWFDP | DENV-1 | Interdomain | KHVNTTFE | NT50 | 0.07 | Neutralizing | 31820734, WO2020033491A1 |
| **269** | C4 | ARYCSSPSCYHNWFDP | DENV-4 | Interdomain | KHVNTVFE | NT50 | 1.62 | Neutralizing | 31820734, WO2020033491A1 |
| **270** | DB2-3 | TIRDGKGAMDY | DENV-2 | EDI | NRVE | PRNT50 | 1.20 | Neutralizing | 26135599, US8637035B2 |
| **271** | DB23-3 | ARLGGDFFADY | DENV-2 | EDI | NRVE | PRNT50 | 0.41 | Neutralizing | 26135599, US8637035B2 |
| **272** | DB25-2 | AKNFGTHYYGSNYGNFDY | DENV-2 | EDIII | EF | PRNT50 | 1.20 | Neutralizing | 26135599, US8637035B2 |
| **273** | DB32-6 | VRTGSFWYFDV | DENV-2 | EDIII | KEI | PRNT50 | 0.14 | Neutralizing | 26135599, US8637035B2 |
| **274** | DB42-3 | TREGGDDDQYYYSMDY | DENV-2 | EDI | NRVE | PRNT50 | 3.70 | Neutralizing | 26135599, US8637035B2 |
| **275** | DENV-1-E106 | RINWALDY | DENV-1 | EDIII | KKKYETDKE | PRNT50,BHK21 | 0.00 | Neutralizing | 24743696 |
| **276** | DENV-1-E106 | RINWALDY | DENV-1 | EDIII | KGTDPKEPEK | PRNT50,BHK21 | 0.00 | Neutralizing | 20369024 |
| **277** | DENV-1-E106 | RINWALDY | DENV-1 | EDIII | KGTDPREPEK | PRNT50,BHK21 | 0.03 | Neutralizing | 20369024 |
| **278** | DENV-1-E111 | WFFPWYFDV | DENV-1 | EDIII | YMTEKPKQKPS | PRNT50,BHK21 | 0.00 | Neutralizing | 20369024 |
| **279** | DENV-1-E111 | WFFPWYFDV | DENV-1 | EDIII | YVMCKIPFSSQDEKGVTQNGRLVAGEAK | PRNT50,BHK21 | 9.72 | Neutralizing | 23055922 |
| **280** | DENV-1-E111 | WFFPWYFDV | DENV-1 | EDIII | YVMCKIPFSTQDEKGATQNGRLVAGEAK | PRNT50,BHK21 | 0.00 | Neutralizing | 23055922 |
| **281** | DM25-3 | VRYGGYYVFDY | DENV-4 | Interdomain | DW | FRNT50 | 0.58 | Neutralizing | 30334522 |
| **282** | DM25-3 | VRYGGYYVFDY | DENV-1 | Interdomain | EW | FRNT50 | 0.32 | Neutralizing | 30334522 |
| **283** | DM25-3 | VRYGGYYVFDY | DENV-3 | Interdomain | KW | FRNT50 | 0.24 | Neutralizing | 30334522 |
| **284** | DM25-3 | VRYGGYYVFDY | DENV-2 | Interdomain | VW | FRNT50 | 0.38 | Neutralizing | 30334522 |
| **285** | DM8-6 | ARSLLPNWYFD | DENV-2 | EDIII | VKE | FRNT50 | 0.04 | Neutralizing | 30334522 |
| **286** | DV22.3 | ARGPPTDCSSGRCLGVGVGLDP | DENV-4 | Interdomain | KGSHVNTVF | FCNT50,Vero | 0.01 | Neutralizing | WO2010043977 |
| **287** | DV82.11 | VGVLTWPVNAEYFHH | DENV-1 | Interdomain | AKISNTTTDSRCPTQGEATLVEEQDANFVCRRTFVDRGWGNGCGLFGKGSLLTCAKFK | FCNT50,Vero | 0.04 | Neutralizing | WO2010043977 |
| **288** | DV82.11 | VGVLTWPVNAEYFHH | DENV-2 | Interdomain | AKLTNTTTESRCPTQGEPSLVEEQDKRFVCRHSMVDRGWGNGCGLFGKGGIVTCAMFT | FCNT50,Vero | 0.02 | Neutralizing | WO2010043977 |
| **289** | DV82.11 | VGVLTWPVNAEYFHH | DENV-4 | Interdomain | ASISNITTATRCPTQGEPYLVEEQDQQYICRRDVVDRGWGNGCGLFGKGGVVTCAKFA | FCNT50,Vero | 0.12 | Neutralizing | WO2010043977 |
| **290** | DV82.11 | VGVLTWPVNAEYFHH | DENV-3 | Interdomain | GKITNITTDSRCPTQGEAVLPEEQDQNYVCKHTYVDRGWGNGCGLFGKGSLVTCAKFQ | FCNT50,Vero | 0.09 | Neutralizing | WO2010043977 |
| **291** | DV87.1 | GGPRGLQLLSSWVDY | DENV-1 | EDIII | KELW | FCNT50,Vero | 0.00 | Neutralizing | 30185598 |
| **292** | DV87.1 | GGPRGLQLLSSWVDY | DENV-3 | EDIII | VEIW | FCNT50,Vero | 0.01 | Neutralizing | 30185598 |
| **293** | EDE1-(2)A2 | ARGFYSGSYYPTAPFDI | DENV-1 | EDE | EKQWVNIAPTKEQWF | ELISA | 5.00 | Neutralizing | 25501631, WO2017212291 |
| **294** | EDE1-(2)A5 | ARVFYSGSYYPNSPFDY | DENV-1 | EDE | EQWVNTIAPTKEKKWF | FCNT50,Vero | 10.00 | Neutralizing | 25501631, WO2017212291 |
| **295** | EDE1-(2)A7 | ARSLWSGELWGGPLGY | DENV-2 | EDE | EKQWVNIAPTKEIKLKQWF | FRNT50,C3/36 | 0.50 | Neutralizing | 25501631, WO2017212291 |
| **296** | EDE1-(2)B10 | ARVEGGPKYYFGSGDFYNL | DENV-2 | EDE | EKQWVNIAPTKEKQWF | FRNT50,C3/36 | 0.50 | Neutralizing | 25501631, WO2017212291 |
| **297** | EDE1-(2)B11 | ARVFYSGSYYPNSPFDS | DENV-2 | EDE | EQWIATWF | FRNT50,C3/36 | 0.50 | Neutralizing | 25501631, WO2017212291 |
| **298** | EDE1-(2)C2 | ARGYNWNDVQYYYTMDV | DENV-2 | EDE | EKQWNIAPTKELKQWF | FRNT50,C3/36 | 0.50 | Neutralizing | 25501631, WO2017212291 |
| **299** | EDE1-(2)D4 | ARGYNWNDVHYYYTMDV | DENV-2 | EDE | EKQWNIAPTKEQWF | FRNT50,C3/36 | 0.50 | Neutralizing | 25501631, WO2017212291 |
| **300** | EDE1-(3)B10 | ARPLAHTYDFWSGYHRATGYGMDV | DENV-2 | EDE | EKQWKNIATKGQRWF | FCNT50,Vero | 0.06 | Neutralizing | 25501631, WO2017212291 |
| **301** | EDE1-(3)B10 | ARPLAHTYDFWSGYHRATGYGMDV | DENV-3 | EDE | EKQWLNTATKGLRWY | FCNT50,Vero | 0.14 | Neutralizing | 25501631, WO2017212291 |
| **302** | EDE1-(3)B10 | ARPLAHTYDFWSGYHRATGYGMDV | DENV-1 | EDE | EKQWVNIATKGLQWF | FCNT50,Vero | 0.03 | Neutralizing | 25501631, WO2017212291 |
| **303** | EDE1-(3)C10 | ARDKVDDYGDYWFPTLWYFDY | DENV-2 | EDE | EKQWKNEIPQENKQWF | NT50 | 0.17 | Neutralizing | 31820734, WO2020033491A1 |
| **304** | EDE1-(3)C10 | ARDKVDDYGDYWFPTLWYFDY | DENV-2 | EDE | EKQWKNEIPQNNKQWF | NT50 | 0.17 | Neutralizing | 31820734, WO2020033491A1 |
| **305** | EDE1-(3)C10 | ARDKVDDYGDYWFPTLWYFDY | DENV-3 | EDE | EKQWLNTAPTKNVEWY | NT50 | 0.75 | Neutralizing | 31820734, WO2020033491A1 |
| **306** | EDE1-(3)C10 | ARDKVDDYGDYWFPTLWYFDY | DENV-1 | EDE | EKQWVNTAPTEKKKWF | NT50 | 0.35 | Neutralizing | 31820734, WO2020033491A1 |
| **307** | EDE1-(3)C10 | ARDKVDDYGDYWFPTLWYFDY | DENV-4 | EDE | TSQWSNTAPKKKSKWF | NT50 | 0.04 | Neutralizing | 31820734, WO2020033491A1 |
| **308** | EDE1-(3)C10 | ARDKVDDYGDYWFPTLWYFDY | DENV-3 | EDE | EKQWLNTASTKNKKWY | FCNT50,Vero | 0.28 | Neutralizing | 25501631, WO2017212291 |
| **309** | EDE1-(3)C10 | ARDKVDDYGDYWFPTLWYFDY | DENV-1 | EDE | EKQWVNIAPTKEKQWF | FCNT50,Vero | 0.08 | Neutralizing | 25501631, WO2017212291 |
| **310** | EDE1-(3)C10 | ARDKVDDYGDYWFPTLWYFDY | DENV-4 | EDE | TSQWSNTAPKKKKKWF | FCNT50,Vero | 0.01 | Neutralizing | 25501631, WO2017212291 |
| **311** | EDE1-(3)C10-(D100B-A) | ARDKVDDYGAYWFPTLWYFDY | DENV-1 | EDE | RHELNEVRWLHVKKK | FRNT50,Vero | 10.00 | Neutralizing | 34852239 |
| **312** | EDE1-(3)C10-(D100B-A) | ARDKVDDYGAYWFPTLWYFDY | DENV-4 | EDE | RHETNEVRWHVKQDDMK | FRNT50,Vero | 0.08 | Neutralizing | 34852239 |
| **313** | EDE1-(3)C10-(D100B-A) | ARDKVDDYGAYWFPTLWYFDY | DENV-2 | EDE | RHGELINEVRWITHVKKQK | FRNT50,Vero | 0.18 | Neutralizing | 34852239 |
| **314** | EDE1-(3)C10-(D98-A) | ARDKVADYGDYWFPTLWYFDY | DENV-1 | EDE | RHELNEVRWLHVKKK | FRNT50,Vero | 10.00 | Neutralizing | 34852239 |
| **315** | EDE1-(3)C10-(D98-A) | ARDKVADYGDYWFPTLWYFDY | DENV-4 | EDE | RHETNEVRWHVKQDDMK | FRNT50,Vero | 10.00 | Neutralizing | 34852239 |
| **316** | EDE1-(3)C10-(D98-A) | ARDKVADYGDYWFPTLWYFDY | DENV-2 | EDE | RHGELINEVRWITHVKKQK | FRNT50,Vero | 10.00 | Neutralizing | 34852239 |
| **317** | EDE1-(3)C10-(D99-A) | ARDKVDAYGDYWFPTLWYFDY | DENV-1 | EDE | RHELNEVRWLHVKKK | FRNT50,Vero | 10.00 | Neutralizing | 34852239 |
| **318** | EDE1-(3)C10-(D99-A) | ARDKVDAYGDYWFPTLWYFDY | DENV-4 | EDE | RHETNEVRWHVKQDDMK | FRNT50,Vero | 10.00 | Neutralizing | 34852239 |
| **319** | EDE1-(3)C10-(D99-A) | ARDKVDAYGDYWFPTLWYFDY | DENV-2 | EDE | RHGELINEVRWITHVKKQK | FRNT50,Vero | 10.00 | Neutralizing | 34852239 |
| **320** | EDE1-(3)C10-(L100H-A) | ARDKVDDYGDYWFPTAWYFDY | DENV-1 | EDE | RHELNEVRWLHVKKK | FRNT50,Vero | 10.00 | Neutralizing | 34852239 |
| **321** | EDE1-(3)C10-(P100F-A) | ARDKVDDYGDYWFETLWYFDY | DENV-1 | EDE | RHELNEVRWLHVKKK | FRNT50,Vero | 10.00 | Neutralizing | 34852239 |
| **322** | EDE1-(3)C10-(P100F-A) | ARDKVDDYGDYWFETLWYFDY | DENV-2 | EDE | RHGELINEVRWITHVKKQK | FRNT50,Vero | 10.00 | Neutralizing | 34852239 |
| **323** | EDE1-(3)C10-(W100D-A) | ARDKVDDYGDYAFPTLWYFDY | DENV-1 | EDE | RHELNEVRWLHVKKK | FRNT50,Vero | 0.54 | Neutralizing | 34852239 |
| **324** | EDE1-(3)C10-(W100D-A) | ARDKVDDYGDYAFPTLWYFDY | DENV-4 | EDE | RHETNEVRWHVKQDDMK | FRNT50,Vero | 0.08 | Neutralizing | 34852239 |
| **325** | EDE1-(3)C10-(W100D-A) | ARDKVDDYGDYAFPTLWYFDY | DENV-2 | EDE | RHGELINEVRWITHVKKQK | FRNT50,Vero | 0.18 | Neutralizing | 34852239 |
| **326** | EDE1-(3)C10-(W100D-A) | ARDKVDDYGDYAFPTLWYFDY | DENV-3 | EDE | RHGELQNEVRWLIHVKKKQ | FRNT50,Vero | 1.89 | Neutralizing | 34852239 |
| **327** | EDE1-(3)C10-(Y100-A) | ARDKVDDAGDYWFPTLWYFDY | DENV-1 | EDE | RHELNEVRWLHVKKK | FRNT50,Vero | 10.00 | Neutralizing | 34852239 |
| **328** | EDE1-(3)C10-(Y100-A) | ARDKVDDAGDYWFPTLWYFDY | DENV-4 | EDE | RHETNEVRWHVKQDDMK | FRNT50,Vero | 10.00 | Neutralizing | 34852239 |
| **329** | EDE1-(3)C10-(Y100-A) | ARDKVDDAGDYWFPTLWYFDY | DENV-2 | EDE | RHGELINEVRWITHVKKQK | FRNT50,Vero | 10.00 | Neutralizing | 34852239 |
| **330** | EDE1-2A2 | VGYSSFYYYYTMDV | DENV-2 | EDE | EKQWVNIAPTKEIKQWF | FRNT50,C3/36 | 0.50 | Neutralizing | 25501631, WO2017212291 |
| **331** | EDE1-2A5 | ARVFYSGSYYPNSPFDS | DENV-2 | EDE | EQWNIAPTAQWF | FRNT50,C3/36 | 0.50 | Neutralizing | 25501631, WO2017212291 |
| **332** | EDE1-2A5 | ARVFYSGSYYPNSPFDS | DENV-1 | EDE | EQWNIAPTEQWF | FCNT50,Vero | 10.00 | Neutralizing | 25501631, WO2017212291 |
| **333** | EDE1-2A9 | VGYSSFYYYYTMDV | DENV-2 | EDE | EKQWNIAPTEGKLKQWF | FRNT50,C3/36 | 0.50 | Neutralizing | 25501631, WO2017212291 |
| **334** | EDE1-2B11 | VGYSSFYYFYTVDV | DENV-2 | EDE | EKQWNIAPTKAKQWF | FRNT50,C3/36 | 0.50 | Neutralizing | 25501631, WO2017212291 |
| **335** | EDE1-2B2 | VGYSSFYYYYTMDV | DENV-2 | EDE | EKQWNIAPQKEKRWF | FCNT50,Vero | 0.03 | Neutralizing | 25501631, WO2017212291 |
| **336** | EDE1-2B2 | VGYSSFYYYYTMDV | DENV-1 | EDE | EKQWNIAPTKEKQWF | FCNT50,Vero | 0.05 | Neutralizing | 25501631, WO2017212291 |
| **337** | EDE1-2B2 | VGYSSFYYYYTMDV | DENV-3 | EDE | EKQWNTASTWNKKWF | FCNT50,Vero | 0.03 | Neutralizing | 25501631, WO2017212291 |
| **338** | EDE1-2B2 | VGYSSFYYYYTMDV | DENV-4 | EDE | TTQWNTAPKKKKKWF | FCNT50,Vero | 0.07 | Neutralizing | 25501631, WO2017212291 |
| **339** | EDE1-2B3 | VGYSTFYYYYTMDV | DENV-2 | EDE | EKQWVNIAPTKEGLQWF | FRNT50 | 0.50 | Neutralizing | 25501631, WO2017212291 |
| **340** | EDE1-2B4 | VGYSSFYFYYTMDV | DENV-2 | EDE | EKQWVNIAPTKELKQWF | FRNT50 | 0.50 | Neutralizing | 25501631, WO2017212291 |
| **341** | EDE1-2B7 | ARGLYSGSHYPTSPLDY | DENV-2 | EDE | EQWNIAPTEQWF | NT50 | 0.50 | Neutralizing | 25501631, WO2017212291 |
| **342** | EDE1-2B8 | VGYSNFYYYYTMDV | DENV-3 | EDE | EQWNTASTNKWY | FCNT50,Vero | 0.11 | Neutralizing | 25501631, WO2017212291 |
| **343** | EDE1-2B8 | VGYSNFYYYYTMDV | DENV-4 | EDE | TQWNTAPKKKWF | FCNT50,Vero | 0.34 | Neutralizing | 25501631, WO2017212291 |
| **344** | EDE1-2B8 | VGYSNFYYYYTMDV | DENV-2 | EDE | EQWNIASTARWF | FCNT50,Vero | 0.04 | Neutralizing | 25501631, WO2017212291 |
| **345** | EDE1-2C8 | GYSNFYYYYTMDV | DENV-1 | EDE | EQWNIAPTEQWF | PRNT50,BHK21 | 0.02 | Neutralizing | 29425203 |
| **346** | EDE1-2C8 | GYSNFYYYYTMDV | DENV-2 | EDE | EQWNIIPQEQWF | PRNT50,BHK21 | 0.08 | Neutralizing | 29425203 |
| **347** | EDE1-2C8 | GYSNFYYYYTMDV | DENV-3 | EDE | EQWNTASTNKWY | PRNT50,BHK21 | 0.35 | Neutralizing | 29425203 |
| **348** | EDE1-2C8 | GYSNFYYYYTMDV | DENV-4 | EDE | TQWNTAPKKKWF | PRNT50,BHK21 | 0.26 | Neutralizing | 29425203 |
| **349** | EDE1-2C8 | GYSNFYYYYTMDV | DENV-2 | EDE | EQWIAQRWF | PRNT50,BHK21 | 1.00 | Neutralizing | 30185598 |
| **350** | EDE1-2C8 | GYSNFYYYYTMDV | DENV-1 | EDE | EQWIATQWF | PRNT50,BHK21 | 0.13 | Neutralizing | 30185598 |
| **351** | EDE1-2C8 | GYSNFYYYYTMDV | DENV-3 | EDE | EQWTATKWY | PRNT50,BHK21 | 1.00 | Neutralizing | 30185598 |
| **352** | EDE1-2C8 | GYSNFYYYYTMDV | DENV-4 | EDE | TQWTAKKWF | PRNT50,BHK21 | 1.00 | Neutralizing | 30185598 |
| **353** | EDE1-2C8 | GYSNFYYYYTMDV | DENV-2 | EDE | EQWNIAPTERWF | FRNT50,Vero | 0.04 | Neutralizing | 25501631, WO2017212291 |
| **354** | EDE1-A12 | ARVHTGGYPPELRYYYYGMDV | DENV-2 | EDE | EKQWVNIAPTKEQWF | FCNT50,Vero | 0.50 | Neutralizing | 25501631, WO2017212291 |
| **355** | EDE1-B10 | VGYSSFYYYYTMDV | DENV-1 | EDE | EQWNIAPTQWF | FRNT50 | 0.06 | Neutralizing | 28945244 |
| **356** | EDE1-B10 | VGYSSFYYYYTMDV | DENV-2 | EDE | EQWNIASQRWF | FRNT50 | 0.07 | Neutralizing | 28945244 |
| **357** | EDE1-B10 | VGYSSFYYYYTMDV | DENV-3 | EDE | EQWNTASKKWY | FRNT50 | 0.14 | Neutralizing | 28945244 |
| **358** | EDE1-B11 | VGGYSSFYYYYTLDV | DENV-2 | EDE | EKQWNIAPTKEQWF | FRNT50 | 0.50 | Neutralizing | 25501631, WO2017212291 |
| **359** | EDE1-C9 | VGGYSSFYYYYTMDV | DENV-2 | EDE | EKQWVNIAPTKEKQWF | FRNT50 | 0.50 | Neutralizing | 25501631, WO2017212291 |
| **360** | EDE1-P6A1 | AKPAHYDDSGYPYMAYFDS | DENV-2 | EDE | EQWIAPTEQWF | FRNT50 | 0.50 | Neutralizing | 25501631, WO2017212291 |
| **361** | EDE1-P6A12 | AGRYCSSTSCSDPWTYFPH | DENV-2 | EDE | EKQWVNIAPTKEGILEQWF | FRNT50 | 0.50 | Neutralizing | 25501631, WO2017212291 |
| **362** | EDE1-P6A3 | ARRHCSSTSCSDPWTFFPS | DENV-2 | EDE | EQWVNIAPTKEQWF | FRNT50 | 0.50 | Neutralizing | 25501631, WO2017212291 |
| **363** | EDE1-P6B11 | AKEISYCGGDCQNFFFYYNMDV | DENV-2 | EDE | EKKQWVNIAPTKEGILEKQWF | FRNT50 | 0.50 | Neutralizing | 25501631, WO2017212291 |
| **364** | EDE1-P6B4 | ARWGGDCNAGSCYGPYQYRGLDA | DENV-2 | EDE | EKQWVNIAPTKEGILEQWF | FRNT50 | 0.50 | Neutralizing | 25501631, WO2017212291 |
| **365** | EDE1-P6B5 | AGRSDNWNDVYYNYALDV | DENV-2 | EDE | EKQWVNIAPTKEGILKQWF | FRNT50 | 0.50 | Neutralizing | 25501631, WO2017212291 |
| **366** | EDE1-P6C4 | ARDLGAMGYYLCSAGNCPFDY | DENV-1 | EDE | EQWIPTWF | FRNT50 | 5.00 | Neutralizing | 25501631, WO2017212291 |
| **367** | EDE1-P6C4 | ARDLGAMGYYLCSAGNCPFDY | DENV-3 | EDE | EQWTSTWY | FRNT50 | 5.00 | Neutralizing | 25501631, WO2017212291 |
| **368** | EDE1-P6C4 | ARDLGAMGYYLCSAGNCPFDY | DENV-4 | EDE | TQWTPKWY | FRNT50 | 5.00 | Neutralizing | 25501631, WO2017212291 |
| **369** | EDE2-(2)D2 | ARAHSGNYDFWSGSNYHYYYGMDV | DENV-1 | EDE | EKQWVNNTIAPTKEKQWF | FRNT50 | 5.00 | Neutralizing | 25501631, WO2017212291 |
| **370** | EDE2-(4)A10 | VRDGVRYYYDSSGYYPDSFFKYGMDV | DENV-3 | EDE | EKQWLNNTTASTKNKKWY | FRNT50 | 5.00 | Neutralizing | 25501631, WO2017212291 |
| **371** | EDE2-(4)A10 | VRDGVRYYYDSSGYYPDSFFKYGMDV | DENV-2 | EDE | EKQWVNNTIAPTKEKQWF | FRNT50 | 5.00 | Neutralizing | 25501631, WO2017212291 |
| **372** | EDE2-(4)A10 | VRDGVRYYYDSSGYYPDSFFKYGMDV | DENV-4 | EDE | TSQWSNNLTAPKKKKKWF | FRNT50 | 5.00 | Neutralizing | 25501631, WO2017212291 |
| **373** | EDE2-(4)A11 | VDGVRFYYDSTGYYPDSFFKYGMDV | DENV-2 | EDE | EKQWKNTIASQKKRWF | PRNT50,BHK21 | 0.03 | Neutralizing | 29425203 |
| **374** | EDE2-(4)A11 | VDGVRFYYDSTGYYPDSFFKYGMDV | DENV-3 | EDE | EKQWLNTTASKWKKWY | PRNT50,BHK21 | 0.03 | Neutralizing | 29425203 |
| **375** | EDE2-(4)A11 | VDGVRFYYDSTGYYPDSFFKYGMDV | DENV-4 | EDE | TSQWSNLTAPKKKQWF | PRNT50,BHK21 | 0.01 | Neutralizing | 29425203 |
| **376** | EDE2-(4)A11 | VDGVRFYYDSTGYYPDSFFKYGMDV | DENV-3 | EDE | EKQWLNTTASTWKKWY | FCNT50,Vero | 0.02 | Neutralizing | 25501631, WO2017212291 |
| **377** | EDE2-(4)A11 | VDGVRFYYDSTGYYPDSFFKYGMDV | DENV-2 | EDE | EKQWVNTIAPTKKQWF | FCNT50,Vero | 0.01 | Neutralizing | 25501631, WO2017212291 |
| **378** | EDE2-(4)A11 | VDGVRFYYDSTGYYPDSFFKYGMDV | DENV-4 | EDE | TSQWSNLTAPKKKKWF | FCNT50,Vero | 1.17 | Neutralizing | 25501631, WO2017212291 |
| **379** | EDE2-(4)A3 | ARDGVRYYYDSTGYYPDSYYEYGMDV | DENV-2 | EDE | EKQWVNTIAPTKEKQWF | FRNT50 | 5.00 | Neutralizing | 25501631, WO2017212291 |
| **380** | EDE2-(4)A3 | ARDGVRYYYDSTGYYPDSYYEYGMDV | DENV-4 | EDE | TSQWSNLTAPKKKKKWF | FRNT50 | 5.00 | Neutralizing | 25501631, WO2017212291 |
| **381** | EDE2-(4)B4 | ARATNYFDSSGYFFAPWFDP | DENV-1 | EDE | EKQWVNNTIAPTKEKKQWF | FRNT50 | 5.00 | Neutralizing | 25501631, WO2017212291 |
| **382** | EDE2-(4)B6 | ASGGGGYAGYNWFDP | DENV-1 | EDE | EKQWVNTIAPTKEKQWF | FRNT50 | 5.00 | Neutralizing | 25501631, WO2017212291 |
| **383** | EDE2-(4)B7 | ARDGVRYYYDSTGYYPDNFFQYGLDV | DENV-2 | EDE | EKQWKHTIASQKAKRWF | NT50 | 0.26 | Neutralizing | 31820734, WO2020033491A1 |
| **384** | EDE2-(4)B7 | ARDGVRYYYDSTGYYPDNFFQYGLDV | DENV-2 | EDE | EKQWKNAIASQKAKRWF | NT50 | 0.26 | Neutralizing | 31820734, WO2020033491A1 |
| **385** | EDE2-(4)B7 | ARDGVRYYYDSTGYYPDNFFQYGLDV | DENV-2 | EDE | EKQWKNTIASQNAKRWF | NT50 | 0.06 | Neutralizing | 31820734, WO2020033491A1 |
| **386** | EDE2-(4)B7 | ARDGVRYYYDSTGYYPDNFFQYGLDV | DENV-2 | EDE | EKQWKNTIISQENKRWF | NT50 | 0.06 | Neutralizing | 31820734, WO2020033491A1 |
| **387** | EDE2-(4)B7 | ARDGVRYYYDSTGYYPDNFFQYGLDV | DENV-3 | EDE | EKQWLNTTASQENKKWY | NT50 | 0.05 | Neutralizing | 31820734, WO2020033491A1 |
| **388** | EDE2-(4)B7 | ARDGVRYYYDSTGYYPDNFFQYGLDV | DENV-1 | EDE | EKQWVNTTAPQEKKQWF | NT50 | 0.09 | Neutralizing | 31820734, WO2020033491A1 |
| **389** | EDE2-(4)B7 | ARDGVRYYYDSTGYYPDNFFQYGLDV | DENV-4 | EDE | TSQWSNTTAPQENKKWF | NT50 | 0.21 | Neutralizing | 31820734, WO2020033491A1 |
| **390** | EDE2-(4)B7 | ARDGVRYYYDSTGYYPDNFFQYGLDV | DENV-2 | EDE | EKQWKNTIASQKAKRWF | FCNT50,Vero | 0.02 | Neutralizing | 25501631, WO2017212291 |
| **391** | EDE2-(4)B7 | ARDGVRYYYDSTGYYPDNFFQYGLDV | DENV-3 | EDE | EKQWVNTIAPKWMKQWY | FCNT50,Vero | 0.02 | Neutralizing | 25501631, WO2017212291 |
| **392** | EDE2-(4)B7 | ARDGVRYYYDSTGYYPDNFFQYGLDV | DENV-1 | EDE | EKQWVNTIAPTKEKQWF | FCNT50,Vero | 0.02 | Neutralizing | 25501631, WO2017212291 |
| **393** | EDE2-(4)D6 | ARDGVRFYSDSTGYYPDNYFPYGMDV | DENV-1 | EDE | EKQWVNNTIAPTKEKQWF | FRNT50 | 5.00 | Neutralizing | 25501631, WO2017212291 |
| **394** | EDE2-(4)D6 | ARDGVRFYSDSTGYYPDNYFPYGMDV | DENV-4 | EDE | TSQWSNNLTAPKKKKKWF | FRNT50 | 5.00 | Neutralizing | 25501631, WO2017212291 |
| **395** | EDE2-B2 | ARDVNFHDSSGYYRQGFFAP | DENV-1 | EDE | EKQWVNTIAPTKEKQWF | FRNT50 | 5.00 | Neutralizing | 25501631, WO2017212291 |
| **396** | EDE2-B6 | ARAHSGNYDFWSGSNYHYYYGMDV | DENV-1 | EDE | EQWVNTIAPTKQWF | FRNT50 | 5.00 | Neutralizing | 25501631, WO2017212291 |
| **397** | EDE2-B8 | VRDGVRYYYDSSGYYPDSFFKYGMDV | DENV-2 | EDE | EKQWVNNTIAPTKEGKKKQWF | FRNT50 | 5.00 | Neutralizing | 25501631, WO2017212291 |
| **398** | EDE2-B8 | VRDGVRYYYDSSGYYPDSFFKYGMDV | DENV-1 | EDE | EKQWVNNTIAPTKEGKLKQWF | FRNT50 | 5.00 | Neutralizing | 25501631, WO2017212291 |
| **399** | EDE2-C2 | TRDDGPYSGYDWPWASSMDV | DENV-1 | EDE | EKQWVNNTIAPTKKQWF | FRNT50 | 5.00 | Neutralizing | 25501631, WO2017212291 |
| **400** | EDE2-C4 | ARDGVRFYYDSTGYYPDPYFQYGLDV | DENV-2 | EDE | EQWKNTIASQKKWF | FCNT50,Vero | 1.20 | Neutralizing | 25501631, WO2017212291 |
| **401** | EDE2-C4 | ARDGVRFYYDSTGYYPDPYFQYGLDV | DENV-3 | EDE | EQWLNTTASKKKWY | FCNT50,Vero | 1.29 | Neutralizing | 25501631, WO2017212291 |
| **402** | EDE2-C4 | ARDGVRFYYDSTGYYPDPYFQYGLDV | DENV-1 | EDE | EQWVNTIAPTKQWF | FCNT50,Vero | 1.32 | Neutralizing | 25501631, WO2017212291 |
| **403** | EDE2-C4 | ARDGVRFYYDSTGYYPDPYFQYGLDV | DENV-4 | EDE | TQWVNTIAPTKQWF | FCNT50,Vero | 1.29 | Neutralizing | 25501631, WO2017212291 |
| **404** | EDE2-C7 | ARDIGHYYDSSGYFHYSFGMDV | DENV-1 | EDE | EKQWVNNTIAPTKKQWF | FRNT50 | 5.00 | Neutralizing | 25501631, WO2017212291 |
| **405** | EDE2-D11 | ARDHPTVINPTFVGSWFDP | DENV-1 | EDE | EKQWVNNTIAPTKKQWF | FRNT50 | 5.00 | Neutralizing | 25501631, WO2017212291 |
| **406** | EDE2-D5 | ARDVNFYDSSGYYREGWFDS | DENV-2 | EDE | EKQWVNNTIAPTKEIKQWF | FRNT50 | 5.00 | Neutralizing | 25501631, WO2017212291 |
| **407** | EDE2-D8 | ARDGVRYYYDSTGYYPDSYYEYGLDV | DENV-1 | EDE | EKQWVNNTIAPTKEKLKQWF | FRNT50 | 5.00 | Neutralizing | 25501631, WO2017212291 |
| **408** | H1 | ARVYYDSGGYFDS | DENV-1 | EDII | WF | NT50 | 0.04 | Neutralizing | 31820734, WO2020033491A1 |
| **409** | HMB-DV-1 | ARDPCSSTTCYFGYYAMDV | DENV-3 | EDIII | LTVKEIW | FCNT50,Vero | 0.01 | Neutralizing | WO2010043977 |
| **410** | HMB-DV-1 | ARDPCSSTTCYFGYYAMDV | DENV-2 | EDIII | TKKKELW | FCNT50,Vero | 0.58 | Neutralizing | WO2010043977 |
| **411** | HMB-DV-1 | ARDPCSSTTCYFGYYAMDV | DENV-1 | EDIII | TLKKELW | FCNT50,Vero | 0.01 | Neutralizing | WO2010043977 |
| **412** | HMB-DV-10 | ARVAEFDYVWGSFDF | DENV-4 | EDIII | SKKE | FCNT50,Vero | 0.47 | Neutralizing | WO2010043977 |
| **413** | HMB-DV-10 | ARVAEFDYVWGSFDF | DENV-2 | EDIII | TKKE | FCNT50,Vero | 0.08 | Neutralizing | WO2010043977 |
| **414** | HMB-DV-11 | ARVAEFDYVWGSFDF | DENV-4 | EDIII | SKSKELW | FCNT50,Vero | 0.47 | Neutralizing | WO2010043977 |
| **415** | HMB-DV-11 | ARVAEFDYVWGSFDF | DENV-2 | EDIII | TKKKELW | FCNT50,Vero | 0.08 | Neutralizing | WO2010043977 |
| **416** | HMB-DV-12 | ITDPGNAGSASYGMDV | DENV-2 | EDII | WF | NT50,Vero | 0.00 | Neutralizing | WO2010043977 |
| **417** | HMB-DV-13 | SRVLWDSSSTGTFDS | DENV-3 | EDIII | LTVKEIW | FCNT50,Vero | 1.51 | Neutralizing | WO2010043977 |
| **418** | HMB-DV-13 | SRVLWDSSSTGTFDS | DENV-2 | EDIII | TKKKELW | FCNT50,Vero | 3.33 | Neutralizing | WO2010043977 |
| **419** | HMB-DV-13 | SRVLWDSSSTGTFDS | DENV-1 | EDIII | TLKKELW | FCNT50,Vero | 0.99 | Neutralizing | WO2010043977 |
| **420** | HMB-DV-14 | ARAFAKNWFDP | DENV-2 | EDIII | TGK | FCNT50,Vero | 0.00 | Neutralizing | WO2010043977 |
| **421** | HMB-DV-2 | AREWAARGGIVDY | DENV-3 | EDIII | LTVKEIW | FCNT50,Vero | 0.01 | Neutralizing | WO2010043977 |
| **422** | HMB-DV-2 | AREWAARGGIVDY | DENV-1 | EDIII | TLKKELW | FCNT50,Vero | 0.01 | Neutralizing | WO2010043977 |
| **423** | HMB-DV-4 | ARQRGNWFDS | DENV-3 | EDIII | LTVKEIW | NT50,DC-SIGN | 0.61 | Neutralizing | WO2010043977 |
| **424** | HMB-DV-4 | ARQRGNWFDS | DENV-4 | EDIII | SKSKELW | FCNT50,Vero | 0.37 | Neutralizing | WO2010043977 |
| **425** | HMB-DV-4 | ARQRGNWFDS | DENV-2 | EDIII | TKKKELW | FCNT50,Vero | 0.25 | Neutralizing | WO2010043977 |
| **426** | HMB-DV-4 | ARQRGNWFDS | DENV-1 | EDIII | TLKKELW | NT50,DC-SIGN | 2.25 | Neutralizing | WO2010043977 |
| **427** | HMB-DV-5 | ATVGVLTWPVNAEYFHH | DENV-2 | Interdomain | KGSHVNTNF | FCNT50,Vero | 0.03 | Neutralizing | WO2010043977 |
| **428** | HMB-DV-5 | ATVGVLTWPVNAEYFHH | DENV-4 | Interdomain | KGSHVNTVF | FCNT50,Vero | 0.20 | Neutralizing | WO2010043977 |
| **429** | HMB-DV-5 | ATVGVLTWPVNAEYFHH | DENV-3 | Interdomain | KGTHVNTNF | FCNT50,Vero | 0.12 | Neutralizing | WO2010043977 |
| **430** | HMB-DV-5 | ATVGVLTWPVNAEYFHH | DENV-1 | Interdomain | KGTHVNTTF | FCNT50,Vero | 0.07 | Neutralizing | WO2010043977 |
| **431** | HMB-DV-6 | AKGGPRGLQLLSSWVDY | DENV-1 | EDIII | KELW | FCNT50,Vero | 0.01 | Neutralizing | WO2010043977 |
| **432** | HMB-DV-6 | AKGGPRGLQLLSSWVDY | DENV-3 | EDIII | VEIW | FCNT50,Vero | 0.01 | Neutralizing | WO2010043977 |
| **433** | HMB-DV-7 | QQANSFPPT | DENV-3 | Interdomain | WD | FCNT50,Vero | 0.33 | Neutralizing | WO2010043977 |
| **434** | HMB-DV-9 | ARGGGYSRNWYSYQNYGLDV | DENV-2 | EDIII | VP | FCNT50,Vero | 0.00 | Neutralizing | WO2010043977 |
| **435** | I7 | ARALFGLVAVASPFDN | DENV-3 | EDII | LIFQG | NT50 | 1.65 | Neutralizing | 31820734, WO2020033491A1 |
| **436** | I7 | ARALFGLVAVASPFDN | DENV-1 | EDII | LVLQG | NT50 | 1.75 | Neutralizing | 31820734, WO2020033491A1 |
| **437** | I7 | ARALFGLVAVASPFDN | DENV-4 | EDII | YILQG | NT50 | 1.65 | Neutralizing | 31820734, WO2020033491A1 |
| **438** | I7 | ARALFGLVAVASPFDN | DENV-2 | EDII | YVLQG | NT50 | 1.65 | Neutralizing | 31820734, WO2020033491A1 |
| **439** | J2 | ARADEMATIEGFYAFDI | DENV-3 | EDE | KHVNTNFE | NT50 | 0.02 | Neutralizing | 31820734, WO2020033491A1 |
| **440** | J8 | ARSCESPSCYHNWFDP | DENV-2 | Interdomain | KASHVNTNF | NT50 | 0.07 | Neutralizing | 31820734, WO2020033491A1 |
| **441** | J8 | ARSCESPSCYHNWFDP | DENV-2 | Interdomain | KGAHVNTNF | NT50 | 0.07 | Neutralizing | 31820734, WO2020033491A1 |
| **442** | J8 | ARSCESPSCYHNWFDP | DENV-2 | Interdomain | KGSHTNTNF | NT50 | 0.07 | Neutralizing | 31820734, WO2020033491A1 |
| **443** | J8 | ARSCESPSCYHNWFDP | DENV-2 | Interdomain | KGSHVNTAF | NT50 | 1.80 | Neutralizing | 31820734, WO2020033491A1 |
| **444** | J8 | ARSCESPSCYHNWFDP | DENV-2 | Interdomain | KGSHVNTNF | NT50 | 0.02 | Neutralizing | 31820734, WO2020033491A1 |
| **445** | J8 | ARSCESPSCYHNWFDP | DENV-2 | Interdomain | KGSHVNTNS | NT50 | 0.04 | Neutralizing | 31820734, WO2020033491A1 |
| **446** | J8 | ARSCESPSCYHNWFDP | DENV-4 | Interdomain | KGSHVNTVF | NT50 | 0.07 | Neutralizing | 31820734, WO2020033491A1 |
| **447** | J8 | ARSCESPSCYHNWFDP | DENV-2 | Interdomain | KGSSTHAAF | NT50 | 0.18 | Neutralizing | 31820734, WO2020033491A1 |
| **448** | J8 | ARSCESPSCYHNWFDP | DENV-2 | Interdomain | KGSSTNTNF | NT50 | 0.07 | Neutralizing | 31820734, WO2020033491A1 |
| **449** | J8 | ARSCESPSCYHNWFDP | DENV-2 | Interdomain | KGSSVNTNF | NT50 | 0.07 | Neutralizing | 31820734, WO2020033491A1 |
| **450** | J8 | ARSCESPSCYHNWFDP | DENV-3 | Interdomain | KGTHVNTNF | NT50 | 0.01 | Neutralizing | 31820734, WO2020033491A1 |
| **451** | J8 | ARSCESPSCYHNWFDP | DENV-1 | Interdomain | KGTHVNTTF | NT50 | 0.01 | Neutralizing | 31820734, WO2020033491A1 |
| **452** | J8 | ARSCESPSCYHNWFDP | DENV-2 | Interdomain | TGSHTNTNF | NT50 | 1.80 | Neutralizing | 31820734, WO2020033491A1 |
| **453** | J8 | ARSCESPSCYHNWFDP | DENV-2 | Interdomain | TGSHVNTNF | NT50 | 0.04 | Neutralizing | 31820734, WO2020033491A1 |
| **454** | J8 | ARSCESPSCYHNWFDP | DENV-2 | Interdomain | TGSHVNTNS | NT50 | 0.90 | Neutralizing | 31820734, WO2020033491A1 |
| **455** | J9 | ARYCYSASCYHNWFDP | DENV-2 | Interdomain | KASHVNTNF | NT50 | 0.12 | Neutralizing | 31820734, WO2020033491A1 |
| **456** | J9 | ARYCYSASCYHNWFDP | DENV-2 | Interdomain | KGAHVNTNF | NT50 | 0.10 | Neutralizing | 31820734, WO2020033491A1 |
| **457** | J9 | ARYCYSASCYHNWFDP | DENV-2 | Interdomain | KGSHTHTNF | NT50 | 0.12 | Neutralizing | 31820734, WO2020033491A1 |
| **458** | J9 | ARYCYSASCYHNWFDP | DENV-2 | Interdomain | KGSHTNTNF | NT50 | 0.12 | Neutralizing | 31820734, WO2020033491A1 |
| **459** | J9 | ARYCYSASCYHNWFDP | DENV-2 | Interdomain | KGSHVHANS | NT50 | 0.30 | Neutralizing | 31820734, WO2020033491A1 |
| **460** | J9 | ARYCYSASCYHNWFDP | DENV-2 | Interdomain | KGSHVNTAF | NT50 | 0.12 | Neutralizing | 31820734, WO2020033491A1 |
| **461** | J9 | ARYCYSASCYHNWFDP | DENV-2 | Interdomain | KGSHVNTNF | NT50 | 0.03 | Neutralizing | 31820734, WO2020033491A1 |
| **462** | J9 | ARYCYSASCYHNWFDP | DENV-2 | Interdomain | KGSHVNTNS | NT50 | 0.03 | Neutralizing | 31820734, WO2020033491A1 |
| **463** | J9 | ARYCYSASCYHNWFDP | DENV-4 | Interdomain | KGSHVNTVF | NT50 | 0.04 | Neutralizing | 31820734, WO2020033491A1 |
| **464** | J9 | ARYCYSASCYHNWFDP | DENV-2 | Interdomain | KGSSTNTNF | NT50 | 0.03 | Neutralizing | 31820734, WO2020033491A1 |
| **465** | J9 | ARYCYSASCYHNWFDP | DENV-2 | Interdomain | KGSSVNTNF | NT50 | 0.10 | Neutralizing | 31820734, WO2020033491A1 |
| **466** | J9 | ARYCYSASCYHNWFDP | DENV-3 | Interdomain | KGTHVNTNF | NT50 | 0.02 | Neutralizing | 31820734, WO2020033491A1 |
| **467** | J9 | ARYCYSASCYHNWFDP | DENV-1 | Interdomain | KGTHVNTTF | NT50 | 0.01 | Neutralizing | 31820734, WO2020033491A1 |
| **468** | J9 | ARYCYSASCYHNWFDP | DENV-2 | Interdomain | TGSHTNTNF | NT50 | 3.00 | Neutralizing | 31820734, WO2020033491A1 |
| **469** | J9 | ARYCYSASCYHNWFDP | DENV-2 | Interdomain | TGSHVNTNF | NT50 | 0.12 | Neutralizing | 31820734, WO2020033491A1 |
| **470** | J9 | ARYCYSASCYHNWFDP | DENV-2 | Interdomain | TGSHVNTNS | NT50 | 0.30 | Neutralizing | 31820734, WO2020033491A1 |
| **471** | L8 | ARVYFDSGGYFDS | DENV-3 | EDII | WF | NT50 | 3.93 | Neutralizing | 31820734, WO2020033491A1 |
| **472** | L8 | ARVYFDSGGYFDS | DENV-1 | EDII | LFWF | NT50 | 0.36 | Neutralizing | 31820734, WO2020033491A1 |
| **473** | L8 | ARVYFDSGGYFDS | DENV-3 | EDII | LYWF | NT50 | 0.29 | Neutralizing | 31820734, WO2020033491A1 |
| **474** | L8 | ARVYFDSGGYFDS | DENV-2 | EDII | YFWF | NT50 | 0.18 | Neutralizing | 31820734, WO2020033491A1 |
| **475** | L8 | ARVYFDSGGYFDS | DENV-4 | EDII | YYWF | NT50 | 2.45 | Neutralizing | 31820734, WO2020033491A1 |
| **476** | M1 | VRQNRNWFDS | DENV-3 | EDII | WF | NT50 | 3.21 | Neutralizing | 31820734, WO2020033491A1 |
| **477** | M1 | ARYCSSASCYHKWFDP | DENV-1 | EDII | LPFWGF | NT50 | 1.30 | Neutralizing | 31820734, WO2020033491A1 |
| **478** | M1 | ARYCSSASCYHKWFDP | DENV-3 | EDII | LPYWGF | NT50 | 2.92 | Neutralizing | 31820734, WO2020033491A1 |
| **479** | M1 | ARYCSSASCYHKWFDP | DENV-2 | EDII | YPFWGF | NT50 | 1.33 | Neutralizing | 31820734, WO2020033491A1 |
| **480** | M1 | ARYCSSASCYHKWFDP | DENV-4 | EDII | YPYWGF | NT50 | 1.13 | Neutralizing | 31820734, WO2020033491A1 |
| **481** | m360.6 | VRTPYNWNDGPRGALDI | DENV-3 | EDIII | CLNTFVLIVVIGI | RVP-NT50,DC-SIGN | 1.50 | Neutralizing | 31242272 |
| **482** | m360.6 | VRTPYNWNDGPRGALDI | DENV-2 | EDIII | CTGKFKVIVVIGV | RVP-NT50,DC-SIGN | 4.20 | Neutralizing | 31242272 |
| **483** | m366.6 | ARYMAGIWTFDI | DENV-3 | EDIII | CLNTFVLIVVIGI | PRNT50,BHK21 | 5.23 | Neutralizing | 31242272 |
| **484** | m366.6 | ARYMAGIWTFDI | DENV-2 | EDIII | CTGKFKVIVVIGV | PRNT50,BHK21 | 4.57 | Neutralizing | 31242272 |
| **485** | mAb8 | GYSNFYYYYTMDA | DENV-3 | EDE | EQWTAKKWF | NT50 | 0.24 | Neutralizing | 31107637 |
| **486** | MZ1 | LDRFNWNDEGDC | DENV-2 | Interdomain | TGGKQLKGMSYSDGSPTEK | FCNT50 | 0.02 | Neutralizing | 32015557 |
| **487** | MZ2 | LDRYAWNDGGDHW | DENV-3 | Interdomain | KGE | FCNT50 | 0.16 | Neutralizing | 32015557 |
| **488** | MZ2 | LDRYAWNDGGDHW | DENV-2 | Interdomain | KGY | FCNT50 | 0.02 | Neutralizing | 32015557 |
| **489** | MZ24 | LDRFNWNDEGDCW | DENV-2 | EDIII | RKGSY | FCNT50 | 0.01 | Neutralizing | 32015557 |
| **490** | MZ4 | LDRYSWNEGGDH | DENV-3 | Interdomain | KLKGE | PRNT50 | 0.32 | Neutralizing | 32015557 |
| **491** | MZ4 | LDRYSWNEGGDH | DENV-2 | Interdomain | QLKGY | PRNT50 | 0.02 | Neutralizing | 32015557 |
| **492** | MZ4 | LDRYSWNEGGDH | DENV-4 | Interdomain | RLKGR | PRNT50 | 9.82 | Neutralizing | 32015557 |
| **493** | MZ4 | LDRYSWNEGGDH | DENV-1 | Interdomain | TLKGY | PRNT50 | 4.67 | Neutralizing | 32015557 |
| **494** | N2 | ARADEMATTGGFYAFDI | DENV-3 | EDE | KHVNTNFE | NT50 | 1.97 | Neutralizing | 31820734, WO2020033491A1 |
| **495** | P4(403) | ATGGPNFWSGHNWLDP | DENV-3 | EDII | QG | NT50 | 0.36 | Neutralizing | 31820734, WO2020033491A1 |
| **496** | SIGN-3C | ARGGRALFYDSYTTPRDGGSWWFDP | DENV-3 | Interdomain | GWKK | PRNT50,BHK21 | 0.47 | Neutralizing | 29263863 |
| **497** | SIGN-3C | ARGGRALFYDSYTTPRDGGSWWFDP | DENV-1 | Interdomain | GWKQ | PRNT50,BHK21 | 0.06 | Neutralizing | 29263863 |
| **498** | SIGN-3C | ARGGRALFYDSYTTPRDGGSWWFDP | DENV-2 | Interdomain | GWKR | PRNT50,BHK21 | 0.02 | Neutralizing | 29263863 |
| **499** | SMZAb2 | ARVRYYGSGTYYGGDAFDF | DENV-2 | EDII | WGLFK | ELISA | 0.12 | Neutralizing | 28978754 |
| **500** | SMZAb2 | ARVRYYGSGTYYGGDAFDF | DENV-3 | EDII | WGLFL | ELISA | 0.37 | Neutralizing | 28978754 |
| **501** | SMZAb2 | ARVRYYGSGTYYGGDAFDF | DENV-1 | EDII | WGLFV | FRNT50 | 0.12 | Neutralizing | 28978754 |
| **502** | SMZAb5 | AKDRSTRGFGELLNY | DENV-1 | EDIII | TGSFKLKYEGTDGKEEKALK | FRNT50 | 0.01 | Neutralizing | 28978754 |
| **503** | VIS513 | ARGWEGFAY | DENV-2 | EDIII | KFKIDKEIRQEGSDPNGLKLNW | PRNT50,BHK21 | 0.02 | Neutralizing | 29425203 |
| **504** | VIS513 | ARGWEGFAY | DENV-3 | EDIII | KFSLDKEMKKEGANVNSLTLHW | PRNT50,BHK21 | 0.22 | Neutralizing | 29425203 |
| **505** | VIS513 | ARGWEGFAY | DENV-1 | EDIII | SFKIDKEAQKEGAEPNKLKLSW | PRNT50,BHK21 | 0.05 | Neutralizing | 29425203 |
| **506** | VIS513 | ARGWEGFAY | DENV-4 | EDIII | TFVIDKEVKEKGVEPNNLKINW | PRNT50,BHK21 | 0.06 | Neutralizing | 29425203 |
| **507** | VIS513 | ARGWEGFAY | DENV-2 | EDIII | KKIDKEQEKDLKLNW | PRNT50,BHK21 | 0.02 | Neutralizing | 29855525 |
| **508** | VIS513 | ARGWEGFAY | DENV-3 | EDIII | KSLDKEKEKNLTLHW | PRNT50,BHK21 | 0.22 | Neutralizing | 29855525 |
| **509** | VIS513 | ARGWEGFAY | DENV-1 | EDIII | SKIDKEKEKELKLSW | PRNT50,BHK21 | 0.05 | Neutralizing | 29855525 |
| **510** | VIS513 | ARGWEGFAY | DENV-4 | EDIII | TVIDKEEKTELKINW | PRNT50,BHK21 | 0.06 | Neutralizing | 29855525 |
| **511** | Z004 | AKDRGPRGVGELFDS | DENV-1 | EDIII | VMCTGSEGTDPQKAGEK | PRNT50 | 0.02 | Neutralizing | 30403995 |
| **512** | Z004-IGL | AKDRGPRGVGELFDY | DENV-1 | EDIII | VMCTGSEGTDPQKAGEK | PRNT50 | 1.40 | Neutralizing | 32321830 |
| **513** | Z021 | NQPGGRAFDY | DENV-1 | EDIII | SYVMTGYGTDAPKEAGEK | PRNT50 | 0.01 | Neutralizing | 30403995 |
| **514** | Z032 | AKDRLNGGFGELFAS | DENV-1 | EDIII | VMCTGSEGTDPQKAGEK | NT50,DC-SIGN | 0.00 | Neutralizing | 28475892 |
| **515** | Z5 | VEVAPPEK | DENV-1 | EDII | FVDRGWG | FCNT50 | 3.45 | Neutralizing | 27974667 |
| **516** | Z5 | VEVAPPEK | DENV-2 | EDII | MVDRGWG | FCNT50 | 0.30 | Neutralizing | 27974667 |
| **517** | Z5 | VEVAPPEK | DENV-4 | EDII | VVDRGWG | FCNT50 | 1.05 | Neutralizing | 27974667 |
| **518** | Z5 | VEVAPPEK | DENV-3 | EDII | YVDRGWG | FCNT50 | 0.35 | Neutralizing | 27974667 |
| **519** | Z6 | ARQWGNYFDH | DENV-4 | EDII | PTYWCGLFGK | NT50 | 6.25 | Neutralizing | 34267374 |
| **520** | ZIKV-116 | DRLSRGVGELYDS | DENV-1 | EDIII | VMTGDGAAGDK | PRNT50 | 0.07 | Neutralizing | 31757867 |
| **521** | ZIKV-116 | DRLSRGVGELYDS | DENV-1 | EDIII | VMTGDGAAGEK | PRNT50 | 0.02 | Neutralizing | 31757867 |
| **522** | ZIKV-116 | DRLSRGVGELYDS | DENV-1 | EDIII | VMTGDQGAAGDK | PRNT50 | 0.03 | Neutralizing | 31757867 |
| **523** | ZIKV-116 | DRLSRGVGELYDS | DENV-1 | EDIII | VMTGDQGAAGEK | PRNT50 | 0.15 | Neutralizing | 31757867 |
| **524** | ZK12-2 | ARQSASTGTMAVGSFDY | DENV-2 | Interdomain | KGSHVNTNF | PRNT50,Vero | 0.22 | Neutralizing | 28614803 |
| **525** | ZK12G2 | ARQFGNYFNS | DENV-2 | Interdomain | KGSHVNTNF | PRNT50,Vero | 1.02 | Neutralizing | 28614803 |
| **526** | ZK12G2 | ARQFGNYFNS | DENV-1 | Interdomain | KGTHVNTTF | PRNT50,Vero | 0.01 | Neutralizing | 28614803 |
| **527** | ZK17F11 | ARQFGNYFNY | DENV-2 | Interdomain | KGSHVNTNF | PRNT50,Vero | 7.12 | Neutralizing | 28614803 |
| **528** | ZK17F11 | ARQFGNYFNY | DENV-1 | Interdomain | KGTHVNTTF | PRNT50,Vero | 0.48 | Neutralizing | 28614803 |
| **529** | ZK1G4 | FVVVIPGREQFFDY | DENV-2 | Interdomain | KGSHVNTNF | PRNT50,Vero | 2.83 | Neutralizing | 28614803 |
| **530** | ZK1G4 | FVVVIPGREQFFDY | DENV-1 | Interdomain | KGTHVNTTF | PRNT50,Vero | 1.80 | Neutralizing | 28614803 |
| **531** | ZK21F2 | TNPVWSADSGGSVGAFHH | DENV-2 | Interdomain | KGSHVNTNF | PRNT50,Vero | 1.84 | Neutralizing | 28614803 |
| **532** | ZK21F2 | TNPVWSADSGGSVGAFHH | DENV-1 | Interdomain | KGTHVNTTF | PRNT50,Vero | 0.42 | Neutralizing | 28614803 |
| **533** | ZK22F6 | ARQFGNFFDY | DENV-2 | Interdomain | KGSHVNTNF | PRNT50,Vero | 0.06 | Neutralizing | 28614803 |
| **534** | ZK22F6 | ARQFGNFFDY | DENV-1 | Interdomain | KGTHVNTTF | PRNT50,Vero | 0.08 | Neutralizing | 28614803 |
| **535** | ZK2B3 | ARQFGNYFDY | DENV-2 | Interdomain | KGSHVNTNF | PRNT50,Vero | 4.78 | Neutralizing | 28614803 |
| **536** | ZK2B3 | ARQFGNYFDY | DENV-1 | Interdomain | KGTHVNTTF | PRNT50,Vero | 0.53 | Neutralizing | 28614803 |
| **537** | ZK3-1 | ANTIWSADSGGSVGAFHS | DENV-2 | Interdomain | KGSHVNTNF | PRNT50,Vero | 0.77 | Neutralizing | 28614803 |
| **538** | ZK3-1 | ANTIWSADSGGSVGAFHS | DENV-1 | Interdomain | KGTHVNTTF | PRNT50,Vero | 0.17 | Neutralizing | 28614803 |
| **539** | ZK5-2 | ANTIWSVDSGGSVGAFHY | DENV-2 | Interdomain | KGSHVNTNF | PRNT50,Vero | 1.77 | Neutralizing | 28614803 |
| **540** | ZK5-2 | ANTIWSVDSGGSVGAFHY | DENV-1 | Interdomain | KGTHVNTTF | PRNT50,Vero | 0.30 | Neutralizing | 28614803 |
| **541** | ZK8-4 | VRQEKNWFDS | DENV-2 | Interdomain | KGSHVNTNF | PRNT50,Vero | 2.10 | Neutralizing | 28614803 |
| **542** | ZK8-4 | VRQEKNWFDS | DENV-1 | Interdomain | KGTHVNTTF | PRNT50,Vero | 0.67 | Neutralizing | 28614803 |
| **543** | ZKA117 | ARLNDGSTVTTSSYFDY | DENV-1 | EDII | NNK | NT50,Vero | 0.08 | Neutralizing | 27417494 |
| **544** | ZKA3 | VKDLAVLESDRLEVDQ | DENV-1 | Interdomain | KGTHVNTTF | NT50,Vero | 0.35 | Neutralizing | 27417494 |
| **545** | ZKA4 | ARGPVPYWSGESYSGAYFDF | DENV-1 | Interdomain | KGTHVNTTF | NT50,Vero | 0.59 | Neutralizing | 27417494 |
| **546** | ZKA7 | VKDFWSGDQSLESDF | DENV-1 | Interdomain | KGTHVNTTF | NT50,Vero | 0.51 | Neutralizing | 27417494 |
| **547** | ZKA78 | VKDLAIPESYRIEADY | DENV-1 | Interdomain | KGTHVNTTF | NT50,Vero | 0.27 | Neutralizing | 27417494 |
| **548** | ZKA8 | VKDFGTSPQTDF | DENV-1 | Interdomain | KGTHVNTTF | NT50,Vero | 0.10 | Neutralizing | 27417494 |
| **549** | ZWT.1 | ARGGNDYSMDY | DENV-1 | EDII | PTTWCGLFGK | NT50 | 10.00 | Neutralizing | 34267374 |
| **550** | ZWT.2 | ARGGDSYSMDY | DENV-1 | EDII | PTTWCGLFGK | NT50 | 10.00 | Neutralizing | 34267374 |
| **551** | ZWT.3 | ARGGDDYGMDY | DENV-1 | EDII | PTTWCGLFGK | NT50 | 10.00 | Neutralizing | 34267374 |
| **552** | ZWT.4 | ARITVVAEDSYFDY | DENV-1 | EDII | PTTWCGLFGK | NT50 | 10.00 | Neutralizing | 34267374 |
| **553** | ZWT.5 | ARLIYGGGHMDY | DENV-1 | EDII | PTTWCGLFGK | NT50 | 10.00 | Neutralizing | 34267374 |
| **554** | ZWT.6 | ARHYRYYLDY | DENV-1 | EDII | PTTWCGLFGK | NT50 | 10.00 | Neutralizing | 34267374 |
| **555** | 3E4 | ATYYADVSSYSEY | DENV-1 | Interdomain | GLH | PRNT50 | 42.70 | Non-neutralizing | 15542643, WO2005056600 |
| **556** | 3E4 | ATYYADVSSYSEY | DENV-4 | Interdomain | GLQ | PRNT50 | 40.50 | Non-neutralizing | 15542643, WO2005056600 |
| **557** | 3E4 | ATYYADVSSYSEY | DENV-3 | Interdomain | VLH | PRNT50 | >100 | Non-neutralizing | 15542643, WO2005056600 |
| **558** | 1E5 | ARGRPYIGGDYEYDYFYGMDV | DENV-2 | EDII | AAF | FC | - | Non-neutralizing | 34155267 |
| **559** | 4E11 | SRGWEGFAY | DENV-4 | EDIII | KFSIDKEMKKEATNSVNSLTLHW | FRNT50 | >300 | Non-neutralizing | 23569282 |
| **560** | 4E11 | SRGWEGFAY | DENV-4 | EDIII | KFSIDKEMKKETNSVNLTLH | FCNT50 | 15.00 | Non-neutralizing | 22285214 |
| **561** | 4E11 | SRGWEGFAY | DENV-2 | EDIII | ALQPRYL | NT50 | >1000 | Non-neutralizing | 11457994 |
| **562** | 4E11 | SRGWEGFAY | DENV-1 | EDIII | FKLEKEVAE | NT50 | 32.13 | Non-neutralizing | 11457994 |
| **563** | 4E11 | SRGWEGFAY | DENV-2 | EDIII | RLITANPIV | ELISA | - | Non-neutralizing | 11457994 |
| **564** | 4E11 | SRGWEGFAY | DENV-2 | EDIII | WSLFLNHAE | NT50 | >76.5 | Non-neutralizing | 11457994 |
| **565** | 4E11 | SRGWEGFAY | DENV-2 | EDIII | VGAGEKALKLSWFKKGG | ELISA | - | Non-neutralizing | 11457994 |
| **566** | 3E31 | SRGSKGAMDY | DENV-4 | EDIII | AETQHTVKISEE | PRNT50 | 50.00 | Non-neutralizing | 29249606 |
| **567** | 3E31 | SRGSKGAMDY | DENV-2 | EDIII | AETQHTVRITEE | PRNT50 | 226.8 | Non-neutralizing | 29249606 |
| **568** | 3E31 | SRGSKGAMDY | DENV-3 | EDIII | SETQHTLKITEE | PRNT50 | 18.90 | Non-neutralizing | 29249606 |
| **569** | 3E31 | SRGSKGAMDY | DENV-4 | EDIII | DKEMAEHKIHWHFRKGK | ELISPOT-MNT | 41.00 | Non-neutralizing | 23851440 |
| **570** | 14C10 | GIAGGWAFW | DENV-4 | EDE | KEQILEGVTPSEVLDGIKNS | NT50 | - | Non-neutralizing | 29425203 |
| **571** | 14C10 | GIAGGWAFW | DENV-3 | EDE | TQQQLKTASQSILEGGLKDK | NT50 | >10 | Non-neutralizing | 29425203 |
| **572** | 14C10 | GIAGGWAFW | DENV-1 | EDE | TNQYLEGTTPQEILRGTLKEK | NT50 | - | Non-neutralizing | 29425203 |
| **573** | 14C10 | GIAGGWAFW | DENV-2 | EDE | TNQPLKGTTPQEILTGVKPG | FCNT50,Vero | 50.00 | Non-neutralizing | 30895307 |
| **574** | 14C10 | GIAGGWAFW | DENV-3 | EDE | TQQYLKGVTPQEALGGLKDN | FCNT50,Vero | >10 | Non-neutralizing | 30895307 |
| **575** | 14C10 | GIAGGWAFW | DENV-4 | EDE | KEQILEGVTPREVLDGIKNS | FRNT50 | - | Non-neutralizing | 28251184 |
| **576** | 14C10 | GIAGGWAFW | DENV-4 | EDE | KEQILEGVTPSEVLDGIKDS | PRNT50,BHK21 | 30.00 | Non-neutralizing | 22723463 |
| **577** | 14C10 | GIAGGWAFW | DENV-3 | EDE | TQQMLKGVTSQEALGGLKDK | PRNT50,BHK21 | 30.00 | Non-neutralizing | 22723463 |
| **578** | 1A10 | ATYYADGSSYSEY | DENV-4 | Interdomain | GLQ | PRNT50 | 12.60 | Non-neutralizing | 15542643, WO2005056600 |
| **579** | 1A10 | ATYYADGSSYSEY | DENV-3 | Interdomain | VLH | PRNT50 | 26.30 | Non-neutralizing | 15542643, WO2005056600 |
| **580** | 1A10H7 | ARSRYYYDSDASNYGMDV | DENV-2 | EDII | SSCLSSSK | ELISA | - | Non-neutralizing | 23545366, WO2013035345, WO2014064943A1 |
| **581** | 1A1D | ARDYEGFAY | DENV-4 | EDIII | KFSIDKVKTLW | ELISA | - | Non-neutralizing | 26905804 |
| **582** | 1A1D-2 | ARDYEGFAY | DENV-1 | Interdomain | GLDEP | ES-ELISA | - | Non-neutralizing | 23162552 |
| **583** | 1A1D-2 | ARDYEGFAY | DENV-1 | Interdomain | GLDKQ | ES-ELISA | - | Non-neutralizing | 23162552 |
| **584** | 1A1D-2 | ARDYEGFAY | DENV-4 | Interdomain | GLKEV | FRNT50,Vero | 20.00 | Non-neutralizing | 23162552 |
| **585** | 1A1D-2 | ARDYEGFAY | DENV-2 | Interdomain | RDDKQ | ES-ELISA | - | Non-neutralizing | 23162552 |
| **586** | 1A1D-2 | ARDYEGFAY | DENV-2 | EDIII | GEFEVVEIRQPKLN | FCNT50 | - | Non-neutralizing | 22278250 |
| **587** | 1A1D-2 | ARDYEGFAY | DENV-4 | EDIII | SKM | PRNT50 | - | Non-neutralizing | 20832836 |
| **588** | 1A1D-2 | ARDYEGFAY | DENV-4 | EDIII | GKSK | FC,DC-SIGN | - | Non-neutralizing | 17881453 |
| **589** | 1A1D-2 | ARDYEGFAY | DENV-2 | EDIII | TFVLKKVEPKN | PRNT50,BHK21 | 50.00 | Non-neutralizing | 18264114 |
| **590** | 1A5 | AREYCTGDTCFAHFDY | DENV-4 | Interdomain | CGLQ | PRNT50 | 12.00 | Non-neutralizing | 15542643, WO2005056600 |
| **591** | 1A5 | AREYCTGDTCFAHFDY | DENV-4 | Interdomain | CGFH | PRNT50 | 50.00 | Non-neutralizing | 15542644, WO2005056600 |
| **592** | 1B2 | ARAVTAGMPAAGTLDH | DENV-4 | Interdomain | GLQ | PRNT50 | 29.20 | Non-neutralizing | 15542643, WO2005056600 |
| **593** | 1B2 | ARAVTAGMPAAGTLDH | DENV-3 | Interdomain | VLH | PRNT50 | >100 | Non-neutralizing | 15542643, WO2005056600 |
| **594** | 1B3B9 | TTLSGYSADWPEDY | DENV-2 | EDII | SSASSSK | ELISA | - | Non-neutralizing | 23545366, WO2013035345, WO2014064943A1 |
| **595** | 1B3B9 | TTLSGYSADWPEDY | DENV-2 | EDII | SSSAAASASSK | ELISA | - | Non-neutralizing | 23545366, WO2013035345, WO2014064943A1 |
| **596** | 1C19 | CAAGRRLTFAYW | DENV-3 | EDII | AAE | FCNT50,U937-DC-SIGNorVero | - | Non-neutralizing | 24255124 |
| **597** | 1C19 | CAAGRRLTFAYW | DENV-4 | EDII | QDV | FCNT50,U937-DC-SIGNorVero | - | Non-neutralizing | 24255124 |
| **598** | 1C1G4 | ARGPDYESSDSPWFDY | DENV-2 | EDII | SSSAAASASSK | ELISA | - | Non-neutralizing | 23545366, WO2013035345, WO2014064943A1 |
| **599** | 1C2D2 | ARVAKLFGSATYGMDV | DENV-2 | EDII | SSSNKLSLSSK | ELISA | - | Non-neutralizing | 23545366, WO2013035345, WO2014064943A1 |
| **600** | 1C3 | ARDSGWYDF | DENV-2 | EDII | AL | FC | - | Non-neutralizing | 34155267 |
| **601** | 1E7B8 | ARHRAVAGGDSDHDENNWFGP | DENV-1 | EDII | CCCDRWCFCCV | VN50 | 10.10 | Non-neutralizing | 23545366, WO2013035345, WO2014064943A1 |
| **602** | 1F11 | AKDFLYYYDTNGDTGH | DENV-2 | EDII | AA | NT50 | - | Non-neutralizing | 34155267 |
| **603** | 1F4 | DKNPGTKPYYHYGMDV | DENV-3 | Interdomain | QLEKELA | ELISA | - | Non-neutralizing | 26962223 |
| **604** | 1F4 | DKNPGTKPYYHYGMDV | DENV-2 | Interdomain | IKEKQETTGKKEITKITEAETGSSGN | FCNT50,Vero | >10 | Non-neutralizing | 30895307 |
| **605** | 1F4 | DKNPGTKPYYHYGMDV | DENV-2 | Interdomain | IKS | FCNT50,DC-SIGN | >10 | Non-neutralizing | 22499787 |
| **606** | 1F4 | DKNPGTKPYYHYGMDV | DENV-3 | Interdomain | QKG | FCNT50,DC-SIGN | >10 | Non-neutralizing | 22499787 |
| **607** | 1F4 | DKNPGTKPYYHYGMDV | DENV-4 | Interdomain | TKG | FCNT50,DC-SIGN | >10 | Non-neutralizing | 22499787 |
| **608** | 1F4 | DKNPGTKPYYHYGMDV | DENV-3 | Interdomain | QKETQKTTQEVTAETTVEAIPESGTT | PRNT50 | - | Non-neutralizing | 24421336 |
| **609** | 1F4 | DKNPGTKPYYHYGMDV | DENV-4 | Interdomain | TKTTEETTSNVTAMTSVEVKPDSDGN | PRNT50 | - | Non-neutralizing | 24421336 |
| **610** | 1G6 | DKNPGTKPYYHYGMDV | DENV-4 | EDIII | ALGLG | ELISA | - | Non-neutralizing | 26430770 |
| **611** | 1G6 | DKNPGTKPYYHYGMDV | DENV-3 | EDIII | ALKIN | ELISA | - | Non-neutralizing | 26430770 |
| **612** | 1G6 | DKNPGTKPYYHYGMDV | DENV-2 | EDIII | QLKLN | ELISA | - | Non-neutralizing | 26430770 |
| **613** | 1G6 | DKNPGTKPYYHYGMDV | DENV-1 | EDIII | ALKL.S | ELISA | - | Non-neutralizing | 26430770 |
| **614** | 1G7C2 | ATLIAVAGSEGAGSFDI | DENV-2 | EDII | SSAAASASSK | ELISA | - | Non-neutralizing | 23545366, WO2013035345, WO2014064943A1 |
| **615** | 1M7 | CARFGPPYSQEEGVFHGAFDVW | DENV-2 | EDII | RC | FCNT50,Vero | - | Non-neutralizing | 24255124 |
| **616** | 1N5 | CAGEMATVFEYFQYW | DENV-2 | EDII | RPR | FCNT50,vero | - | Non-neutralizing | 24255124 |
| **617** | 2C8 | AKWDGHYFDY | DENV-3 | EDIII | MSYAMLGEDAPKKIGD | FRNT50 | - | Non-neutralizing | 30323338 |
| **618** | 2C8 | AKWDGHYFDY | DENV-4 | EDIII | MSYTMSGAGAPKTVGD | FRNT50 | - | Non-neutralizing | 30323338 |
| **619** | 2C8 | AKWDGHYFDY | DENV-1 | EDIII | MSYVMTGTDAPKKAGE | FRNT50 | - | Non-neutralizing | 30323338 |
| **620** | 2D1G5 | ARAGPIAATGVQYEMDV | DENV-3 | EDII | CCCDRWCFCCL | VN50 | 12.00 | Non-neutralizing | 23545366, WO2013035345, WO2014064943A1 |
| **621** | 2D1G5 | ARAGPIAATGVQYEMDV | DENV-4 | EDII | CCCDRWCFCCS | VN50 | 16.40 | Non-neutralizing | 23545366, WO2013035345, WO2014064943A1 |
| **622** | 2D1G5 | ARAGPIAATGVQYEMDV | DENV-1 | EDII | CCCDRWCFCCV | VN50 | 18.80 | Non-neutralizing | 23545366, WO2013035345, WO2014064943A1 |
| **623** | 2D22 | RPQSIFDWNFDL | DENV-1 | EDIII | KEKKEEKP | PRNT50 | - | Non-neutralizing | 26138979 |
| **624** | 2D22 | RPQSIFDWNFDL | DENV-4 | EDIII | SDKKENSV | PRNT50 | >10 | Non-neutralizing | 26138979 |
| **625** | 2D22 | RPQSIFDWNFDL | DENV-3 | EDIII | VKKEKEEP | PRNT50 | - | Non-neutralizing | 26138979 |
| **626** | 2D22 | RPQSIFDWNFDL | DENV-4 | EDIII | SDKQGKNSV | FRNT50 | - | Non-neutralizing | 26463165 |
| **627** | 2D73 | RGYRYDGAHFDY | DENV-1 | EDIII | ETQDGT | ELISA | - | Non-neutralizing | 23851440 |
| **628** | 2D73 | RGYRYDGAHFDY | DENV-1 | EDIII | PTTYGT | ELISA | - | Non-neutralizing | 23851440 |
| **629** | 2D73 | RGYRYDGAHFDY | DENV-1 | EDIII | TTINGT | ELISA | - | Non-neutralizing | 23851440 |
| **630** | 2D73 | RGYRYDGAHFDY | DENV-1 | EDIII | AETQHGTLITANEE | ELISPOT-MNT | 143.00 | Non-neutralizing | 23851440 |
| **631** | 2D73 | RGYRYDGAHFDY | DENV-4 | EDIII | AETQHGTVISSNEE | ELISPOT-MNT | 21.00 | Non-neutralizing | 23851440 |
| **632** | 2H12 | TRGGSHAMDY | DENV-4 | EDIII | KAETQHVISE | FRNT50,Vero | 21.75 | Non-neutralizing | 22491255 |
| **633** | 2H12 | TRGGSHAMDY | DENV-2 | EDIII | KAETQHVIVE | ELISA | - | Non-neutralizing | 22491255 |
| **634** | 2H5 | AREYCTGDTCFAHFDY | DENV-3 | Interdomain | VLH | PRNT50 | 20.80 | Non-neutralizing | 15542643, WO2005056600 |
| **635** | 2H8G1 | ATGGGRFSGSGNYYYYGMDV | DENV-3 | EDII | CCCDRWCFCCL | VN50 | 25.00 | Non-neutralizing | 23545366, WO2013035345, WO2014064943A1 |
| **636** | 2H8G1 | ATGGGRFSGSGNYYYYGMDV | DENV-1 | EDII | CCCDRWCFCCV | VN50 | 16.80 | Non-neutralizing | 23545366, WO2013035345, WO2014064943A1 |
| **637** | 3A1E2 | ARGMTGFTTSNTESFDL | DENV-2 | EDII | SSSAAASASSK | ELISA | - | Non-neutralizing | 23545366, WO2013035345, WO2014064943A1 |
| **638** | 3B6C7 | ASPGGLISDEAMAGYFDY | DENV-3 | EDII | CCRWCFCCL | VN50 | 12.20 | Non-neutralizing | 23545366, WO2013035345, WO2014064943A1 |
| **639** | 3B6C7 | ASPGGLISDEAMAGYFDY | DENV-1 | EDII | CCCDRWCFCCV | VN50 | 11.70 | Non-neutralizing | 23545366, WO2013035345, WO2014064943A1 |
| **640** | 3C1 | ATGRRGRYPTGSFDY | DENV-4 | EDII | DK | PRNT50 | >200 | Non-neutralizing | 15078949, WO2005056600 |
| **641** | 3H5 | ARKGGFAMDY | DENV-3 | EDIII | MLTEGDPFSRKGVGDKA | FRNT50,Vero | - | Non-neutralizing | 23162552 |
| **642** | 3H5 | ARKGGFAMDY | DENV-4 | EDIII | MTKEGDPFIETGVGNSA | FRNT50,Vero | - | Non-neutralizing | 23162552 |
| **643** | 3H5 | ARKGGFAMDY | DENV-1 | EDIII | MTSEGDPFSVKGAGEKA | FRNT50,Vero | 20.00 | Non-neutralizing | 23162552 |
| **644** | 3H5 | ARKGGFAMDY | DENV-4 | EDIII | KED | PRNT50,BHK21 | 20.00 | Non-neutralizing | 22509258 |
| **645** | 3H5 | ARKGGFAMDY | DENV-1 | EDIII | SEE | PRNT50,BHK21 | 20.00 | Non-neutralizing | 22509258 |
| **646** | 3H5 | ARKGGFAMDY | DENV-3 | EDIII | TED | PRNT50,BHK21 | 20.00 | Non-neutralizing | 22509258 |
| **647** | 3H5 | ARKGGFAMDY | DENV-2 | EDIII | KAD | FRNT50 | 12.00 | Non-neutralizing | 25351518 |
| **648** | 3H5 | ARKGGFAMDY | DENV-3 | EDII | ALKINWYRKGSS | ELISA | - | Non-neutralizing | 1634111 |
| **649** | 3H5 | ARKGGFAMDY | DENV-1 | EDII | ALKLDWFKKGSS | ELISA | - | Non-neutralizing | 1634111 |
| **650** | 3H5 | ARKGGFAMDY | DENV-4 | EDII | ALTLHWFRKGSS | ELISA | - | Non-neutralizing | 1634111 |
| **651** | 4A6F9 | ANTLWTVGSKGGFDY | DENV-1 | EDII | CCCDRWCFCCV | VN50 | 13.90 | Non-neutralizing | 23545366, WO2013035345, WO2014064943A1 |
| **652** | 4F5E1 | ARVTGGWSDY | DENV-1 | EDII | CCCDRWCFCCV | VN50 | 14.30 | Non-neutralizing | 23545366, WO2013035345, WO2014064943A1 |
| **653** | 4G2 | ARIYHYDGYFDV | DENV-3 | EDII | WF | NT50 | 25.12 | Non-neutralizing | 34155267 |
| **654** | 4G2 | ARIYHYDGYFDV | DENV-2 | Interdomain | WGGLFSP | PRNT50 | 10.70 | Non-neutralizing | 29425203 |
| **655** | 4G2 | ARIYHYDGYFDV | DENV-1 | Interdomain | GDKEP | ES-ELISA | - | Non-neutralizing | 23162552 |
| **656** | 4G2 | ARIYHYDGYFDV | DENV-1 | Interdomain | GLDEP | ES-ELISA | - | Non-neutralizing | 23162552 |
| **657** | 4G2 | ARIYHYDGYFDV | DENV-1 | Interdomain | GLDKQ | ES-ELISA | - | Non-neutralizing | 23162552 |
| **658** | 4G2 | ARIYHYDGYFDV | DENV-1 | Interdomain | GLKEQ | ES-ELISA | - | Non-neutralizing | 23162552 |
| **659** | 4G2 | ARIYHYDGYFDV | DENV-1 | Interdomain | RDDKQ | ES-ELISA | - | Non-neutralizing | 23162552 |
| **660** | 4G2 | ARIYHYDGYFDV | DENV-1 | Interdomain | RDKEP | ES-ELISA | - | Non-neutralizing | 23162552 |
| **661** | 4G2 | ARIYHYDGYFDV | DENV-2 | Interdomain | AGAKP | WB | - | Non-neutralizing | 22509258 |
| **662** | 4G2 | ARIYHYDGYFDV | DENV-1 | Interdomain | LRWEP | PRNT50,Vero | 400.00 | Non-neutralizing | 22509258 |
| **663** | 4G2 | ARIYHYDGYFDV | DENV-3 | Interdomain | LRWSV | PRNT50,Vero | 400.00 | Non-neutralizing | 22509258 |
| **664** | 4G2 | ARIYHYDGYFDV | DENV-1 | EDII | AAAA | WB | - | Non-neutralizing | 22235356 |
| **665** | 4G2 | ARIYHYDGYFDV | DENV-3 | EDII | GLFGKGSLVT | PRNT50 | - | Non-neutralizing | 10664386 |
| **666** | 4G2 | ARIYHYDGYFDV | DENV-2 | EDIII | LITVNPIV | ELISA | - | Non-neutralizing | 10664386 |
| **667** | 4G2 | ARIYHYDGYFDV | DENV-2 | Interdomain | HGTFLLNGS | ELISA | - | Non-neutralizing | 10664386 |
| **668** | 4G2 | ARIYHYDGYFDV | DENV-2 | Interdomain | SSGNLLFTGHL | PRNT50 | - | Non-neutralizing | 10664386 |
| **669** | 4G2 | ARIYHYDGYFDV | DENV-2 | Interdomain | SSGNL | ELISA | - | Non-neutralizing | 18559931 |
| **670** | 4G2 | ARIYHYDGYFDV | DENV-2 | Interdomain | CPTQGEPSLN | ELISA | - | Non-neutralizing | 18559931 |
| **671** | 4G2 | ARIYHYDGYFDV | DENV-2 | Interdomain | NTTTDSRCPT | ELISA | - | Non-neutralizing | 18559931 |
| **672** | 4G2 | ARIYHYDGYFDV | DENV-2 | Interdomain | TEAKQPATLR | ELISA | - | Non-neutralizing | 18559931 |
| **673** | 4H12C8 | TTLSGYSADWPEDY | DENV-2 | EDII | SSSAKLSLSSK | ELISA | - | Non-neutralizing | 23545366, WO2013035345, WO2014064943A1 |
| **674** | 5D9 | ARQGTGTTGVSEDPDLY | DENV-2 | EDI | ET | ELISA | - | Non-neutralizing | 15078949, WO2005056600 |
| **675** | 5D9 | ARQGTGTTGVSEDPDLY | DENV-3 | EDI | IP | ELISA | - | Non-neutralizing | 15078949, WO2005056600 |
| **676** | 5D9 | ARQGTGTTGVSEDPDLY | DENV-1 | EDI | QT | ELISA | - | Non-neutralizing | 15078949, WO2005056600 |
| **677** | 5G2D2 | STYYYDGSDLTYGMDV | DENV-2 | EDII | DRWCFCCK | FRNT50 | 100.00 | Non-neutralizing | 23545366, WO2013035345, WO2014064943A1 |
| **678** | 5G2D2 | STYYYDGSDLTYGMDV | DENV-2 | EDII | SSSAAASASSK | ELISA | - | Non-neutralizing | 23545366, WO2013035345, WO2014064943A1 |
| **679** | 5G8E3 | AVYYCARRGDYSSSAENFQH | DENV-3 | EDII | CCCDRWCFCCL | VN50 | 10.50 | Non-neutralizing | 23545366, WO2013035345, WO2014064943A1 |
| **680** | 5G8E3 | AVYYCARRGDYSSSAENFQH | DENV-1 | EDII | CCCDRWCFCCV | VN50 | 10.60 | Non-neutralizing | 23545366, WO2013035345, WO2014064943A1 |
| **681** | 5H2 | ARQGTGTTGVSEDSFDL | DENV-3 | EDI | KEIKSITEAETGGTKQ | ELISA | - | Non-neutralizing | 15078949, WO2005056600 |
| **682** | 5H2 | ARQGTGTTGVSEDSFDL | DENV-1 | EDI | TIATATSEIQTDGAKT | ELISA | - | Non-neutralizing | 15078949, WO2005056600 |
| **683** | 5H2 | ARQGTGTTGVSEDSFDL | DENV-1 | EDI | TVIAMATSEIQTDGAKT | FRNT50,C3/36 | >100 | Non-neutralizing | 28251184 |
| **684** | 5H2 | ARQGTGTTGVSEDSFDL | DENV-3 | EDI | TVTAEATTEAIPEGTKE | N50 | >10 | Non-neutralizing | 29215033 |
| **685** | 5H2 | ARQGTGTTGVSEDSFDL | DENV-3 | EDI | IP | ELISA | - | Non-neutralizing | 17881450 |
| **686** | 5H2 | ARQGTGTTGVSEDSFDL | DENV-1 | EDI | QT | ELISA | - | Non-neutralizing | 17881450 |
| **687** | 5H2 | ARQGTGTTGVSEDSFDL | DENV-3 | EDI | VTAMSSTEAIPEGTKE | ELISA | - | Non-neutralizing | 22139356 |
| **688** | 5H2 | ARQGTGTTGVSEDSFDL | DENV-1 | EDI | VTAMSSTEIQTDGAKT | ELISA | - | Non-neutralizing | 22139356 |
| **689** | 5J7 | ARDKELLFSRAFDI | DENV-4 | Interdomain | AKEVALRCWGTNVQENTLKDNSIE | FRNT50,C3/36 | - | Non-neutralizing | 28251184 |
| **690** | 5J7 | ARDKELLFSRAFDI | DENV-1 | Interdomain | VTNPAVRCWGEKVQENQMLGTKLE | FRNT50,C3/36 | >10 | Non-neutralizing | 28251184 |
| **691** | 5J7 | ARDKELLFSRAFDI | DENV-4 | Interdomain | AKEVALRCWGTNVQENTLKSDKIK | FCNT50,Vero | >10 | Non-neutralizing | 22499787 |
| **692** | 5J7 | ARDKELLFSRAFDI | DENV-2 | Interdomain | AKQPATRCWGKKVQENELQNLVKE | FCNT50,Vero | >10 | Non-neutralizing | 22499787 |
| **693** | 5J7 | ARDKELLFSRAFDI | DENV-4 | Interdomain | AKEVALRCWGTNVQENTLKNMDKE | ELISA | - | Non-neutralizing | 25698059 |
| **694** | 5J7 | ARDKELLFSRAFDI | DENV-1 | Interdomain | VTNPAVRCWGEKVQENQLTTIEKE | ELISA | - | Non-neutralizing | 25698059 |
| **695** | 6A6 | CVRQEDTKGRSFEYW | DENV-3 | EDIII | NKK | ELISA,EC50 | >75 | Non-neutralizing | 31130109 |
| **696** | 7B3 | CARATDSGYDYVWGSFRYHFDSW | DENV-4 | EDIII | AATNS | ELISA,EC50 | - | Non-neutralizing | 31130109 |
| **697** | 7B3 | CARATDSGYDYVWGSFRYHFDSW | DENV-3 | EDIII | EAKEN | ELISA,EC50 | - | Non-neutralizing | 31130109 |
| **698** | 7B3 | CARATDSGYDYVWGSFRYHFDSW | DENV-1 | EDIII | TAKEK | FCNT50 | >75 | Non-neutralizing | 31130109 |
| **699** | 7G4 | TTRITTLTVISDAFDI | DENV-1 | Interdomain | GLH | PRNT50 | - | Non-neutralizing | 15078949, WO2005056600 |
| **700** | 7G4 | TTRITTLTVISDAFDI | DENV-4 | Interdomain | GLQ | PRNT50 | 121.00 | Non-neutralizing | 15078949, WO2005056600 |
| **701** | 7G4 | TTRITTLTVISDAFDI | DENV-3 | Interdomain | VLH | PRNT50 | - | Non-neutralizing | 15078949, WO2005056600 |
| **702** | A9E | ARSDFWRSGRYYYYMDV | DENV-4 | Interdomain | EDMF | FRNT50 | >10 | Non-neutralizing | 30996133 |
| **703** | A9E | ARSDFWRSGRYYYYMDV | DENV-1 | Interdomain | EDMI | ELISA | - | Non-neutralizing | 33597214 |
| **704** | A9E | ARSDFWRSGRYYYYMDV | DENV-3 | Interdomain | EEMV | ELISA | - | Non-neutralizing | 33597214 |
| **705** | ADI-24191 | AKDRPLHGFGELYDH | DENV-3 | EDIII | LNTFVLEYKGEDAKEDKALK | ELISA | - | Non-neutralizing | 28821561 |
| **706** | ADI-24191 | AKDRPLHGFGELYDH | DENV-4 | EDIII | SGKFSIKYEGAGATNASALT | ELISA | - | Non-neutralizing | 28821561 |
| **707** | ADI-24191 | AKDRPLHGFGELYDH | DENV-2 | EDIII | TGKFKVQYEGDGSKDEKALK | ELISA | - | Non-neutralizing | 28821561 |
| **708** | ADI-24197 | ARDGYNHHLGYHHYMDV | DENV-3 | EDIII | LNTFVLEYKGEDAKEDKALK | ELISA | - | Non-neutralizing | 28821561 |
| **709** | ADI-24197 | ARDGYNHHLGYHHYMDV | DENV-4 | EDIII | SGKFSIKYEGAGATNASALT | ELISA | - | Non-neutralizing | 28821561 |
| **710** | ADI-24197 | ARDGYNHHLGYHHYMDV | DENV-1 | EDIII | TGSFKLKYEGTDGKEEKALK | FRNT50,Vero | - | Non-neutralizing | 28821561 |
| **711** | ADI-24229 | ARDRLGQGFGELFAF | DENV-3 | EDIII | LNTFVLEYKGEDAKEDKALK | ELISA | - | Non-neutralizing | 28821561 |
| **712** | ADI-24229 | ARDRLGQGFGELFAF | DENV-4 | EDIII | SGKFSIKYEGAGATNASALT | ELISA | - | Non-neutralizing | 28821561 |
| **713** | ADI-24229 | ARDRLGQGFGELFAF | DENV-2 | EDIII | TGKFKVQYEGDGSKDEKALK | ELISA | - | Non-neutralizing | 28821561 |
| **714** | ADI-29997 | ARDPELFGGFDM | DENV-1 | EDII | KIISTVEDNKKCPLEWKV | ELISA | - | Non-neutralizing | 33255202 |
| **715** | ADI-29997 | ARDPELFGGFDM | DENV-3 | EDII | KITNTPEDNKTCPLSWKV | ELISA | - | Non-neutralizing | 33255202 |
| **716** | ADI-29997 | ARDPELFGGFDM | DENV-4 | EDII | SISITPKEDQKSCPLVWRV | ELISA | - | Non-neutralizing | 33255202 |
| **717** | ADI-30000 | AKDGEEVAAAFD | DENV-1 | EDII | KIISTVEDNKKCPLEWKV | ELISA | - | Non-neutralizing | 33255202 |
| **718** | ADI-30000 | AKDGEEVAAAFD | DENV-3 | EDII | KITNTPEDNKTCPLSWKV | ELISA | - | Non-neutralizing | 33255202 |
| **719** | ADI-30000 | AKDGEEVAAAFD | DENV-4 | EDII | SISITPKEDQKSCPLVWRV | ELISA | - | Non-neutralizing | 33255202 |
| **720** | ADI-30031 | ARDPELFGGFDI | DENV-1 | EDII | KIISTVEDNKKCPLEWKV | ELISA | - | Non-neutralizing | 33255202 |
| **721** | ADI-30031 | ARDPELFGGFDI | DENV-3 | EDII | KITNTPEDNKTCPLSWKV | ELISA | - | Non-neutralizing | 33255202 |
| **722** | ADI-30031 | ARDPELFGGFDI | DENV-4 | EDII | SISITPKEDQKSCPLVWRV | ELISA | - | Non-neutralizing | 33255202 |
| **723** | ADI-30056 | ARDGEETSPGSFDY | DENV-1 | EDII | KIISTVEDNKKCPLEWKV | ELISA | - | Non-neutralizing | 33255202 |
| **724** | ADI-30056 | ARDGEETSPGSFDY | DENV-3 | EDII | KITNTPEDNKTCPLSWKV | ELISA | - | Non-neutralizing | 33255202 |
| **725** | ADI-30056 | ARDGEETSPGSFDY | DENV-4 | EDII | SISITPKEDQKSCPLVWRV | ELISA | - | Non-neutralizing | 33255202 |
| **726** | ANTI-DENV-10_50-1G-H1L1 | ARAGQALFYDSSGYDPNKYDAFDI | DENV-3 | EDII | GGLL | ELISA | - | Non-neutralizing | 27707930, WO2013089647A1 |
| **727** | ANTI-DENV-10_50-1G-H1L1 | ARAGQALFYDSSGYDPNKYDAFDI | DENV-4 | EDII | GGLS | ELISA | - | Non-neutralizing | 27707930, WO2013089647A1 |
| **728** | ANTI-DENV-10_50-1G-H1L1 | ARAGQALFYDSSGYDPNKYDAFDI | DENV-1 | EDII | GGLV | ELISA | - | Non-neutralizing | 27707930, WO2013089647A1 |
| **729** | ANTI-DENV-10_50-1H-H1L1 | ARAGPALFYDSSGYDPTIYDSLEI | DENV-3 | EDII | GGLL | ELISA | - | Non-neutralizing | 27707930, WO2013089647A1 |
| **730** | ANTI-DENV-10_50-1H-H1L1 | ARAGPALFYDSSGYDPTIYDSLEI | DENV-4 | EDII | GGLS | ELISA | - | Non-neutralizing | 27707930, WO2013089647A1 |
| **731** | ANTI-DENV-10_50-1H-H1L1 | ARAGPALFYDSSGYDPTIYDSLEI | DENV-1 | EDII | GGLV | ELISA | - | Non-neutralizing | 27707930, WO2013089647A1 |
| **732** | ANTI-DENV-10_50-3H-H1L1 | AKDRQSLHDPGSGSYVALDH | DENV-2 | EDII | GWFA | NT50,DC-SIGN | 11.50 | Non-neutralizing | 27707930, WO2013089647A1 |
| **733** | ANTI-DENV-10_50-5A-H1L1 | ALSLQTLVRGLVYINFDY | DENV-3 | EDII | GGLL | ELISA | - | Non-neutralizing | 27707930, WO2013089647A1 |
| **734** | ANTI-DENV-10_50-5A-H1L1 | ALSLQTLVRGLVYINFDY | DENV-4 | EDII | GGLS | ELISA | - | Non-neutralizing | 27707930, WO2013089647A1 |
| **735** | ANTI-DENV-10_50-5A-H1L1 | ALSLQTLVRGLVYINFDY | DENV-1 | EDII | GGLV | ELISA | - | Non-neutralizing | 27707930, WO2013089647A1 |
| **736** | ANTI-DENV-10_50-5C-H6L1 | ARAGVALFYDSSGYDPTPYDAFEV | DENV-1 | EDII | GWFS | NT50,DC-SIGN | - | Non-neutralizing | 27707930, WO2013089647A1 |
| **737** | ANTI-DENV-10_50-5C-H6L1 | ARAGVALFYDSSGYDPTPYDAFEV | DENV-3 | EDII | GWFT | NT50,DC-SIGN | - | Non-neutralizing | 27707930, WO2013089647A1 |
| **738** | ANTI-DENV-10_50-5C-H6L1 | ARAGVALFYDSSGYDPTPYDAFEV | DENV-4 | EDII | GWFV | NT50,DC-SIGN | - | Non-neutralizing | 27707930, WO2013089647A1 |
| **739** | ANTI-DENV-10_50-6B-H1L2 | ARGGRAYYYDSTTPRDGESW | DENV-3 | EDII | GGLL | ELISA | - | Non-neutralizing | 27707930, WO2013089647A1 |
| **740** | ANTI-DENV-10_50-6B-H1L2 | ARGGRAYYYDSTTPRDGESW | DENV-4 | EDII | GGLS | ELISA | - | Non-neutralizing | 27707930, WO2013089647A1 |
| **741** | ANTI-DENV-10_50-6B-H1L2 | ARGGRAYYYDSTTPRDGESW | DENV-1 | EDII | GGLV | ELISA | - | Non-neutralizing | 27707930, WO2013089647A1 |
| **742** | ANTI-DENV-10_50-6D-H3L1 | ARAGPALYYDSSGYDTTPYDSFEV | DENV-3 | EDII | GGLL | NT50,DC-SIGN | - | Non-neutralizing | 23152560 |
| **743** | ANTI-DENV-10_50-6D-H3L1 | ARAGPALYYDSSGYDTTPYDSFEV | DENV-4 | EDII | GGLS | ELISA | - | Non-neutralizing | 23152560 |
| **744** | ANTI-DENV-10_50-6D-H3L1 | ARAGPALYYDSSGYDTTPYDSFEV | DENV-1 | EDII | GGLV | ELISA | - | Non-neutralizing | 23152560 |
| **745** | ANTI-DENV-10_50-6E-H1L1 | AGGGIVSTMGAFGF | DENV-4 | EDII | AAA | NT50 | - | Non-neutralizing | 27707930, WO2013089647A1 |
| **746** | ANTI-DENV-10_50-6E-H1L1 | AGGGIVSTMGAFGF | DENV-2 | EDII | WLFK | ELISA | - | Non-neutralizing | 27707930, WO2013089647A1 |
| **747** | ANTI-DENV-10_50-6E-H1L1 | AGGGIVSTMGAFGF | DENV-1 | EDII | WLFV | ELISA | - | Non-neutralizing | 27707930, WO2013089647A1 |
| **748** | ANTI-DENV-10_50-7C-H3L1 | ARGGWAYYYDSYTTPRDGQSWWFDP | DENV-1 | EDII | GWFS | ELISA | - | Non-neutralizing | 27707930, WO2013089647A1 |
| **749** | ANTI-DENV-10_50-7C-H3L1 | ARGGWAYYYDSYTTPRDGQSWWFDP | DENV-3 | EDII | GWFT | ELISA | - | Non-neutralizing | 27707930, WO2013089647A1 |
| **750** | ANTI-DENV-10_50-7C-H3L1 | ARGGWAYYYDSYTTPRDGQSWWFDP | DENV-4 | EDII | GWFV | ELISA | - | Non-neutralizing | 27707930, WO2013089647A1 |
| **751** | ANTI-DENV-10_50-7E-H1L1 | ARHDCSDTTCREYFQH | DENV-3 | Interdomain | RILALE | ELISA | - | Non-neutralizing | 27707930, WO2013089647A1 |
| **752** | ANTI-DENV-10_50-7E-H1L1 | ARHDCSDTTCREYFQH | DENV-3 | Interdomain | RILALM | ELISA | - | Non-neutralizing | 27707930, WO2013089647A1 |
| **753** | ANTI-DENV-10_50-7E-H1L1 | ARHDCSDTTCREYFQH | DENV-3 | Interdomain | RILALR | ELISA | - | Non-neutralizing | 27707930, WO2013089647A1 |
| **754** | ANTI-DENV-10_50-7H-H1L1 | AREGMVEVTALSFLDY | DENV-4 | Interdomain | RILALE | NT50,DC-SIGN | 70.00 | Non-neutralizing | 27707930, WO2013089647A1 |
| **755** | ANTI-DENV-10_50-9B-H2L2 | ATTNWGVSVKPGTFDI | DENV-3 | EDII | GGLL | ELISA | - | Non-neutralizing | 27707930, WO2013089647A1 |
| **756** | ANTI-DENV-10_50-9B-H2L2 | ATTNWGVSVKPGTFDI | DENV-4 | EDII | GGLS | ELISA | - | Non-neutralizing | 27707930, WO2013089647A1 |
| **757** | ANTI-DENV-10_50-9B-H2L2 | ATTNWGVSVKPGTFDI | DENV-1 | EDII | GGLV | ELISA | - | Non-neutralizing | 27707930, WO2013089647A1 |
| **758** | ANTI-DENV-10_50-9D-H1L1 | ARAGVALYYDSSGYDITPYDSFEI | DENV-3 | EDII | GGLL | NT50,DC-SIGN | - | Non-neutralizing | 27707930, WO2013089647A1 |
| **759** | ANTI-DENV-10_50-9D-H1L1 | ARAGVALYYDSSGYDITPYDSFEI | DENV-4 | EDII | GGLS | NT50,DC-SIGN | - | Non-neutralizing | 27707930, WO2013089647A1 |
| **760** | ANTI-DENV-10_50-9D-H1L1 | ARAGVALYYDSSGYDITPYDSFEI | DENV-1 | EDII | GGLV | NT50,DC-SIGN | >10 | Non-neutralizing | 27707930, WO2013089647A1 |
| **761** | ANTI-DENV-10_63-1E-H3L1 | ARGRGGFAATAGTDY | DENV-2 | EDII | GGLK | ELISA | - | Non-neutralizing | 27707930, WO2013089647A1 |
| **762** | ANTI-DENV-10_63-1E-H3L1 | ARGRGGFAATAGTDY | DENV-3 | EDII | GGLL | ELISA | - | Non-neutralizing | 27707930, WO2013089647A1 |
| **763** | ANTI-DENV-10_63-1E-H3L1 | ARGRGGFAATAGTDY | DENV-4 | EDII | GGLS | ELISA | - | Non-neutralizing | 27707930, WO2013089647A1 |
| **764** | ANTI-DENV-10_63-2A-H5L4 | VRDYTFVRGVASWEY | DENV-4 | EDII | GGLS | ELISA | - | Non-neutralizing | 27707930, WO2013089647A1 |
| **765** | ANTI-DENV-10_63-2A-H5L4 | VRDYTFVRGVASWEY | DENV-1 | EDII | GGLV | ELISA | - | Non-neutralizing | 27707930, WO2013089647A1 |
| **766** | ANTI-DENV-10_63-2C-H3L2 | SQRHILTGHYTDY | DENV-4 | EDIII | AAAL | ELISA | - | Non-neutralizing | 27707930, WO2013089647A1 |
| **767** | ANTI-DENV-10_63-2E-H2L2 | AKLQRPDSTVWPEDQ | DENV-3 | EDII | GGLL | ELISA | - | Non-neutralizing | 27707930, WO2013089647A1 |
| **768** | ANTI-DENV-10_63-2E-H2L2 | AKLQRPDSTVWPEDQ | DENV-4 | EDII | GGLS | ELISA | - | Non-neutralizing | 27707930, WO2013089647A1 |
| **769** | ANTI-DENV-10_63-2E-H2L2 | AKLQRPDSTVWPEDQ | DENV-1 | EDII | GGLV | ELISA | - | Non-neutralizing | 27707930, WO2013089647A1 |
| **770** | ANTI-DENV-10_63-2F-H1L1 | AEDTALYYCANVLGYCTPTTCYEDH | DENV-2 | EDII | AAA | ELISA | - | Non-neutralizing | 27707930, WO2013089647A1 |
| **771** | ANTI-DENV-10_63-2G-H2L1 | ARPDDSSGLEAFDI | DENV-3 | EDII | GGLL | ELISA | - | Non-neutralizing | 27707930, WO2013089647A1 |
| **772** | ANTI-DENV-10_63-2G-H2L1 | ARPDDSSGLEAFDI | DENV-4 | EDII | GGLS | ELISA | - | Non-neutralizing | 27707930, WO2013089647A1 |
| **773** | ANTI-DENV-10_63-2G-H2L1 | ARPDDSSGLEAFDI | DENV-1 | EDII | GGLV | ELISA | - | Non-neutralizing | 27707930, WO2013089647A1 |
| **774** | ANTI-DENV-10_63-3D-H2L2 | AREYCSSTTCINSFLTP | DENV-3 | EDII | GGLL | ELISA | - | Non-neutralizing | 27707930, WO2013089647A1 |
| **775** | ANTI-DENV-10_63-3D-H2L2 | AREYCSSTTCINSFLTP | DENV-4 | EDII | GGLS | ELISA | - | Non-neutralizing | 27707930, WO2013089647A1 |
| **776** | ANTI-DENV-10_63-3D-H2L2 | AREYCSSTTCINSFLTP | DENV-1 | EDII | GGLV | ELISA | - | Non-neutralizing | 27707930, WO2013089647A1 |
| **777** | ANTI-DENV-10_63-3E-H1L2 | VRDVSSYSIIWSDAFEI | DENV-4 | EDII | GGLS | ELISA | - | Non-neutralizing | 27707930, WO2013089647A1 |
| **778** | ANTI-DENV-10_63-3E-H1L2 | VRDVSSYSIIWSDAFEI | DENV-1 | EDII | GGLV | ELISA | - | Non-neutralizing | 27707930, WO2013089647A1 |
| **779** | ANTI-DENV-10_63-5A-H6L1 | ARCWRGYDPLTGYYTGYYFDY | DENV-4 | EDII | AAA | ELISA | - | Non-neutralizing | 27707930, WO2013089647A1 |
| **780** | ANTI-DENV-10_63-5B-H1L1 | ARHHSSGNALDY | DENV-1 | EDII | GGLV | NT50,DC-SIGN | >10 | Non-neutralizing | 27707930, WO2013089647A1 |
| **781** | ANTI-DENV-10_63-5C-H2L1 | AGAYCSATSCSSGQWLVY | DENV-1 | EDII | GWFS | ELISA | - | Non-neutralizing | 27707930, WO2013089647A1 |
| **782** | ANTI-DENV-10_63-5C-H2L1 | AGAYCSATSCSSGQWLVY | DENV-3 | EDII | GWFT | ELISA | - | Non-neutralizing | 27707930, WO2013089647A1 |
| **783** | ANTI-DENV-10_63-5C-H2L1 | AGAYCSATSCSSGQWLVY | DENV-4 | EDII | GWFV | ELISA | - | Non-neutralizing | 27707930, WO2013089647A1 |
| **784** | ANTI-DENV-10_63-6C-H8L1 | LTSEDMAVYFCARHHRHILTGERTDY | DENV-4 | EDIII | AAAAS | ELISA | - | Non-neutralizing | 27707930, WO2013089647A1 |
| **785** | ANTI-DENV-10_63-6E-H6L1 | ARGGHHILTGRYIDD | DENV-4 | EDII | GGLS | NT50,DC-SIGN | >10 | Non-neutralizing | 27707930, WO2013089647A1 |
| **786** | ANTI-DENV-10_63-6E-H6L1 | ARGGHHILTGRYIDD | DENV-1 | EDII | GGLV | NT50,DC-SIGN | >10 | Non-neutralizing | 27707930, WO2013089647A1 |
| **787** | B11F | AISLYYDISTGDNYYWYMDV | DENV-3 | Interdomain | TIQGI | ELISA | - | Non-neutralizing | 33597214 |
| **788** | B11F | AISLYYDISTGDNYYWYMDV | DENV-1 | Interdomain | SITTTS | ELISA | - | Non-neutralizing | 33597214 |
| **789** | B11F | AISLYYDISTGDNYYWYMDV | DENV-4 | Interdomain | TASTGI | ELISA | - | Non-neutralizing | 33597214 |
| **790** | C4 | ARYCSSPSCYHNWFDP | DENV-2 | Interdomain | KSTATNSI | ELISA | - | Non-neutralizing | 31820734, WO2020033491A1 |
| **791** | CR4354 | DWGSNYVWGSYPKY | DENV-3 | Interdomain | KEVTNQYLKGTTPQEILGTLKEK | NT50 | >10 | Non-neutralizing | 31820734, WO2020033491A1 |
| **792** | D29 | AHSSGWYDA | DENV-4 | Interdomain | KEVNIE | PRNT50,Vero | 20.00 | Non-neutralizing | 22509258 |
| **793** | D29 | AHSSGWYDA | DENV-1 | Interdomain | TNPKYE | PRNT50,Vero | 20.00 | Non-neutralizing | 22509258 |
| **794** | D29 | AHSSGWYDA | DENV-3 | Interdomain | TQLKHE | PRNT50,Vero | 20.00 | Non-neutralizing | 22509258 |
| **795** | D29 | AHSSGWYDA | DENV-4 | Interdomain | AKEVNLVQIEPWKR | PRNT50,Vero | 20.00 | Non-neutralizing | 22509258 |
| **796** | D29 | AHSSGWYDA | DENV-3 | Interdomain | ATQLKVVQHESWKK | PRNT50,Vero | 20.00 | Non-neutralizing | 22509258 |
| **797** | D29 | AHSSGWYDA | DENV-1 | Interdomain | VTNPKIVQYEPWKK | PRNT50,Vero | 20.00 | Non-neutralizing | 22509258 |
| **798** | DB25-2 | AKNFGTHYYGSNYGNFDY | DENV-3 | EDIII | EY | ELISA | - | Non-neutralizing | 26135599, US8637035B2 |
| **799** | DB32-6 | VRTGSFWYFDV | DENV-4 | EDIII | KEM | ELISA | - | Non-neutralizing | 26135599, US8637035B2 |
| **800** | DB32-6 | VRTGSFWYFDV | DENV-1 | EDIII | KEV | ELISA | - | Non-neutralizing | 26135599, US8637035B2 |
| **801** | DENV-1-E106 | RINWALDY | DENV-2 | EDIII | KKQYEDGKD | PRNT50,BHK21 | - | Non-neutralizing | 24743696 |
| **802** | DENV-1-E106 | RINWALDY | DENV-4 | EDIII | SKKYEAGTN | PRNT50,BHK21 | - | Non-neutralizing | 24743696 |
| **803** | DENV-1-E106 | RINWALDY | DENV-3 | EDIII | VKEYKEDKE | PRNT50,BHK21 | - | Non-neutralizing | 24743696 |
| **804** | DENV-1-E106 | RINWALDY | DENV-1 | EDIII | EGGGPEKPKE | PRNT50,BHK21 | - | Non-neutralizing | 20369024 |
| **805** | DENV-1-E111 | WFFPWYFDV | DENV-3 | EDIII | YMLKKPKEQPN | FCNT50 | - | Non-neutralizing | 20369024 |
| **806** | DENV-1-E111 | WFFPWYFDV | DENV-4 | EDIII | YMSDKPKRNVH | FCNT50 | - | Non-neutralizing | 20369024 |
| **807** | DENV-1-E111 | WFFPWYFDV | DENV-1 | EDIII | YVMCKIPFSSQDEKGITQNGRLIAGEAK | PRNT50,BHK21 | 25.00 | Non-neutralizing | 23055922 |
| **808** | DENV-1-E111 | WFFPWYFDV | DENV-1 | EDIII | YVMCKIPFSSQDEKGVTQNGRVVAGEAK | PRNT50,BHK21 | 25.00 | Non-neutralizing | 23055922 |
| **809** | DENV-1-E111 | WFFPWYFDV | DENV-1 | EDIII | YVMCKIPFSSQNEKGVTQNGRLVAGEAK | PRNT50,BHK21 | 15.20 | Non-neutralizing | 23055922 |
| **810** | DM25-3 | VRYGGYYVFDY | DENV-2 | Interdomain | VG | ELISA | - | Non-neutralizing | 30334522 |
| **811** | DM8-6 | ARSLLPNWYFD | DENV-4 | EDIII | DKE | FRNT50 | >10 | Non-neutralizing | 30334522 |
| **812** | DM8-6 | ARSLLPNWYFD | DENV-1 | EDIII | EKE | FRNT50 | >10 | Non-neutralizing | 30334522 |
| **813** | DM8-6 | ARSLLPNWYFD | DENV-3 | EDIII | KKE | FRNT50 | >10 | Non-neutralizing | 30334522 |
| **814** | DV22.3 | ARGPPTDCSSGRCLGVGVGLDP | DENV-2 | Interdomain | KGSHVNTNF | FCNT50,Vero | 20.00 | Non-neutralizing | WO2010043977 |
| **815** | DV22.3 | ARGPPTDCSSGRCLGVGVGLDP | DENV-3 | Interdomain | KGTHVNTNF | FCNT50,Vero | 20.00 | Non-neutralizing | WO2010043977 |
| **816** | DV22.3 | ARGPPTDCSSGRCLGVGVGLDP | DENV-1 | Interdomain | KGTHVNTTF | FCNT50,Vero | 20.00 | Non-neutralizing | WO2010043977 |
| **817** | DV87.1 | GGPRGLQLLSSWVDY | DENV-4 | EDIII | SELW | FCNT50,Vero | - | Non-neutralizing | 30185598 |
| **818** | EDE1-(2)A2 | ARGFYSGSYYPTAPFDI | DENV-2 | EDE | AAQAVNIAPTKEQAA | ELISA | - | Non-neutralizing | 25501631, WO2017212291 |
| **819** | EDE1-(2)A2 | ARGFYSGSYYPTAPFDI | DENV-4 | EDE | TSQWSNTAPKKKKWF | ELISA | - | Non-neutralizing | 25501631, WO2017212291 |
| **820** | EDE1-(2)A5 | ARVFYSGSYYPNSPFDY | DENV-4 | EDE | TQWSNTTAPKKKKKWF | ELISA | - | Non-neutralizing | 25501631, WO2017212291 |
| **821** | EDE1-(2)A7 | ARSLWSGELWGGPLGY | DENV-2 | EDE | AKAAVNAGSTKEIKLKQAA | ELISA | - | Non-neutralizing | 25501631, WO2017212291 |
| **822** | EDE1-(2)A8 | ARSYNWNDVFYYYYMDV | DENV-4 | EDE | TKSQWKNTAPKKKMKIKKWF | ELISA | - | Non-neutralizing | 25501631, WO2017212291 |
| **823** | EDE1-(2)B10 | ARVEGGPKYYFGSGDFYNL | DENV-4 | EDE | TSQWSNTAPKKKKKWF | ELISA | - | Non-neutralizing | 25501631, WO2017212291 |
| **824** | EDE1-(2)B11 | ARVFYSGSYYPNSPFDS | DENV-2 | EDE | AAAAGAAA | ELISA | - | Non-neutralizing | 25501631, WO2017212291 |
| **825** | EDE1-(2)C2 | ARGYNWNDVQYYYTMDV | DENV-4 | EDE | TSQWNTAPKKKIKKWF | ELISA | - | Non-neutralizing | 25501631, WO2017212291 |
| **826** | EDE1-(2)D4 | ARGYNWNDVHYYYTMDV | DENV-2 | EDE | EKQWNAAPTKAQAA | ELISA | - | Non-neutralizing | 25501631, WO2017212291 |
| **827** | EDE1-(3)B10 | ARPLAHTYDFWSGYHRATGYGMDV | DENV-4 | EDE | TSQWSNTAKKDIKWF | FCNT50,Vero | 100.00 | Non-neutralizing | 25501631, WO2017212291 |
| **828** | EDE1-(3)C10 | ARDKVDDYGDYWFPTLWYFDY | DENV-2 | EDE | EKQAVNIAPTKEKQAA | ELISA | - | Non-neutralizing | 25501631, WO2017212291 |
| **829** | EDE1-(3)C10-(AllHCDR3-A) | AAAAAAAAAAAAAAAAAAAAAA | DENV-1 | EDE | RHELNEVRWLHVKKK | FRNT50,Vero | >10 | Non-neutralizing | 34852239 |
| **830** | EDE1-(3)C10-(AllHCDR3-A) | AAAAAAAAAAAAAAAAAAAAAA | DENV-4 | EDE | RHETNEVRWHVKQDDMK | FRNT50,Vero | >10 | Non-neutralizing | 34852239 |
| **831** | EDE1-(3)C10-(AllHCDR3-A) | AAAAAAAAAAAAAAAAAAAAAA | DENV-3 | EDE | RHGELQNEVRWLIHVKKKQ | FRNT50,Vero | >10 | Non-neutralizing | 34852239 |
| **832** | EDE1-(3)C10-(D100B-A) | ARDKVDDYGAYWFPTLWYFDY | DENV-3 | EDE | RHGELQNEVRWLIHVKKKQ | FRNT50,Vero | >10 | Non-neutralizing | 34852239 |
| **833** | EDE1-(3)C10-(D98-A) | ARDKVADYGDYWFPTLWYFDY | DENV-3 | EDE | RHGELQNEVRWLIHVKKKQ | FRNT50,Vero | >10 | Non-neutralizing | 34852239 |
| **834** | EDE1-(3)C10-(D99-A) | ARDKVDAYGDYWFPTLWYFDY | DENV-3 | EDE | RHGELQNEVRWLIHVKKKQ | FRNT50,Vero | >10 | Non-neutralizing | 34852239 |
| **835** | EDE1-(3)C10-(L100H-A) | ARDKVDDYGDYWFPTAWYFDY | DENV-4 | EDE | RHETNEVRWHVKQDDMK | FRNT50,Vero | >10 | Non-neutralizing | 34852239 |
| **836** | EDE1-(3)C10-(L100H-A) | ARDKVDDYGDYWFPTAWYFDY | DENV-2 | EDE | RHGELINEVRWITHVKKQK | FRNT50,Vero | >10 | Non-neutralizing | 34852239 |
| **837** | EDE1-(3)C10-(L100H-A) | ARDKVDDYGDYWFPTAWYFDY | DENV-3 | EDE | RHGELQNEVRWLIHVKKKQ | FRNT50,Vero | >10 | Non-neutralizing | 34852239 |
| **838** | EDE1-(3)C10-(P100F-A) | ARDKVDDYGDYWFETLWYFDY | DENV-4 | EDE | RHETNEVRWHVKQDDMK | FRNT50,Vero | >10 | Non-neutralizing | 34852239 |
| **839** | EDE1-(3)C10-(P100F-A) | ARDKVDDYGDYWFETLWYFDY | DENV-3 | EDE | RHGELQNEVRWLIHVKKKQ | FRNT50,Vero | >10 | Non-neutralizing | 34852239 |
| **840** | EDE1-(3)C10-(Y100-A) | ARDKVDDAGDYWFPTLWYFDY | DENV-3 | EDE | RHGELQNEVRWLIHVKKKQ | FRNT50,Vero | >10 | Non-neutralizing | 34852239 |
| **841** | EDE1-(3)C10-(Y100C-A) | ARDKVDDYGDAWFPTLWYFDY | DENV-1 | EDE | RHELNEVRWLHVKKK | FRNT50,Vero | >10 | Non-neutralizing | 34852239 |
| **842** | EDE1-(3)C10-(Y100C-A) | ARDKVDDYGDAWFPTLWYFDY | DENV-4 | EDE | RHETNEVRWHVKQDDMK | FRNT50,Vero | >10 | Non-neutralizing | 34852239 |
| **843** | EDE1-(3)C10-(Y100C-A) | ARDKVDDYGDAWFPTLWYFDY | DENV-3 | EDE | RHGELQNEVRWLIHVKKKQ | FRNT50,Vero | >10 | Non-neutralizing | 34852239 |
| **844** | EDE1-(4)B3 | ARGRRAYDSSGYVKYYYFYGVDV | DENV-4 | EDE | TSQWSIAPKKKSKKWF | ELISA | - | Non-neutralizing | 25501631, WO2017212291 |
| **845** | EDE1-2A2 | VGYSSFYYYYTMDV | DENV-2 | EDE | AKAAVNIAPAKAIKQAA | ELISA | - | Non-neutralizing | 25501631, WO2017212291 |
| **846** | EDE1-2A4 | ARGLYSVSYYPTSPFDY | DENV-2 | EDE | AKAAVNAAPTKAGKKQAF | ELISA | - | Non-neutralizing | 25501631, WO2017212291 |
| **847** | EDE1-2A9 | VGYSSFYYYYTMDV | DENV-2 | EDE | AKQWNAGPAAGKLKQAA | ELISA | - | Non-neutralizing | 25501631, WO2017212291 |
| **848** | EDE1-2B11 | VGYSSFYYFYTVDV | DENV-2 | EDE | AKQANAGPAKAKQAA | ELISA | - | Non-neutralizing | 25501631, WO2017212291 |
| **849** | EDE1-2B2 | VGYSSFYYYYTMDV | DENV-2 | EDE | AKQANAGPAKEKRAA | ELISA | - | Non-neutralizing | 25501631, WO2017212291 |
| **850** | EDE1-2B3 | VGYSTFYYYYTMDV | DENV-2 | EDE | AKQAVNAGPAKAGLQAA | ELISA | - | Non-neutralizing | 25501631, WO2017212291 |
| **851** | EDE1-2B4 | VGYSSFYFYYTMDV | DENV-2 | EDE | AKQAVNAGPAKALAQAA | ELISA | - | Non-neutralizing | 25501631, WO2017212291 |
| **852** | EDE1-2B7 | ARGLYSGSHYPTSPLDY | DENV-4 | EDE | TQWNIAPKKKWF | ELISA | - | Non-neutralizing | 25501631, WO2017212291 |
| **853** | EDE1-2B8 | VGYSNFYYYYTMDV | DENV-1 | EDE | EQWNIAPTEQWF | FCNT50,Vero | - | Non-neutralizing | 25501631, WO2017212291 |
| **854** | EDE1-2C4 | ARGYNWNDVHYYYTMDV | DENV-2 | EDE | AKQAVNAGPAKELKQAA | ELISA | - | Non-neutralizing | 25501631, WO2017212291 |
| **855** | EDE1-2C4 | ARGYNWNDVHYYYTMDV | DENV-4 | EDE | TSQWSNTAPKKKIKKWF | ELISA | - | Non-neutralizing | 25501631, WO2017212291 |
| **856** | EDE1-2C8 | GYSNFYYYYTMDV | DENV-2 | EDE | AQWNAAPTERAA | ELISA | - | Non-neutralizing | 25501631, WO2017212291 |
| **857** | EDE1-A12 | ARVHTGGYPPELRYYYYGMDV | DENV-2 | EDE | AKQAVNAAPAKEQAA | ELISA | - | Non-neutralizing | 25501631, WO2017212291 |
| **858** | EDE1-B11 | VGGYSSFYYYYTLDV | DENV-2 | EDE | EKQANAAPAKEQAA | ELISA | - | Non-neutralizing | 25501631, WO2017212291 |
| **859** | EDE1-C9 | VGGYSSFYYYYTMDV | DENV-2 | EDE | AKQAVNIAPTKEKQAA | ELISA | - | Non-neutralizing | 25501631, WO2017212291 |
| **860** | EDE1-P6A1 | AKPAHYDDSGYPYMAYFDS | DENV-2 | EDE | EQWIAPTEQAA | ELISA | - | Non-neutralizing | 25501631, WO2017212291 |
| **861** | EDE1-P6A12 | AGRYCSSTSCSDPWTYFPH | DENV-2 | EDE | AKAAVNAAPAKEGILEQAA | ELISA | - | Non-neutralizing | 25501631, WO2017212291 |
| **862** | EDE1-P6A3 | ARRHCSSTSCSDPWTFFPS | DENV-2 | EDE | AQAVNIAPAKEQAA | ELISA | - | Non-neutralizing | 25501631, WO2017212291 |
| **863** | EDE1-P6B11 | AKEISYCGGDCQNFFFYYNMDV | DENV-2 | EDE | AKKQAVNAAPAKEGILEKQAA | ELISA | - | Non-neutralizing | 25501631, WO2017212291 |
| **864** | EDE1-P6B4 | ARWGGDCNAGSCYGPYQYRGLDA | DENV-2 | EDE | AKQAVAAGPTKEGILEQAA | ELISA | - | Non-neutralizing | 25501631, WO2017212291 |
| **865** | EDE1-P6B5 | AGRSDNWNDVYYNYALDV | DENV-2 | EDE | EKQAVNAAPTKEGILKQAA | ELISA | - | Non-neutralizing | 25501631, WO2017212291 |
| **866** | EDE1-P6C4 | ARDLGAMGYYLCSAGNCPFDY | DENV-2 | EDE | AAAIPAAF | ELISA | - | Non-neutralizing | 25501631, WO2017212291 |
| **867** | EDE2-(2)D2 | ARAHSGNYDFWSGSNYHYYYGMDV | DENV-2 | EDE | AKQAVNAAAAPTKEKQAA | Single-pointELISA | - | Non-neutralizing | 25501631, WO2017212291 |
| **868** | EDE2-(2)D2 | ARAHSGNYDFWSGSNYHYYYGMDV | DENV-3 | EDE | EKQWLNNTTASTKNKKWY | Single-pointELISA | - | Non-neutralizing | 25501631, WO2017212291 |
| **869** | EDE2-(2)D2 | ARAHSGNYDFWSGSNYHYYYGMDV | DENV-4 | EDE | TSQWSNNLTAPKKKKKWF | Single-pointELISA | - | Non-neutralizing | 25501631, WO2017212291 |
| **870** | EDE2-(4)A10 | VRDGVRYYYDSSGYYPDSFFKYGMDV | DENV-2 | EDE | AKQAVNNTIAPTKEKQAA | ELISA | - | Non-neutralizing | 25501631, WO2017212291 |
| **871** | EDE2-(4)A11 | VDGVRFYYDSTGYYPDSFFKYGMDV | DENV-2 | EDE | AKQAVNTAAPTKKAAA | ELISA | - | Non-neutralizing | 25501631, WO2017212291 |
| **872** | EDE2-(4)A3 | ARDGVRYYYDSTGYYPDSYYEYGMDV | DENV-2 | EDE | EKQAVAAAAPTKEKQAA | ELISA | - | Non-neutralizing | 25501631, WO2017212291 |
| **873** | EDE2-(4)B4 | ARATNYFDSSGYFFAPWFDP | DENV-2 | EDE | AKQAVNAAAAPTKEKKQAA | Single-pointELISA | >10 | Non-neutralizing | 25501631, WO2017212291 |
| **874** | EDE2-(4)B4 | ARATNYFDSSGYFFAPWFDP | DENV-4 | EDE | TSQWSNNLTAPKKKSKKWF | Single-pointELISA | >10 | Non-neutralizing | 25501631, WO2017212291 |
| **875** | EDE2-(4)B6 | ASGGGGYAGYNWFDP | DENV-2 | EDE | AKQAVAAAAPTKEKQAA | ELISA | - | Non-neutralizing | 25501631, WO2017212291 |
| **876** | EDE2-(4)B6 | ASGGGGYAGYNWFDP | DENV-3 | EDE | EKQWVNTIAPKWMKKWY | ELISA | - | Non-neutralizing | 25501631, WO2017212291 |
| **877** | EDE2-(4)B6 | ASGGGGYAGYNWFDP | DENV-4 | EDE | TTQWVNTIAPKKKKQWF | ELISA | - | Non-neutralizing | 25501631, WO2017212291 |
| **878** | EDE2-(4)B7 | ARDGVRYYYDSTGYYPDNFFQYGLDV | DENV-2 | EDE | AKQAVAAAAPTKEKQAA | FCNT50,Vero | >10 | Non-neutralizing | 25501631, WO2017212291 |
| **879** | EDE2-(4)B7 | ARDGVRYYYDSTGYYPDNFFQYGLDV | DENV-4 | EDE | TTQWVNTIAPKKKKQWF | FCNT50,Vero | 13.95 | Non-neutralizing | 25501631, WO2017212291 |
| **880** | EDE2-(4)D6 | ARDGVRFYSDSTGYYPDNYFPYGMDV | DENV-2 | EDE | AKQAVNNTAAPTKEKQAA | ELISA | - | Non-neutralizing | 25501631, WO2017212291 |
| **881** | EDE2-B2 | ARDVNFHDSSGYYRQGFFAP | DENV-2 | EDE | AKQAVAAAAPTKEKQAA | ELISA | - | Non-neutralizing | 25501631, WO2017212291 |
| **882** | EDE2-B2 | ARDVNFHDSSGYYRQGFFAP | DENV-4 | EDE | TSQWSNLTAPKKKKKWF | ELISA | - | Non-neutralizing | 25501631, WO2017212291 |
| **883** | EDE2-B6 | ARAHSGNYDFWSGSNYHYYYGMDV | DENV-2 | EDE | EQWKNTIASQKKWF | ELISA | - | Non-neutralizing | 25501631, WO2017212291 |
| **884** | EDE2-B6 | ARAHSGNYDFWSGSNYHYYYGMDV | DENV-3 | EDE | EQWLNTTASKKKWY | ELISA | - | Non-neutralizing | 25501631, WO2017212291 |
| **885** | EDE2-B6 | ARAHSGNYDFWSGSNYHYYYGMDV | DENV-4 | EDE | TQWVNTIAPTKQWF | ELISA | - | Non-neutralizing | 25501631, WO2017212291 |
| **886** | EDE2-B8 | VRDGVRYYYDSSGYYPDSFFKYGMDV | DENV-2 | EDE | EKQWVNNTAAPAKEGKKKQAA | ELISA | - | Non-neutralizing | 25501631, WO2017212291 |
| **887** | EDE2-C2 | TRDDGPYSGYDWPWASSMDV | DENV-4 | EDE | TSQWSNNLTAPKKKKWF | ELISA | - | Non-neutralizing | 25501631, WO2017212291 |
| **888** | EDE2-C4 | ARDGVRFYYDSTGYYPDPYFQYGLDV | DENV-2 | EDE | AQAKAAIASQKKWF | ELISA | - | Non-neutralizing | 25501631, WO2017212291 |
| **889** | EDE2-C7 | ARDIGHYYDSSGYFHYSFGMDV | DENV-2 | EDE | EKQWVAAAIAPTKKQWA | ELISA | - | Non-neutralizing | 25501631, WO2017212291 |
| **890** | EDE2-C7 | ARDIGHYYDSSGYFHYSFGMDV | DENV-4 | EDE | TSQWSNNLTAPKKKKWF | ELISA | - | Non-neutralizing | 25501631, WO2017212291 |
| **891** | EDE2-D11 | ARDHPTVINPTFVGSWFDP | DENV-2 | EDE | AKQAVNAAAAPTKKQAA | ELISA | - | Non-neutralizing | 25501631, WO2017212291 |
| **892** | EDE2-D11 | ARDHPTVINPTFVGSWFDP | DENV-3 | EDE | EKQWLNNTTASTKKKWY | ELISA | - | Non-neutralizing | 25501631, WO2017212291 |
| **893** | EDE2-D11 | ARDHPTVINPTFVGSWFDP | DENV-4 | EDE | TSQWSNNLTAPKKKKWF | ELISA | - | Non-neutralizing | 25501631, WO2017212291 |
| **894** | EDE2-D5 | ARDVNFYDSSGYYREGWFDS | DENV-4 | EDE | TSQWSNNLTAPKKKMKKWF | ELISA | - | Non-neutralizing | 25501631, WO2017212291 |
| **895** | EDE2-D8 | ARDGVRYYYDSTGYYPDSYYEYGLDV | DENV-2 | EDE | EKQWVNATAAPTKEKLKQAA | ELISA | - | Non-neutralizing | 25501631, WO2017212291 |
| **896** | G9E | VGGSSAYNGDNGWREAASLDD | DENV-4 | EDIII | RV | FRNT50 | >10 | Non-neutralizing | 30996133 |
| **897** | HMB-DV-1 | ARDPCSSTTCYFGYYAMDV | DENV-4 | EDIII | SKSKELW | NT50,DC-SIGN | 20.00 | Non-neutralizing | WO2010043977 |
| **898** | HMB-DV-10 | ARVAEFDYVWGSFDF | DENV-3 | EDIII | LTKE | FCNT50,Vero | 20.00 | Non-neutralizing | WO2010043977 |
| **899** | HMB-DV-10 | ARVAEFDYVWGSFDF | DENV-1 | EDIII | TSKE | FCNT50,Vero | 20.00 | Non-neutralizing | WO2010043977 |
| **900** | HMB-DV-11 | ARVAEFDYVWGSFDF | DENV-3 | EDIII | LTVKEIW | FCNT50,Vero | 20.00 | Non-neutralizing | WO2010043977 |
| **901** | HMB-DV-11 | ARVAEFDYVWGSFDF | DENV-1 | EDIII | TLKKELW | FCNT50,Vero | 20.00 | Non-neutralizing | WO2010043977 |
| **902** | HMB-DV-13 | SRVLWDSSSTGTFDS | DENV-4 | EDIII | SKSKELW | FCNT50,Vero | 20.00 | Non-neutralizing | WO2010043977 |
| **903** | HMB-DV-14 | ARAFAKNWFDP | DENV-3 | EDIII | LNV | FCNT50,Vero | 20.00 | Non-neutralizing | WO2010043977 |
| **904** | HMB-DV-14 | ARAFAKNWFDP | DENV-4 | EDIII | SGS | FCNT50,Vero | 20.00 | Non-neutralizing | WO2010043977 |
| **905** | HMB-DV-2 | AREWAARGGIVDY | DENV-4 | EDIII | SKSKELW | FCNT50,Vero | 20.00 | Non-neutralizing | WO2010043977 |
| **906** | HMB-DV-6 | AKGGPRGLQLLSSWVDY | DENV-4 | EDIII | SELW | FCNT50,Vero | 20.00 | Non-neutralizing | WO2010043977 |
| **907** | HMB-DV-7 | QQANSFPPT | DENV-4 | Interdomain | WE | FCNT50,Vero | 20.00 | Non-neutralizing | WO2010043977 |
| **908** | HMB-DV-9 | ARGGGYSRNWYSYQNYGLDV | DENV-1 | EDIII | AE | FCNT50,Vero | 20.00 | Non-neutralizing | WO2010043977 |
| **909** | HMB-DV-9 | ARGGGYSRNWYSYQNYGLDV | DENV-3 | EDIII | ID | FCNT50,Vero | 20.00 | Non-neutralizing | WO2010043977 |
| **910** | HMB-DV-9 | ARGGGYSRNWYSYQNYGLDV | DENV-4 | EDIII | VD | FCNT50,Vero | 20.00 | Non-neutralizing | WO2010043977 |
| **911** | J8 | ARSCESPSCYHNWFDP | DENV-2 | Interdomain | KGSHVHANS | NT50 | 18.00 | Non-neutralizing | 31820734, WO2020033491A1 |
| **912** | J8 | ARSCESPSCYHNWFDP | DENV-2 | Interdomain | KGSHVHTNF | NT50 | 18.00 | Non-neutralizing | 31820734, WO2020033491A1 |
| **913** | J8 | ARSCESPSCYHNWFDP | DENV-2 | Interdomain | KGSHVNANF | NT50 | 18.00 | Non-neutralizing | 31820734, WO2020033491A1 |
| **914** | J9 | ARYCYSASCYHNWFDP | DENV-2 | Interdomain | KGSHVHTNF | NT50 | 30.00 | Non-neutralizing | 31820734, WO2020033491A1 |
| **915** | J9 | ARYCYSASCYHNWFDP | DENV-2 | Interdomain | KGSHVNANF | NT50 | 30.00 | Non-neutralizing | 31820734, WO2020033491A1 |
| **916** | J9 | ARYCYSASCYHNWFDP | DENV-2 | Interdomain | KGSSTHAAF | NT50 | 30.00 | Non-neutralizing | 31820734, WO2020033491A1 |
| **917** | L8 | ARVYFDSGGYFDS | DENV-2 | EDII | YFAA | NT50 | >10 | Non-neutralizing | 31820734, WO2020033491A1 |
| **918** | M1 | ARYCSSASCYHKWFDP | DENV-2 | EDII | YPFAAA | NT50 | >10 | Non-neutralizing | 31820734, WO2020033491A1 |
| **919** | M360.6 | VRTPYNWNDGPRGALDI | DENV-4 | EDIII | CSGKFSIIVVIGV | RVP-NT50,DC-SIGN | 21.00 | Non-neutralizing | 31242272 |
| **920** | M360.6 | VRTPYNWNDGPRGALDI | DENV-1 | EDIII | CTGSFKLVVVVGA | RVP-NT50,DC-SIGN | 12.00 | Non-neutralizing | 31242272 |
| **921** | m366.6 | ARYMAGIWTFDI | DENV-4 | EDIII | CSGKFSIIVVIGV | PRNT50,BHK21 | 23.31 | Non-neutralizing | 31242272 |
| **922** | m366.6 | ARYMAGIWTFDI | DENV-1 | EDIII | CTGSFKLVVVVGA | PRNT50,BHK21 | 12.74 | Non-neutralizing | 31242272 |
| **923** | MZ2 | LDRYAWNDGGDHW | DENV-1 | Interdomain | KGL | FCNT50 | >10 | Non-neutralizing | 32015557 |
| **924** | MZ2 | LDRYAWNDGGDHW | DENV-4 | Interdomain | KGR | FCNT50 | >10 | Non-neutralizing | 32015557 |
| **925** | SMZAb2 | ARVRYYGSGTYYGGDAFDF | DENV-4 | EDII | WGLFS | ELISA | - | Non-neutralizing | 28978754 |
| **926** | WNE16 | SASYGDYADY | DENV-3 | EDIII | LNKE | ELISA | - | Non-neutralizing | 15852016 |
| **927** | WNE16 | SASYGDYADY | DENV-4 | EDIII | SGEA | ELISA | - | Non-neutralizing | 15852016 |
| **928** | WNE16 | SASYGDYADY | DENV-1 | EDIII | TGET | ELISA | - | Non-neutralizing | 15852016 |
| **929** | WNE16 | SASYGDYADY | DENV-3 | EDIII | YLNTFKGEDKEGPG | ELISA | - | Non-neutralizing | 16193056 |
| **930** | WNE16 | SASYGDYADY | DENV-4 | EDIII | YSGKFEGAGTNGDK | ELISA | - | Non-neutralizing | 16193056 |
| **931** | WNE16 | SASYGDYADY | DENV-1 | EDIII | YTGSFEGTDKEGEK | ELISA | - | Non-neutralizing | 16193056 |
| **932** | WNE53 | VYGNYPYFDV | DENV-2 | EDII | CPTQGEGCGLGK | ELISA | - | Non-neutralizing | 19713934 |
| **933** | WNV-86 | AKDRAYYFGSGTSGGAFDV | DENV-1 | EDII | AKNAQL | NT50 | >10 | Non-neutralizing | 30455471 |
| **934** | Z002 | AKDRGPRGVGELFDS | DENV-3 | EDIII | VMCTGSEGTDPKKIGDN | EC50 | - | Non-neutralizing | 28475892 |
| **935** | Z002 | AKDRGPRGVGELFDS | DENV-4 | EDIII | VMCTGSEGTDPQKVGNS | EC50 | - | Non-neutralizing | 28475892 |
| **936** | Z004-IGL | AKDRGPRGVGELFDY | DENV-3 | EDIII | VMCTGSEGTDPQKIGDN | SPR | - | Non-neutralizing | 32321830 |
| **937** | Z004-IGL | AKDRGPRGVGELFDY | DENV-4 | EDIII | VMCTGSEGTDPQKVGNS | SPR | - | Non-neutralizing | 32321830 |
| **938** | Z005 | AKDRLVRGFAEVLDY | DENV-4 | EDIII | VMCTGSEGTDPQKVGNS | EC50 | - | Non-neutralizing | 28475892 |
| **939** | Z006 | DRSNGWSSINL | DENV-3 | EDIII | YAMTNTGEDKGKAIGDNA | ELISA | - | Non-neutralizing | 28475892 |
| **940** | Z006 | DRSNGWSSINL | DENV-4 | EDIII | YTMSGKGAGKNKEVGNSA | ELISA | - | Non-neutralizing | 28475892 |
| **941** | Z006 | DRSNGWSSINL | DENV-1 | EDIII | YVMTGSGTDKKGVAGEKA | ELISA | - | Non-neutralizing | 28475892 |
| **942** | Z010 | VKDRGTGWSSIVH | DENV-3 | EDIII | VMCTGSEGTDPKKIGDN | EC50 | - | Non-neutralizing | 28475892 |
| **943** | Z012 | ARGKNHQTTVAVLSWYYGMDV | DENV-3 | EDIII | VMCTGSEGTDPKKIGDN | EC50 | - | Non-neutralizing | 28475892 |
| **944** | Z012 | ARGKNHQTTVAVLSWYYGMDV | DENV-1 | EDIII | VMCTGSEGTDPQKAGEK | EC50 | - | Non-neutralizing | 28475892 |
| **945** | Z012 | ARGKNHQTTVAVLSWYYGMDV | DENV-4 | EDIII | VMCTGSEGTDPQKVGNS | EC50 | - | Non-neutralizing | 28475892 |
| **946** | Z013 | AKDRVAFDGFHV | DENV-4 | EDIII | VMCTGSEGTDPQKVGNS | EC50 | - | Non-neutralizing | 28475892 |
| **947** | Z014 | AKDRLVRGFGEVLDS | DENV-4 | EDIII | VMCTGSEGTDPQKVGNS | EC50 | - | Non-neutralizing | 28475892 |
| **948** | Z016 | ARTLVAPSAQSMYYFDF | DENV-4 | EDIII | VMCTGSEGTDPQKVGNS | EC50 | - | Non-neutralizing | 28475892 |
| **949** | Z017 | ARGGRINAPLGFDP | DENV-3 | EDIII | VMCTGSEGTDPKKIGDN | EC50 | - | Non-neutralizing | 28475892 |
| **950** | Z017 | ARGGRINAPLGFDP | DENV-1 | EDIII | VMCTGSEGTDPQKAGEK | EC50 | - | Non-neutralizing | 28475892 |
| **951** | Z017 | ARGGRINAPLGFDP | DENV-4 | EDIII | VMCTGSEGTDPQKVGNS | EC50 | - | Non-neutralizing | 28475892 |
| **952** | Z018 | ARGGRINSPLGFDP | DENV-3 | EDIII | VMCTGSEGTDPKKIGDN | EC50 | - | Non-neutralizing | 28475892 |
| **953** | Z018 | ARGGRINSPLGFDP | DENV-1 | EDIII | VMCTGSEGTDPQKAGEK | EC50 | - | Non-neutralizing | 28475892 |
| **954** | Z018 | ARGGRINSPLGFDP | DENV-4 | EDIII | VMCTGSEGTDPQKVGNS | EC50 | - | Non-neutralizing | 28475892 |
| **955** | Z021 | NQPGGRAFDY | DENV-3 | EDIII | SYAMTNYGEDAPKEIGDN | EC50 | - | Non-neutralizing | 28475892 |
| **956** | Z021 | NQPGGRAFDY | DENV-2 | EDIII | SYSMTGYGDGSPKDVEPG | EC50 | - | Non-neutralizing | 28475892 |
| **957** | Z021 | NQPGGRAFDY | DENV-4 | EDIII | SYTMSGYGAGAPKNVGNS | EC50 | - | Non-neutralizing | 28475892 |
| **958** | Z024 | ARHDGRGYCSPTRCFFSGMDV | DENV-4 | EDIII | VMCTGSEGTDPQKVGNS | EC50 | - | Non-neutralizing | 28475892 |
| **959** | Z028 | TRSLVTPAAQSVQYFDS | DENV-3 | EDIII | VMCTGSEGTDPKKIGDN | RVP-NT50 | - | Non-neutralizing | 28475892 |
| **960** | Z031 | AKDRLNGGFGELFAS | DENV-4 | EDIII | VMCTGSEGTDPQKVGNS | RVP-NT50 | - | Non-neutralizing | 28475892 |
| **961** | Z034 | VRDRIQGGFGELYRY | DENV-3 | EDIII | VMCTGSEGTDPKKIGDN | RVP-NT50 | - | Non-neutralizing | 28475892 |
| **962** | Z035 | AKDRLSGGFGELFQK | DENV-4 | EDIII | VMCTGSEGTDPQKVGNS | RVP-NT50 | - | Non-neutralizing | 28475892 |
| **963** | Z036 | AKDRIPHGLGELYAN | DENV-1 | EDIII | VMCTGSEGTDPQKAGEK | RVP-NT50 | - | Non-neutralizing | 28475892 |
| **964** | Z036 | AKDRIPHGLGELYAN | DENV-4 | EDIII | VMCTGSEGTDPQKVGNS | RVP-NT50 | - | Non-neutralizing | 28475892 |
| **965** | Z037 | ARDARSRSWDRTGFFGP | DENV-3 | EDIII | VMCTGSEGTDPKKIGDN | RVP-NT50 | - | Non-neutralizing | 28475892 |
| **966** | Z037 | ARDARSRSWDRTGFFGP | DENV-1 | EDIII | VMCTGSEGTDPQKAGEK | RVP-NT50 | - | Non-neutralizing | 28475892 |
| **967** | Z037 | ARDARSRSWDRTGFFGP | DENV-4 | EDIII | VMCTGSEGTDPQKVGNS | RVP-NT50 | - | Non-neutralizing | 28475892 |
| **968** | Z039 | AKSPYVGGYGLPGDS | DENV-4 | EDIII | VMCTGSEGTDPQKVGNS | RVP-NT50 | - | Non-neutralizing | 28475892 |
| **969** | Z040 | AKSSGGHNWNYVDYYYGMDV | DENV-4 | EDIII | VMCTGSEGTDPQKVGNS | RVP-NT50 | - | Non-neutralizing | 28475892 |
| **970** | Z041 | ATPDWQYSSAYSLDH | DENV-4 | EDIII | VMCTGSEGTDPQKVGNS | RVP-NT50 | - | Non-neutralizing | 28475892 |
| **971** | Z042 | ARGPDNRY | DENV-3 | EDIII | VMCTGSEGTDPKKIGDN | RVP-NT50 | - | Non-neutralizing | 28475892 |
| **972** | Z042 | ARGPDNRY | DENV-1 | EDIII | VMCTGSEGTDPQKAGEK | RVP-NT50 | - | Non-neutralizing | 28475892 |
| **973** | Z042 | ARGPDNRY | DENV-4 | EDIII | VMCTGSEGTDPQKVGNS | RVP-NT50 | - | Non-neutralizing | 28475892 |
| **974** | Z043 | GRDSKGWLQLRGDIDY | DENV-4 | EDIII | VMCTGSEGTDPQKVGNS | RVP-NT50 | - | Non-neutralizing | 28475892 |
| **975** | Z044 | ARDFGGYSSSSVSDAFDI | DENV-3 | EDIII | VMCTGSEGTDPKKIGDN | RVP-NT50 | - | Non-neutralizing | 28475892 |
| **976** | Z044 | ARDFGGYSSSSVSDAFDI | DENV-1 | EDIII | VMCTGSEGTDPQKAGEK | RVP-NT50 | - | Non-neutralizing | 28475892 |
| **977** | Z044 | ARDFGGYSSSSVSDAFDI | DENV-4 | EDIII | VMCTGSEGTDPQKVGNS | RVP-NT50 | - | Non-neutralizing | 28475892 |
| **978** | Z050 | ARFRYYYESGGYSDASPYYLDY | DENV-3 | EDIII | VMCTGSEGTDPKKIGDN | RVP-NT50 | - | Non-neutralizing | 28475892 |
| **979** | Z050 | ARFRYYYESGGYSDASPYYLDY | DENV-1 | EDIII | VMCTGSEGTDPQKAGEK | RVP-NT50 | - | Non-neutralizing | 28475892 |
| **980** | Z050 | ARFRYYYESGGYSDASPYYLDY | DENV-4 | EDIII | VMCTGSEGTDPQKVGNS | RVP-NT50 | - | Non-neutralizing | 28475892 |
| **981** | Z051 | ARAWCEYAAYCWFDP | DENV-4 | EDIII | VMCTGSEGTDPQKVGNS | RVP-NT50 | - | Non-neutralizing | 28475892 |
| **982** | Z052 | ARAGFDYGSPVSAFDI | DENV-4 | EDIII | VMCTGSEGTDPQKVGNS | RVP-NT50 | - | Non-neutralizing | 28475892 |
| **983** | Z055 | ARRTYYDTRFPYWYFDL | DENV-4 | EDIII | VMCTGSEGTDPQKVGNS | RVP-NT50 | - | Non-neutralizing | 28475892 |
| **984** | Z056 | ARGGMLGQLWALDN | DENV-3 | EDIII | VMCTGSEGTDPKKIGDN | RVP-NT50 | - | Non-neutralizing | 28475892 |
| **985** | Z056 | ARGGMLGQLWALDN | DENV-1 | EDIII | VMCTGSEGTDPQKAGEK | RVP-NT50 | - | Non-neutralizing | 28475892 |
| **986** | Z056 | ARGGMLGQLWALDN | DENV-4 | EDIII | VMCTGSEGTDPQKVGNS | RVP-NT50 | - | Non-neutralizing | 28475892 |
| **987** | Z057 | ARGGRINVAEALRY | DENV-3 | EDIII | VMCTGSEGTDPKKIGDN | RVP-NT50 | - | Non-neutralizing | 28475892 |
| **988** | Z057 | ARGGRINVAEALRY | DENV-1 | EDIII | VMCTGSEGTDPQKAGEK | RVP-NT50 | - | Non-neutralizing | 28475892 |
| **989** | Z057 | ARGGRINVAEALRY | DENV-4 | EDIII | VMCTGSEGTDPQKVGNS | RVP-NT50 | - | Non-neutralizing | 28475892 |
| **990** | Z059 | ARGRDSSGRLLDH | DENV-3 | EDIII | VMCTGSEGTDPKKIGDN | RVP-NT50 | - | Non-neutralizing | 28475892 |
| **991** | Z059 | ARGRDSSGRLLDH | DENV-1 | EDIII | VMCTGSEGTDPQKAGEK | RVP-NT50 | - | Non-neutralizing | 28475892 |
| **992** | Z059 | ARGRDSSGRLLDH | DENV-4 | EDIII | VMCTGSEGTDPQKVGNS | RVP-NT50 | - | Non-neutralizing | 28475892 |
| **993** | Z061 | ARGEVGYFDL | DENV-3 | EDIII | VMCTGSEGTDPKKIGDN | RVP-NT50 | - | Non-neutralizing | 28475892 |
| **994** | Z061 | ARGEVGYFDL | DENV-1 | EDIII | VMCTGSEGTDPQKAGEK | RVP-NT50 | - | Non-neutralizing | 28475892 |
| **995** | Z061 | ARGEVGYFDL | DENV-4 | EDIII | VMCTGSEGTDPQKVGNS | RVP-NT50 | - | Non-neutralizing | 28475892 |
| **996** | Z062 | AREVDGIYGYLHY | DENV-3 | EDIII | VMCTGSEGTDPKKIGDN | RVP-NT50 | - | Non-neutralizing | 28475892 |
| **997** | Z062 | AREVDGIYGYLHY | DENV-1 | EDIII | VMCTGSEGTDPQKAGEK | RVP-NT50 | - | Non-neutralizing | 28475892 |
| **998** | Z062 | AREVDGIYGYLHY | DENV-4 | EDIII | VMCTGSEGTDPQKVGNS | RVP-NT50 | - | Non-neutralizing | 28475892 |
| **999** | Z10 | TRRDYRYDGGFDF | DENV-1 | EDII | FVDRGWG | ELISA | - | Non-neutralizing | 27974667 |
| **1000** | Z10 | TRRDYRYDGGFDF | DENV-4 | EDII | VVDRGWG | ELISA | - | Non-neutralizing | 27974667 |
| **1001** | Z10 | TRRDYRYDGGFDF | DENV-3 | EDII | YVDRGWG | ELISA | - | Non-neutralizing | 27974667 |
| **1002** | Z20 | ARDRDDLSPFDY | DENV-3 | EDII | DNGLF | ELISA | - | Non-neutralizing | 34267374 |
| **1003** | Z20 | ARDRDDLSPFDY | DENV-1 | EDII | KISNTTEDNFKFKETQKKVQTSG | FCNT50 | >10 | Non-neutralizing | 27974667 |
| **1004** | Z20 | ARDRDDLSPFDY | DENV-3 | EDII | KITNITEDNYKFQNETKKVQNSG | FCNT50 | >10 | Non-neutralizing | 27974667 |
| **1005** | Z20 | ARDRDDLSPFDY | DENV-4 | EDII | SISNITEDQYKFLKSEKKVDSGD | FCNT50 | >10 | Non-neutralizing | 27974667 |
| **1006** | Z23 | ARTGIAAAGFVFDY | DENV-4 | EDIII | MMDKLK | FCNT50,Vero | - | Non-neutralizing | 34267374 |
| **1007** | Z23 | ARTGIAAAGFVFDY | DENV-1 | EDIII | VLEKIT | FCNT50,Vero | - | Non-neutralizing | 34267374 |
| **1008** | Z23 | ARTGIAAAGFVFDY | DENV-4 | EDIII | MMDSLT | ELISA | - | Non-neutralizing | 27974667 |
| **1009** | Z24 | ARQQGNFFDH | DENV-1 | EDII | FVDRGWG | ELISA | - | Non-neutralizing | 27974667 |
| **1010** | Z24 | ARQQGNFFDH | DENV-4 | EDII | VVDRGWG | ELISA | - | Non-neutralizing | 27974667 |
| **1011** | Z24 | ARQQGNFFDH | DENV-3 | EDII | YVDRGWG | ELISA | - | Non-neutralizing | 27974667 |
| **1012** | Z25 | ARHDHPPLYGSGAIDY | DENV-1 | EDII | FVDRGWG | ELISA | - | Non-neutralizing | 27974667 |
| **1013** | Z25 | ARHDHPPLYGSGAIDY | DENV-4 | EDII | VVDRGWG | ELISA | - | Non-neutralizing | 27974667 |
| **1014** | Z25 | ARHDHPPLYGSGAIDY | DENV-3 | EDII | YVDRGWG | ELISA | - | Non-neutralizing | 27974667 |
| **1015** | Z30 | ARTTYSSTSGSFSINWFDP | DENV-3 | EDIII | MMDKLK | ELISA | - | Non-neutralizing | 27974667 |
| **1016** | Z30 | ARTTYSSTSGSFSINWFDP | DENV-4 | EDIII | MMDSLT | ELISA | - | Non-neutralizing | 27974667 |
| **1017** | Z30 | ARTTYSSTSGSFSINWFDP | DENV-1 | EDIII | MMEKLK | ELISA | - | Non-neutralizing | 27974667 |
| **1018** | Z3L1 | ARDHLGWSSTSGSFSINWFDP | DENV-3 | EDII | DWNGLF | ELISA | - | Non-neutralizing | 34267374 |
| **1019** | Z3L1 | ARDHLGWSSTSGSFSINWFDP | DENV-1 | Interdomain | LKENKSITHEKGETQKKVQTSG | ELISA | - | Non-neutralizing | 27974667 |
| **1020** | Z3L1 | ARDHLGWSSTSGSFSINWFDP | DENV-3 | Interdomain | QKEQKTIQVEKTNETKKVQNSG | ELISA | - | Non-neutralizing | 27974667 |
| **1021** | Z3L1 | ARDHLGWSSTSGSFSINWFDP | DENV-4 | Interdomain | TKTEETASHEKGKSEKKVDSGD | ELISA | - | Non-neutralizing | 27974667 |
| **1022** | Z6 | ARQWGNYFDH | DENV-2 | EDII | PTSWCGLFGK | NT50 | - | Non-neutralizing | 34267374 |
| **1023** | Z6 | ARQWGNYFDH | DENV-1 | EDII | PTTWCGLFGK | NT50 | - | Non-neutralizing | 34267374 |
| **1024** | Z6 | ARQWGNYFDH | DENV-3 | EDII | PTVWCGLFGK | NT50 | - | Non-neutralizing | 34267374 |
| **1025** | Z6 | ARQWGNYFDH | DENV-1 | EDII | FVDRGWG | ELISA | - | Non-neutralizing | 27974667 |
| **1026** | Z6 | ARQWGNYFDH | DENV-2 | EDII | MVDRGWG | ELISA | - | Non-neutralizing | 27974667 |
| **1027** | Z6 | ARQWGNYFDH | DENV-4 | EDII | VVDRGWG | ELISA | - | Non-neutralizing | 27974667 |
| **1028** | Z6 | ARQWGNYFDH | DENV-3 | EDII | YVDRGWG | ELISA | - | Non-neutralizing | 27974667 |
| **1029** | Z7 | ARHPRPETGYDFDY | DENV-1 | EDII | FVDRGWG | ELISA | - | Non-neutralizing | 27974667 |
| **1030** | Z7 | ARHPRPETGYDFDY | DENV-4 | EDII | VVDRGWG | ELISA | - | Non-neutralizing | 27974667 |
| **1031** | Z7 | ARHPRPETGYDFDY | DENV-3 | EDII | YVDRGWG | ELISA | - | Non-neutralizing | 27974667 |
| **1032** | ZIKV-116 | DRLSRGVGELYDS | DENV-3 | EDIII | AMLNDGKEGDK | ELISA | - | Non-neutralizing | 31757867 |
| **1033** | ZIKV-116 | DRLSRGVGELYDS | DENV-2 | EDIII | SMTGGEKRVEPG | ELISA | - | Non-neutralizing | 31757867 |
| **1034** | ZIKV-116 | DRLSRGVGELYDS | DENV-4 | EDIII | TMSGGNNDLGDS | ELISA | - | Non-neutralizing | 31757867 |
| **1035** | ZIKV-116 | DRLSRGVGELYDS | DENV-3 | EDIII | LDK | FC | - | Non-neutralizing | 27819683 |
| **1036** | ZIKV-116 | DRLSRGVGELYDS | DENV-4 | EDIII | SDS | FC | - | Non-neutralizing | 27819683 |
| **1037** | ZIKV-116 | DRLSRGVGELYDS | DENV-2 | EDIII | TPG | FC | - | Non-neutralizing | 27819683 |
| **1038** | ZIKV-117 | ARENYGSVY | DENV-3 | EDII | NNK | FC | - | Non-neutralizing | 27819683 |
| **1039** | ZIKV-117 | ARENYGSVY | DENV-4 | EDII | NQK | FC | - | Non-neutralizing | 27819683 |
| **1040** | ZK12-2 | ARQSASTGTMAVGSFDY | DENV-1 | Interdomain | KGTHVNTTF | PRNT50,Vero | 12.59 | Non-neutralizing | 28614803 |
| **1041** | ZK2B10 | ARVRSGTNYGSYYYYYGMDV | DENV-1 | EDIII | TGSVKYEGTENEK | PRNT50,Vero | 40.00 | Non-neutralizing | 28614803 |
| **1042** | ZK2B10 | ARVRSGTNYGSYYYYYGMDV | DENV-3 | EDIII | LNTVEYKGEENDK | EC50 | - | Non-neutralizing | 31708914 |
| **1043** | ZK2B10 | ARVRSGTNYGSYYYYYGMDV | DENV-4 | EDIII | SGKVKYEGAENES | EC50 | - | Non-neutralizing | 31708914 |
| **1044** | ZK6F5 | ARSGYSSAARGGWFDP | DENV-1 | Interdomain | KGTHVNTTF | PRNT50,Vero | 40.00 | Non-neutralizing | 28614803 |
| **1045** | ZK7C3 | AKCQQFVGDDAFDI | DENV-2 | Interdomain | KGSHVNTNF | PRNT50,Vero | 40.00 | Non-neutralizing | 28614803 |
| **1046** | ZK7C3 | AKCQQFVGDDAFDI | DENV-1 | Interdomain | KGTHVNTTF | PRNT50,Vero | 40.00 | Non-neutralizing | 28614803 |
| **1047** | ZKA117 | ARLNDGSTVTTSSYFDY | DENV-4 | EDII | NQK | ELISA | - | Non-neutralizing | 27417494 |
| **1048** | ZKA117 | ARLNDGSTVTTSSYFDY | DENV-2 | EDII | NRM | ELISA | - | Non-neutralizing | 27417494 |
| **1049** | ZKA134 | ARSDIVSTTRGYHHYGMDV | DENV-4 | EDIII | YEA | EC50 | - | Non-neutralizing | 27417494 |
| **1050** | ZKA134 | ARSDIVSTTRGYHHYGMDV | DENV-3 | EDIII | YED | EC50 | - | Non-neutralizing | 27417494 |
| **1051** | ZKA134 | ARSDIVSTTRGYHHYGMDV | DENV-1 | EDIII | YET | EC50 | - | Non-neutralizing | 27417494 |
| **1052** | ZKA185 | ARRYCSSSSCYVDN | DENV-1 | EDII | NK | EC50 | - | Non-neutralizing | 31511387 |
| **1053** | ZKA190 | AKSGTQYYDTTGYEYRGLEYFGY | DENV-4 | EDIII | YEA | EC50 | - | Non-neutralizing | 28938115 |
| **1054** | ZKA190 | AKSGTQYYDTTGYEYRGLEYFGY | DENV-3 | EDIII | YED | EC50 | - | Non-neutralizing | 28938115 |
| **1055** | ZKA190 | AKSGTQYYDTTGYEYRGLEYFGY | DENV-1 | EDIII | YET | EC50 | - | Non-neutralizing | 28938115 |
| **1056** | ZKA3 | VKDLAVLESDRLEVDQ | DENV-4 | Interdomain | KGSHVNTVF | EC50 | - | Non-neutralizing | 27417494 |
| **1057** | ZKA3 | VKDLAVLESDRLEVDQ | DENV-3 | Interdomain | KGTHVNTNF | EC50 | - | Non-neutralizing | 27417494 |
| **1058** | ZKA4 | ARGPVPYWSGESYSGAYFDF | DENV-2 | Interdomain | KGSHVNTNF | EC50 | - | Non-neutralizing | 27417494 |
| **1059** | ZKA4 | ARGPVPYWSGESYSGAYFDF | DENV-4 | Interdomain | KGSHVNTVF | EC50 | - | Non-neutralizing | 27417494 |
| **1060** | ZKA4 | ARGPVPYWSGESYSGAYFDF | DENV-3 | Interdomain | KGTHVNTNF | EC50 | - | Non-neutralizing | 27417494 |
| **1061** | ZKA5 | ARSGIETVAGSIDYYGMDV | DENV-4 | Interdomain | KGSHVNTVF | EC50 | - | Non-neutralizing | 27417494 |
| **1062** | ZKA5 | ARSGIETVAGSIDYYGMDV | DENV-3 | Interdomain | KGTHVNTNF | EC50 | - | Non-neutralizing | 27417494 |
| **1063** | ZKA5 | ARSGIETVAGSIDYYGMDV | DENV-1 | Interdomain | KGTHVNTTF | NT50,Vero | >10 | Non-neutralizing | 27417494 |
| **1064** | ZKA6 | ATGVTMFQGAQTNAEYLHY | DENV-4 | Interdomain | KGSHVNTVF | EC50 | - | Non-neutralizing | 27417494 |
| **1065** | ZKA6 | ATGVTMFQGAQTNAEYLHY | DENV-3 | Interdomain | KGTHVNTNF | EC50 | - | Non-neutralizing | 27417494 |
| **1066** | ZKA6 | ATGVTMFQGAQTNAEYLHY | DENV-1 | Interdomain | KGTHVNTTF | NT50,Vero | >10 | Non-neutralizing | 27417494 |
| **1067** | ZKA64 | ARMSSSIWGFDH | DENV-4 | EDIII | DEA | EC50 | - | Non-neutralizing | 31511387 |
| **1068** | ZKA64 | ARMSSSIWGFDH | DENV-1 | EDIII | EET | EC50 | - | Non-neutralizing | 31511387 |
| **1069** | ZKA64 | ARMSSSIWGFDH | DENV-3 | EDIII | VED | EC50 | - | Non-neutralizing | 31511387 |
| **1070** | ZKA7 | VKDFWSGDQSLESDF | DENV-4 | Interdomain | KGSHVNTVF | EC50 |  | Non-neutralizing | 27417494 |
| **1071** | ZKA7 | VKDFWSGDQSLESDF | DENV-3 | Interdomain | KGTHVNTNF | EC50 | - | Non-neutralizing | 27417494 |
| **1072** | ZKA78 | VKDLAIPESYRIEADY | DENV-4 | Interdomain | KGSHVNTVF | EC50 | - | Non-neutralizing | 27417494 |
| **1073** | ZKA78 | VKDLAIPESYRIEADY | DENV-3 | Interdomain | KGTHVNTNF | EC50 | - | Non-neutralizing | 27417494 |
| **1074** | ZKA8 | VKDFGTSPQTDF | DENV-4 | Interdomain | KGSHVNTVF | EC50 | - | Non-neutralizing | 27417494 |
| **1075** | ZKA8 | VKDFGTSPQTDF | DENV-3 | Interdomain | KGTHVNTNF | EC50 | - | Non-neutralizing | 27417494 |
| **1076** | ZV-2 | YYYGSYYALDY | DENV-4 | EDIII | KDKIMAETQVKTFAENSVTNE | ELISA | - | Non-neutralizing | 27475895, WO2018022786A1 |
| **1077** | ZV-2 | YYYGSYYALDY | DENV-1 | EDIII | KEKIVAETQLQNIVTDKPVNE | ELISA | - | Non-neutralizing | 27475895, WO2018022786A1 |
| **1078** | ZV-2 | YYYGSYYALDY | DENV-3 | EDIII | KKKIVSETQLKNVVTKEPVNE | ELISA | - | Non-neutralizing | 27475895, WO2018022786A1 |
| **1079** | ZV-48 | LGNDMDY | DENV-1 | EDIII | MKPFSDEGVTQNEY | ELISA | - | Non-neutralizing | 27475895, WO2018022786A1 |
| **1080** | ZV-48 | LGNDMDY | DENV-3 | EDIII | MKPFSDGGKAHNEN | ELISA | - | Non-neutralizing | 27475895, WO2018022786A1 |
| **1081** | ZV-48 | LGNDMDY | DENV-4 | EDIII | MKPIEDVKRKVVDY | ELISA | - | Non-neutralizing | 27475895, WO2018022786A1 |
| **1082** | ZV-54 | ENYGSVY | DENV-4 | EDIII | KEATNAS | ELISA | - | Non-neutralizing | 27475895, WO2018022786A1 |
| **1083** | ZV-54 | ENYGSVY | DENV-1 | EDIII | SETKEEK | ELISA | - | Non-neutralizing | 27475895, WO2018022786A1 |
| **1084** | ZV-54 | ENYGSVY | DENV-3 | EDIII | TKEKEDK | ELISA | - | Non-neutralizing | 27475895, WO2018022786A1 |
| **1085** | ZV-64 | YYYDYDGMDY | DENV-1 | EDIII | MKPFSDEGVTQNLA | ELISA | - | Non-neutralizing | 27475895, WO2018022786A1 |
| **1086** | ZV-64 | YYYDYDGMDY | DENV-3 | EDIII | MKPFSDGGKAHNLI | ELISA | - | Non-neutralizing | 27475895, WO2018022786A1 |
| **1087** | ZV-64 | YYYDYDGMDY | DENV-4 | EDIII | MKPIEDVKEKVVIV | ELISA | - | Non-neutralizing | 27475895, WO2018022786A1 |
| **1088** | ZV-67 | ENYGSVY | DENV-3 | EDIII | LNTFVLEYKGEDAKEDKALK | ELISA | - | Non-neutralizing | 27475895, WO2018022786A1 |
| **1089** | ZV-67 | ENYGSVY | DENV-4 | EDIII | SGKFSIKYEGAGATNASALT | ELISA | - | Non-neutralizing | 27475895, WO2018022786A1 |
| **1090** | ZV-67 | ENYGSVY | DENV-1 | EDIII | TGSFKLKYEGTDGKEEKALK | ELISA | - | Non-neutralizing | 27475895, WO2018022786A1 |
| **1091** | ZWT.1 | ARGGNDYSMDY | DENV-2 | EDII | PTSWCGLFGK | ELISA | - | Non-neutralizing | 34267374 |
| **1092** | ZWT.1 | ARGGNDYSMDY | DENV-3 | EDII | PTVWCGLFGK | ELISA | - | Non-neutralizing | 34267374 |
| **1093** | ZWT.1 | ARGGNDYSMDY | DENV-4 | EDII | PTYWCGLFGK | ELISA | - | Non-neutralizing | 34267374 |
| **1094** | ZWT.2 | ARGGDSYSMDY | DENV-2 | EDII | PTSWCGLFGK | ELISA | - | Non-neutralizing | 34267374 |
| **1095** | ZWT.2 | ARGGDSYSMDY | DENV-3 | EDII | PTVWCGLFGK | ELISA | - | Non-neutralizing | 34267374 |
| **1096** | ZWT.2 | ARGGDSYSMDY | DENV-4 | EDII | PTYWCGLFGK | ELISA | - | Non-neutralizing | 34267374 |
| **1097** | ZWT.3 | ARGGDDYGMDY | DENV-2 | EDII | PTSWCGLFGK | ELISA | - | Non-neutralizing | 34267374 |
| **1098** | ZWT.3 | ARGGDDYGMDY | DENV-3 | EDII | PTVWCGLFGK | ELISA | - | Non-neutralizing | 34267374 |
| **1099** | ZWT.3 | ARGGDDYGMDY | DENV-4 | EDII | PTYWCGLFGK | ELISA | - | Non-neutralizing | 34267374 |
| **1100** | ZWT.4 | ARITVVAEDSYFDY | DENV-2 | EDII | PTSWCGLFGK | ELISA | - | Non-neutralizing | 34267374 |
| **1101** | ZWT.4 | ARITVVAEDSYFDY | DENV-3 | EDII | PTVWCGLFGK | ELISA | - | Non-neutralizing | 34267374 |
| **1102** | ZWT.4 | ARITVVAEDSYFDY | DENV-4 | EDII | PTYWCGLFGK | ELISA | - | Non-neutralizing | 34267374 |
| **1103** | ZWT.5 | ARLIYGGGHMDY | DENV-2 | EDII | PTSWCGLFGK | ELISA | - | Non-neutralizing | 34267374 |
| **1104** | ZWT.5 | ARLIYGGGHMDY | DENV-3 | EDII | PTVWCGLFGK | ELISA | - | Non-neutralizing | 34267374 |
| **1105** | ZWT.5 | ARLIYGGGHMDY | DENV-4 | EDII | PTYWCGLFGK | ELISA | - | Non-neutralizing | 34267374 |
| **1106** | ZWT.6 | ARHYRYYLDY | DENV-2 | EDII | PTSWCGLFGK | ELISA | - | Non-neutralizing | 34267374 |
| **1107** | ZWT.6 | ARHYRYYLDY | DENV-3 | EDII | PTVWCGLFGK | ELISA | - | Non-neutralizing | 34267374 |
| **1108** | ZWT.6 | ARHYRYYLDY | DENV-4 | EDII | PTYWCGLFGK | ELISA | - | Non-neutralizing | 34267374 |

Dash symbol (-) representing for no binding detected antibody.

**Supplemental Table 2: Description of sequence-based feature descriptors.**

| **Feature** | **Properties** | **Description** | **Size** | **Reference** |
| --- | --- | --- | --- | --- |
| AAC | Amino Acid Composition | calculates the frequency of each natural amino acid type  (i.e., “ACDEFGHIKLMNPQRSTVWY”). | 20 |  |
| AACPCP | Amino Acid Composition and Physico-Chemical Properties | calculates the frequency of physicochemical properties in a protein sequence based on amino acid composition (i.e., “charged, aliphatic, aromatic, polar, neutral, hydrophobic, positively charged, negatively charged, tiny, small, and large”) | 11 |  |
| AAINDEX | Amino acid indices | Amino acid indices, for representing biochemical reactions. | - | Kawashima et al. (2008) |
| ACID | Acidity | calculate the acidic properties (i.e., “acidic, basic, or neutral) | 3 |  |
| CHARGE | Charge | calculate the charge properties (i.e., “positive, neutral, or negative”) | 3 |  |
| CKSAAGP | Composition of k-Spaced Amino Acid Group Pairs | calculates the frequency of amino acid group pairs separated by any k residues (the default maximum value of k is set as 5). Taking k = 0 as an example, there are 25 0-spaced group pairs (i.e., g1g1, g1g2, g1g3, … g5g5) | 150 | Chen et al. (2013) |
| DHP | Detailed Hydrophobic (H)-Hydrophilic (P) | calculate the DHP properties  (i.e., “non-polar, uncharged-polar, positive-polar, or negative-polar class”) | 4 |  |
| DPC | Di-Peptide Composition | calculate number of dipeptides represented by amino acid types (i.e., AA, AC, AD, YY.), gives 400 descriptors | 400 |  |
| EAAC | Enhanced Amino Acid Composition | calculates the AAC based on the sequence window of fixed length (the default value is 5) that continuously slides from the N- to C-terminus of each peptide and can be usually applied to encode the peptides with an equal length | - | Chen et al. (2018) |
| EGAAC | Enhanced Grouped Amino Acid Composition | calculates Grouped Amino Acid Composition in windows of fixed length (default is 5) continuously sliding from the N- to C-terminal of each peptide and is usually applied to peptides with an equal length | 5 |  |
| POLAR | Polarity/acidity | calculate the polarity and acidity properties | 5 |  |
| SECOND | Secondary structure | calculate the secondary structure properties  (i.e., “helix, strand, or coil”) | 3 |  |

**Supplemental Table 3: Description of atom-based features descriptors.**

| **Feature** | **Properties** | **Description** | **Size** | **Reference** |
| --- | --- | --- | --- | --- |
| Atom type |  | calculate number of atoms: C, H, N, O, S and unknown | 6 |  |
| Aromaticity |  | Indicates whether an amino acid is aromatic or not | 1 |  |
| Degree |  | indicates number of the directly bonded neighbors | 11 |  |
| Formal charge |  | calculate the formal charge on the atoms in an amino acid | 1 |  |
| Hybridization |  | indicates *sp, sp^2^, sp^3^, sp^3^d, and sp^3^d^2^* | 5 |  |
| Number of radical electrons |  | calculate number of atoms in an amino acid with an unpaired electron | 5 |  |
| Valency |  | indicates the valency of the atom in an amino acid | 6 |  |

**Supplemental Table 4: Description of fingerprint-based feature descriptors.**

| **Feature** | **Property** | **Description** | **Size** | **Reference** |
| --- | --- | --- | --- | --- |
| AP2D | 2D atom pair count | Presence of atom pairs at various topological distances | 780 | Carhart, Smith & Venkataraghavan (1985) |
| CDK | CDK | Fingerprint of length 1024 and search depth of 8 | 1,024 | Steinbeck et al. (2003) |
| CDKExt | CDK extended | Extends the fingerprint with additional bits describing ring Steinbeck et al. (2003) features | 1,024 | Steinbeck et al. (2003) |
| CDKGraph | CDK graph only | A special version that considers only the connectivity and Steinbeck et al. (2003) not bond order | 1,024 | Steinbeck et al. (2003) |
| MACCS | MACCS | Binary representation of chemical features | 166 | Durant et al. (2002) |
| PubChem | PubChem | Binary representation of substructures defined by PubChem | 881 | NCBI (2009) |
| Estate | E-state | Electrotopological state atom types | 79 | Hall & Kier (1995) |
| KR | Klekota–Roth | Presence of chemical substructures | 4,860 | Klekota & Roth (2008) |
| FP4 | Substructure | Presence of SMARTS patterns for functional groups | 307 | Laggner (2005) |
| FP4C | Substructure count | Count of SMARTS patterns for functional groups | 307 | Laggner (2005) |
| Circle | Circular | Functionally equivalent to the extended-connectivity fingerprints (ECFP) -2/4/6: features are descriptions of the neighborhood of the atoms up to a certain distance or radius X and the functional class version and (FCFP) -2/4/6 fingerprints | 1,024 | Rogers & Hahn (2010) |
| Hybrid | Hybridization |  | 1,024 | Steinbeck et al. (2003) |

**Supplemental Table 5: Hyperparameter searching**

| **Method** | **Parameters** | **Range of parameters** |
| --- | --- | --- |
| ET | n_estimators | [20, 50, 100] |
| LN | Cost | [2^0^–2^5^] in log_2_ steps |
| LR | Cos | [0.001, 0.01, 0.1, 1, 10, 100] |
| MLP | hidden_layer_sizes | [20, 50, 100] |
| SVM | Cost | [2^0^–2^5^] in log_2_ steps |
| RF | n_estimators | [20, 50, 100] |
| XGB | n_estimators | [20, 50, 100] |

**Supplemental Table 6: ML performances using sequence-based feature encoding method.**

| **ML model** | **10-fold cross validation** | | | | |  | **Independent test** | | | | |
| --- | --- | --- | --- | --- | --- | --- | --- | --- | --- | --- | --- |
|  | **ACC** | **Sn** | **Sp** | **MCC** | **AUC** |  | **ACC** | **Sn** | **Sp** | **MCC** | **AUC** |
| SVM | 0.5374 | 0.814 | 0.271 | 0.1008 | 0.5118 |  | 0.5450 | 0.801 | 0.262 | 0.0667 | 0.5262 |
| LN | 0.5374 | 0.814 | 0.271 | 0.1008 | 0.5054 |  | 0.5450 | 0.801 | 0.262 | 0.0667 | 0.5281 |
| RF | 0.5295 | 0.820 | 0.248 | 0.0824 | 0.5306 |  | 0.5450 | 0.801 | 0.262 | 0.0667 | 0.5262 |
| ET | 0.5262 | 0.837 | 0.227 | 0.0779 | 0.5307 |  | 0.5360 | 0.805 | 0.231 | 0.0438 | 0.5570 |
| XGB | 0.5239 | 0.832 | 0.227 | 0.0725 | 0.5259 |  | 0.5360 | 0.805 | 0.231 | 0.0438 | 0.5570 |
| MLP | 0.5284 | 0.832 | 0.236 | 0.0845 | 0.5236 |  | 0.5140 | 0.186 | 0.885 | 0.099 | 0.5670 |
| 1NN | 0.5136 | 0.789 | 0.231 | 0.0299 | 0.5102 |  | 0.5270 | 0.744 | 0.293 | 0.0288 | 0.5129 |
| DT | 0.5239 | 0.832 | 0.227 | 0.0725 | 0.5251 |  | 0.5360 | 0.805 | 0.231 | 0.044 | 0.5570 |
| LR | 0.5284 | 0.827 | 0.239 | 0.0797 | 0.5339 |  | 0.5450 | 0.801 | 0.262 | 0.0667 | 0.5561 |
| PLS | 0.5147 | 0.766 | 0.291 | 0.0687 | 0.525 |  | 0.5540 | 0.839 | 0.231 | 0.088 | 0.5850 |

**Supplemental Table 7: ML performances using atom-based feature encoding method.**

| **ML model** | **10-fold cross validation** | | | | |  | **Independent test** | | | | |  |
| --- | --- | --- | --- | --- | --- | --- | --- | --- | --- | --- | --- | --- |
|  | **ACC** | **Sn** | **Sp** | **MCC** | **AUC** |  | **ACC** | **Sn** | **Sp** | **MCC** | **AUC** | |
| SVM | 0.6784 | 0.653 | 0.711 | 0.3615 | 0.7177 |  | 0.7207 | 0.661 | 0.789 | 0.4508 | 0.7873 | |
| LN | 0.6682 | 0.603 | 0.734 | 0.3371 | 0.696 |  | 0.6847 | 0.585 | 0.798 | 0.3891 | 0.7479 | |
| RF | 0.737 | 0.737 | 0.740 | 0.4738 | 0.8047 |  | 0.7027 | 0.670 | 0.740 | 0.4095 | 0.8073 | |
| ET | 0.7381 | 0.751 | 0.729 | 0.4781 | 0.8076 |  | 0.7252 | 0.712 | 0.740 | 0.4513 | 0.8116 | |
| XGB | 0.7574 | 0.748 | 0.766 | 0.5113 | 0.8237 |  | 0.6802 | 0.610 | 0.760 | 0.372 | 0.8052 | |
| MLP | 0.6694 | 0.635 | 0.703 | 0.3354 | 0.7277 |  | 0.7207 | 0.602 | 0.856 | 0.4686 | 0.8024 | |
| 1NN | 0.7235 | 0.692 | 0.751 | 0.4439 | 0.7219 |  | 0.6892 | 0.644 | 0.740 | 0.3847 | 0.6922 | |
| DT | 0.6897 | 0.718 | 0.668 | 0.3838 | 0.6922 |  | 0.6622 | 0.610 | 0.721 | 0.332 | 0.6657 | |
| LR | 0.6705 | 0.616 | 0.726 | 0.3407 | 0.6962 |  | 0.6847 | 0.636 | 0.740 | 0.3765 | 0.7381 | |
| PLS | 0.6411 | 0.601 | 0.683 | 0.2837 | 0.6834 |  | 0.6712 | 0.602 | 0.750 | 0.3538 | 0.7189 | |

**Supplemental Table 8: ML performances using fingerprint-based feature encoding method.**

| **ML model** | **10-fold cross-validation test** | | | | |  | **Independent test** | | | | |  |
| --- | --- | --- | --- | --- | --- | --- | --- | --- | --- | --- | --- | --- |
|  | **ACC** | **Sn** | **Sp** | **MCC** | **AUC** |  | **ACC** | **Sn** | **Sp** | **MCC** | **AUC** | |
| SVM | 0.8092 | 0.788 | 0.831 | 0.6179 | 0.8679 |  | 0.7793 | 0.754 | 0.808 | 0.5609 | 0.8519 | |
| LN | 0.7449 | 0.724 | 0.764 | 0.4868 | 0.8098 |  | 0.6847 | 0.627 | 0.750 | 0.3782 | 0.7486 | |
| RF | 0.7911 | 0.769 | 0.814 | 0.5827 | 0.8542 |  | 0.7568 | 0.729 | 0.789 | 0.5164 | 0.8327 | |
| ET | 0.7844 | 0.782 | 0.787 | 0.5673 | 0.8444 |  | 0.7432 | 0.729 | 0.760 | 0.4875 | 0.8249 | |
| XGB | 0.8149 | 0.791 | 0.842 | 0.6298 | 0.8788 |  | 0.8018 | 0.788 | 0.817 | 0.6043 | 0.8849 | |
| MLP | 0.7517 | 0.731 | 0.769 | 0.5024 | 0.815 |  | 0.7748 | 0.746 | 0.808 | 0.5526 | 0.8575 | |
| 1NN | 0.745 | 0.707 | 0.781 | 0.4886 | 0.7439 |  | 0.6667 | 0.627 | 0.712 | 0.3387 | 0.6693 | |
| DT | 0.7337 | 0.689 | 0.772 | 0.4622 | 0.7305 |  | 0.7432 | 0.703 | 0.789 | 0.4916 | 0.7459 | |
| LR | 0.7584 | 0.741 | 0.775 | 0.5139 | 0.8252 |  | 0.7477 | 0.695 | 0.808 | 0.5033 | 0.8374 | |
| PLS | 0.6388 | 0.608 | 0.668 | 0.2754 | 0.7079 |  | 0.7072 | 0.670 | 0.750 | 0.4193 | 0.7654 | |

**Supplemental Table 9: List of top 30 molecular fingerprints and their corresponding descriptions utilized in ML**

**.**

| **Fingerprints** | **Description** |
| --- | --- |
| FP4C1 | Primary carbon |
| FP4C2 | Secondary carbon |
| FP4C3 | Tertiary carbon |
| FP4C12 | Alcohol |
| FP4C13 | Primary alcohol |
| FP4C14 | Secondary alcohol |
| FP4C23 | Amine |
| FP4C36 | Alkylthiol |
| FP4C84 | Carboxylic acid |
| FP4C88 | Carboxylic acid derivative |
| FP4C98 | Amide |
| FP4C99 | Primary amide |
| FP4C100 | Secondary amide |
| FP4C184 | Heteroaromatic ring |
| FP4C274 | Aromatic ring |
| FP4C295 | C ONS bond |
| FP4C300 | 1,3-Tautomerizable |
| FP4C302 | Rotatable bond |
| FP4C307 | Chiral center specified |
| MACCS129 | sulfur-sulfur (S-S) bond |
| CDK622 | CDK bit 622 |
| Circle162 | ECFP bit 162 |
| Circle174 | ECFP bit 174 |
| Circle391 | ECFP bit 391 |
| Circle477 | ECFP bit 477 |
| Circle496 | ECFP bit 496 |
| Circle59 | ECFP bit 59 |
| Circle657 | ECFP bit 657 |
| Circle892 | ECFP bit 892 |
| Circle981 | ECFP bit 981 |

**Supplemental Table 10: Data of 1B3B9 and 28 NAbs after screening with ML and MD methods.**

| **Antibody name** | **CDR-H3 sequence** | **ML screening**  **(XGB model)** | | **MD screening**  **(Amber 16)** | |
| --- | --- | --- | --- | --- | --- |
|  |  | **Confident score** | **Ramachandran favoured, QMEAN  Z-score** | **Averaged RMSD (Å)** | **Averaged end-to-end distance (Å)** |
| 1B3B9  (template) | TTLSGYSADWPEDY | ND | 96.73%, 0.25 | 2.94 ± 0.32 | 39.22 ± 4.33 |
| 1B3B9-V1 | TTLFGRVADWPEDY | 0.9905 | 96.26%, -0.04 | 3.31 ± 0.97 | 44.62 ± 4.65 |
| 1B3B9-V2* | TTLFGEVADWPEDY | 0.9901 | 96.26%, -0.04 | 2.89 ± 0.46 | 38.08 ± 4.88 |
| 1B3B9-V3 | TTLFGKVADWPEDY | 0.9905 | 96.50%, 0.03 | 3.34 ± 0.93 | 52.38 ± 7.75 |
| 1B3B9-V4 | TTLWGRVADWPEDY | 0.9901 | 96.26%, 0.06 | 2.90 ± 0.51 | 45.63 ± 3.73 |
| 1B3B9-V5 | TTLWGKVADWPEDY | 0.9901 | 96.50%, -0.06 | 3.76 ± 1.09 | 52.14 ± 3.99 |
| 1B3B9-V6 | TTLYGRVADWPEDY | 0.9905 | 96.50%, -0.15 | 3.62 ± 0.81 | 47.30 ± 5.24 |
| 1B3B9-V7 | TTLYGEVADWPEDY | 0.9901 | 96.50%, -0.10 | 3.32 ± 0.69 | 54.08 ± 6.36 |
| 1B3B9-V8 | TTLYGKVADWPEDY | 0.9905 | 93.98%, -2.24 | 11.23 ± 0.53 | 40.82 ± 10.60 |
| 1B3B9-V9 | TTLDGRSADWPPDY | 0.9904 | 96.26%, 0.02 | 4.08 ± 0.92 | 55.37 ± 6.37 |
| 1B3B9-V10 | TTLDGESADWPPDY | 0.9904 | 96.50%, 0.01 | 4.43 ± 0.67 | 47.13 ± 9.26 |
| 1B3B9-V11 | TTLDGKSADWPPDY | 0.9904 | 93.98%, -2.24 | 11.23 ± 0.53 | 36.08 ± 12.92 |
| 1B3B9-V12 | TTLFGRSADWPPDY | 0.9903 | 96.73%, -0.07 | 3.67 ± 0.77 | 41.86 ± 4.35 |
| 1B3B9-V13 | TTLFGESADWPPDY | 0.9903 | 96.73%, -0.06 | 4.07 ± 0.65 | 52.29 ± 7.78 |
| 1B3B9-V14 | TTLFGKSADWPPDY | 0.9903 | 96.26%, 0.21 | 4.15 ± 0.81 | 54.68 ± 4.47 |
| 1B3B9-V15 | TTLYGRSADWPPDY | 0.9903 | 96.73%, 0.22 | 3.74 ± 0.85 | 49.16 ± 6.40 |
| 1B3B9-V16 | TTLYGESADWPPDY | 0.9903 | 96.03%, 0.16 | 3.54 ± 0.73 | 35.48 ± 3.12 |
| 1B3B9-V17 | TTLYGKSADWPPDY | 0.9903 | 96.50%, 0.07 | 2.48 ± 0.22 | 46.07 ± 4.54 |
| 1B3B9-V18 | TTLVGRSADWPPDY | 0.9904 | 96.50%, 0.03 | 3.87 ± 0.84 | 42.58 ± 6.80 |
| 1B3B9-V19 | TTLVGESADWPPDY | 0.9904 | 96.73%, 0.04 | 2.95 ± 0.43 | 44.14 ± 7.65 |
| 1B3B9-V20 | TTLVGKSADWPPDY | 0.9904 | 95.79%, 0.07 | 3.89 ± 0.49 | 40.47 ± 4.63 |
| 1B3B9-V21* | TTLFGYPPDWPEDY | 0.9900 | 95.79%, 0.12 | 2.65 ± 0.36 | 38.77 ± 3.44 |
| 1B3B9-V22 | TTLYGYPPDWPEDY | 0.9900 | 96.50%, -0.00 | 3.38 ± 0.42 | 35.27 ± 2.13 |
| 1B3B9-V23 | TTLHGYGAVWPEDY | 0.9902 | 96.50%, -0.02 | 4.40 ± 1.29 | 54.34 ± 5.83 |
| 1B3B9-V24 | TTLHGYVADPPEDY | 0.9902 | 96.50%, 0.03 | 3.65 ± 0.63 | 33.90 ± 5.55 |
| 1B3B9-V25 | TTLSGEVADWPPDY | 0.9902 | 96.73%, 0.10 | 2.51 ± 0.22 | 56.67 ± 5.25 |
| 1B3B9-V26 | TTLSGESADWPPDV | 0.9907 | 96.03%, 0.17 | 2.85 ± 0.33 | 48.14 ± 6.92 |
| 1B3B9-V27 | TTLSGYPPDWPEVY | 0.9900 | 96.26%, 0.12 | 3.74 ± 0.64 | 34.45 ± 3.25 |
| 1B3B9-V28 | TTLSGYPPDWPEDV | 0.9900 | 96.38%, 0.17 | 4.83 ± 0.31 | 42.66 ± 9.65 |

ND; not determined, Red letters represent mutated residues of antibody variants from antibody template (1B3B9).

* Outstanding candidates

# **Supplemental Table 11: Numbers of atom-to-atom contacts between 1B3B9 and DENV-1 to DENV-4 E proteins.**

| DENV 1-E | H chain contacts | L chain contacts | Total contacts | DENV 2-E | H chain contacts | L chain contacts | Total contacts | DENV 3-E | H chain contacts | L chain contacts | Total contacts | DENV 4-E | H chain contacts | L chain contacts | Total contacts |
| --- | --- | --- | --- | --- | --- | --- | --- | --- | --- | --- | --- | --- | --- | --- | --- |
| W101 | G102(2) ^a^, Y103(51), S104(18), D105(11) |  | 82 | W101 | G102(17), Y103(29), S104(46) |  | 92 | R99 |  | W59(1) | 1 | R99 | G26(1) |  | 1 |
| G104 | G102(20) |  | 20 | N103 | Q1(1), G102(8) |  | 9 | W101 | W106(67) | S58(3) | 70 | W101 | Q23(1), S25(5) |  | 6 |
| C105 | G102(33), Y103(17) |  | 50 | G104 | Q1(3), G102(11) |  | 14 | N103 |  | S58(23), T60(11), H61(31), A62(17) | 82 | N103 | A24(7), SS25(39), G26(18), V76(1), S77(6) |  | 71 |
| G106 | Y32(8), G102(17), Y103(38) |  | 63 | C105 | Q1(10), G26(9), G102(17), Y103(12) |  | 48 | G104 |  | S58(7), W59(3), H61(1) | 11 | G104 | Q1(7), SS25(21), G26(29) |  | 57 |
| L107 | Y103(29) |  | 29 | G106 | V2(6), G26(16), Y27(19), G102(32), Y103(48) |  | 121 | C105 | S104(1) | S58(23), W59(2) | 26 | C105 | Q1(20), H3(23), S25(2) |  | 45 |
| F108 | Y103(10) |  | 10 | L107 | G26(4), Y27(3), P28(15), Y103(14) |  | 36 | G106 | G102(22), S104(7), E108(5) | S58(17) | 51 | G106 | Q1(20), H3(20) |  | 40 |
|  |  |  |  | F108 | Y103(50), S104(3) |  | 53 | L107 | G102(29), Y103(22), S104(4), 106(2) |  | 57 |  |  |  |  |
|  |  |  |  |  |  |  |  | F108 | Y103(12), S104(8) |  | 20 |  |  |  |  |
| Total | 258 | 0 | 254 | Total | 373 | 0 | 373 | Total | 179 | 139 | 318 | Total | 220 | 0 | 220 |

^a^Numbers represent the number of atom-to-atom contacts analyzed by the contact command of AMBER16 (The distance cutoff is 4.5 Å.).

E; Envelope, H; Heavy chain, L; Light chain

**Supplemental Table 12: Numbers of** **atom-to-atom contacts between 1B3B9_V21 and DENV-1 to DENV-4 E proteins.**

| DENV 1-E | H chain contacts | L chain contacts | Total contacts | DENV 2-E | H chain contacts | L chain contacts | Total contacts | DENV 3-E | H chain contacts | L chain contacts | Total contacts | DENV 4-E | H chain contacts | L chain contacts | Total contacts |
| --- | --- | --- | --- | --- | --- | --- | --- | --- | --- | --- | --- | --- | --- | --- | --- |
| R99 | Q1(21) ^a^, S25(2), G26(2) |  | 25 | W101 | W106 (101) | K56 (8), H57 (4), S58 (34), W59 (2), T60 (2) | 151 | W101 | P104(26), W106(64) | S58(6) | 96 | W101 | P28(57), T30(2), R31(64), Y32(8), L100(4) |  | 135 |
| W101 | P103(49), P104(1), D105(11) |  | 61 | N103 |  | T60 (7), H61 (19), A62 (29) | 55 | G104 |  | S58(4) | 4 | G102 | R31(22) |  | 7 |
| N103 | Q1(5), V2(1), L100(1), G102(2), E108(1), Y110(1) |  | 11 | G104 |  | S58 (4), T60 (1), H61 (1) | 6 | C105 | P104(4) | S58(20), W59(1) | 25 | N103 | R31(5) |  | 5 |
| G104 | Q1(24), G26(1), Y27(1), L100(1) |  | 27 | C105 | P104 (2) | S58 (18), W59 (10) | 30 | G106 | G102(26), P104(14), W106(1) E108(6) | S58(18), W59(2) | 67 | G104 | R31(26), Y32(22) |  | 48 |
| C105 | Q1(54), V2(2) |  | 56 | G106 | G102 (10),  P104 (11),  W106 (5),  E108 (6) | S58 (38), W59 (7) | 77 | L107 | G102(26), P103(21), P104(45) |  | 92 | C105 | Y32(14), L100(8), F101(3), D105(15) |  | 40 |
| G106 | Q1(14), V2(24), G26(12), Y27(1) |  | 51 | L107 | G102 (19), P103 (5),  P104 (39) |  | 63 | F108 | G102(2), P103(22), P104(11) |  | 35 | G106 | L100(5), F101(4), G102(3), P103(16), P104(4), D105(37) |  | 69 |
| L107 | G26(12), Y27(1), P28(1), L100(1) |  | 15 | F108 | P104 (65) |  | 65 |  |  |  |  | L107 | P103(15), P104(6), D105(31) |  | 52 |
|  |  |  |  |  |  |  |  |  |  |  |  | F108 | D105(1) |  | 1 |
| Total | 246 | 0 | 246 | Total | 263 | 184 | 447 | Total | 268 | 51 | 319 | Total | 357 | 0 | 357 |

^c^ analyzed by the contact command of AMBER16 (The distance cutoff is 4.5 Å.).

E; Envelope, H; Heavy chain, L; Light chain

**Supplemental Table 13: Numbers of atom-to-atom contacts between 1B3B9_V21 against ZIKV and JEV E proteins.**

| ZIKV-E | H chain contacts | L chain contacts | Total contacts | JEV-E | H chain contacts | L chain contacts | Total contacts |
| --- | --- | --- | --- | --- | --- | --- | --- |
| R99 | D105(13) |  | 13 | W101 | W106(6) |  | 6 |
| W101 | P103(9),  P104(31),  D105(6) |  | 46 | G102 | W106(8) |  | 8 |
| G102 | P104(1) |  | 1 | N103 | W106(6) |  | 6 |
| N103 | D105(1) |  | 1 | G104 | P104(15),  W106(30) |  | 45 |
| G104 | Y102(4),  P104(17),  W106(2) |  | 23 | C105 | P104(12),  W106(5) |  | 17 |
| C105 | Y102(4),  P104(7),  W106(3) |  | 23 | G106 | P104(3),  D105(5) |  | 8 |
| G106 | P103(2),  W106(7) |  | 9 |  |  |  |  |
| L107 | W106(3) |  | 3 |  |  |  |  |
| Total | 119 | 0 | 119 | Total | 93 |  | 93 |

^a^ analyzed by UCSF Chimera using contact command (The distance cutoff is 4.5 Å.).

E; Envelope, H; Heavy chain, L; Light chain
